# Mutation, methylation, and gene expression profiles in dup(1q)-positive pediatric B-cell precursor acute lymphoblastic leukemia

**DOI:** 10.1038/s41375-018-0092-2

**Published:** 2018-03-12

**Authors:** Rebeqa Gunnarsson, Sebastian Dilorenzo, Kristina B Lundin-Ström, Linda Olsson, Andrea Biloglav, Henrik Lilljebjörn, Marianne Rissler, Per Wahlberg, Anders Lundmark, Anders Castor, Mikael Behrendtz, Thoas Fioretos, Kajsa Paulsson, Anders Isaksson, Bertil Johansson

**Affiliations:** 1 Division of Clinical Genetics, Department of Laboratory Medicine, Lund University, Lund, Sweden; 2 Array and Analysis Facility, Department of Medical Sciences, Uppsala University, Uppsala, Sweden; 3 National Bioinformatics Infrastructure Sweden, Science for Life Laboratory, Uppsala University, Uppsala, Sweden; 4 Department of Clinical Genetics and Pathology, Division of Laboratory Medicine, Lund, Sweden; 5 Department of Medical Sciences, Molecular Medicine and Science for Life Laboratory, Uppsala University, Uppsala, Sweden; 6 Department of Pediatrics, Skåne University Hospital, Lund, Sweden; 7 Department of Pediatrics, Linköping University Hospital, Linköping, Sweden

## Abstract

High-throughput sequencing was applied to investigate the mutation/methylation patterns on 1q and gene expression profiles in pediatric B-cell precursor acute lymphoblastic leukemia (BCP ALL) with/without (w/wo) dup(1q). Sequencing of the breakpoint regions and all exons on 1q in seven dup(1q)-positive cases revealed non-synonymous somatic single nucleotide variants (SNVs) in *BLZF1*, *FMN2*, *KCNT2*, *LCE1C*, *NES*, and *PARP1*. Deep sequencing of these in a validation cohort w (*n* = 17)/wo (*n* = 94) dup(1q) revealed similar SNV frequencies in the two groups (47% vs. 35%; *P* = 0.42). Only 0.6% of the 36,259 CpGs on 1q were differentially methylated between cases w (*n* = 14)/wo (*n* = 13) dup(1q). RNA sequencing of high hyperdiploid (HeH) and t(1;19)(q23;p13)-positive cases w (*n* = 14)/wo (*n* = 52) dup(1q) identified 252 and 424 differentially expressed genes, respectively; only seven overlapped. Of the overexpressed genes in the HeH and t(1;19) groups, 23 and 31%, respectively, mapped to 1q; 60-80% of these encode nucleic acid/protein binding factors or proteins with catalytic activity. We conclude that the pathogenetically important consequence of dup(1q) in BCP ALL is a gene-dosage effect, with the deregulated genes differing between genetic subtypes, but involving similar molecular functions, biological processes, and protein classes.

## Introduction

Gain of 1q through a duplication or an unbalanced translocation (both are here denoted “dup(1q)”) is found by chromosome banding analysis in ~5% of pediatric B-cell precursor acute lymphoblastic leukemia (BCP ALL) cases[1, 2], but the frequency increases quite substantially if single nucleotide polymorphism array (SNP-A) analyses are performed[3, 4]. The incidence of dup(1q) varies according to genetic subtype, with 1q gain being particularly common in cases with t(1;19)(q23;p13)/*TCF3-PBX1* fusion (~60%), most often as a consequence of the unbalanced der(19)t(1;19), or with high hyperdiploidy (HeH; ~20%); more rarely, dup(1q) is present in t(12;21)(p13;q22)/*ETV6*-*RUNX1*-positive or B-other cases[2, 4–7]. However, gain of 1q is not restricted to BCP ALL. In fact, dup(1q) is one of the most common genomic imbalances in human neoplasia, such as multiple myeloma, various types of B-cell lymphoma, carcinomas of the breast, colon, and lung, malignant melanoma, and Wilms' tumor[2, 8–15]. The high frequency of 1q gain strongly suggests that it plays an important pathogenetic role in tumorigenesis in general, perhaps by conferring a proliferative advantage, as indicated by a study of growth patterns of dup(1q)-positive and -negative chronic lymphocytic leukemia clones in nude mice[16]. Such a proliferative advantage is a possible explanation for the worse prognosis of Wilms' tumors and multiple myelomas with 1q gains[8, 17, 18].

Genomic gains are generally associated with gene-dosage effects resulting in overexpression of some of the duplicated/amplified genes[19, 20]. However, considering the high frequency of 1q gain in neoplasia, surprisingly few studies have investigated its effect on gene expression or ascertained pathogenetically important target genes within the duplicated segments. In hematologic malignancies, gain of 1q has been associated with overexpression of the *CKS1B* gene in 1q21 in multiple myeloma [17, 18] and with upregulation of *B4GALT3* (1q23), *DAP3* (1q22), *RGS16* (1q25)*, TMEM183A* (1q32), and *UCK2* (1q24) in a few dup(1q)-positive HeH cases[21]. This notwithstanding, a gene-dosage effect may not be the only functional consequence of dup(1q)—it may also be associated with DNA methylation changes and/or gene mutations on 1q, but this has not been addressed in previous studies of dup(1q)-positive malignancies.

To ascertain the functional consequences of dup(1q) in pediatric BCP ALL, we compared, using several types of next-generation sequencing, the mutation and methylation patterns on 1q and global gene expression profiles between cases with/without (w/wo) 1q gain.

## Materials and methods

### Patients

Twenty-seven dup(1q)-positive, all identified by SNP-A analysis, and 132 dup(1q)-negative pediatric BCP ALL cases were investigated (Supplementary Tables 1 and 2). Based on the SNP-A data, the smallest dup(1q)-positive clone included had a frequency of ~25% (case 6; Supplementary Tables 1). A flowchart outlining the number of cases included in the various analyses is presented in Supplementary Figure 1.

The 27 cases with 1q gain consisted of 16 HeH, eight t(1;19), one t(12;21), and two B-other cases. Targeted deep sequencing of 1q was performed on seven cases, Truseq custom amplicon (TCA) analysis on 17, bisulfite sequencing (BS-Seq) on 15, and RNA sequencing (RNA-Seq) on 14 cases (Supplementary Table 1). The 132 dup(1q)-negative cases, used as a validation or reference cohort in the TCA (*n* = 94), BS-Seq (*n* = 15), and RNA-Seq (*n* = 52) analyses, comprised 51 HeH, six t(1;19), 32 t(12;21), seven 11q23-rearranged, four t(9;22)(q34;q11), one low hypodiploid, and 31 B-other cases (Supplementary Table 2). The cases with t(1;19), t(9;22), and t(12;21) were all confirmed to be positive for the *TCF3*-*PBX1*, *BCR*-*ABL1*, and *ETV6*-*RUNX1* fusions, respectively, by fluorescence in situ hybridization (FISH) or reverse-transcription polymerase chain reaction analysis, whereas the cases with 11q23 rearrangements were all confirmed to be *KMT2A*-rearranged by FISH or Southern blot analysis.

The study was approved by the Research Ethics Committee of Lund University (DNR 2016/18) and informed consent for the analyses was obtained according to the Declaration of Helsinki.

### Single nucleotide polymorphism array analysis

The SNP-A analyses and original SNP data have previously been reported[4, 22]. The SNP-A systems HumanOmni1-Quad or Human1M-Duo (Illumina, San Diego, CA, USA), covering >1,000,000 SNPs, were used. The analyses were performed according to the manufacturer’s instructions and the B-allele frequencies and the log2 ratios were ascertained by the Genome studio v2011.1 software (Illumina), extracting probe positions from the GRCh37 genome build.

### Targeted deep sequencing of 1q

The SureDesign tool was applied to select probes for the DNA library preparation kit (Agilent Technologies, Santa Clara, CA, USA). Two probe sets were used: (1) probes covering the 1q breakpoint regions at chr1:142,535,430-150,580,000; chr1:155,065,160-156,045,662; chr1:164,619,900-164,875,964; chr1:186,307,000-186,961,000; chr1:189,450,000-190,872,000; chr1:197,566,843-197,843,204; chr1:215,136,000-215,400,000; chr1:224,625,000-224,800,000; chr1:226,000,000-226,055,000; and chr1:249,000,000-249,250,621; and (2) probes covering all exons on 1q. This resulted in 220,160 probes with a total size of 13.755 Mb.

Seven paired diagnostic/remission samples were included in the library preparation and sequenced on HiSeq 2000 (Illumina) at SciLifeLab, Uppsala, Sweden. The Burrows–Wheeler aligner was used for alignment of the DNA sequences to the human reference genome (Hg19)[23]. Variant calling was performed using MuTect/1.1.5[24], candidate mutations were annotated with Annovar[25], and structural variant calling for breakpoint mapping was performed with LUMPY/0.2.13[26]. The results from LUMPY were processed with SVTYPER (https://github.com/hall-lab/svtyper) [27] to generate descriptive genotype information, and custom scripts were used to identify candidate somatic structural variants. The filtering criteria used for candidate detection were A0 > 4 in the leukemic samples and A0 = 0 in the matched remission sample for each variant.

### Truseq custom amplicon analysis

Based on the single nucleotide variants (SNVs) identified by targeted deep sequencing of 1q, the *BLZF1* (basic leucine zipper nuclear factor 1 at 1q24.2), *FMN2* (formin 2; 1q43), *KCNT2* (potassium sodium-activated channel subfamily T member 2; 1q31.3), *LCE1C* (late cornified envelope 1C; 1q21.3), *NES* (nestin; 1q23.1), and *PARP1* (poly(ADP-ribose) polymerase 1; 1q42.12) genes were selected for TCA analysis of a larger patient cohort w/wo 1q gains. Probes targeting all exons within these genes were constructed with DesignStudio (Illumina). In total, 111 diagnostic samples w (*n* = 17)/wo (*n* = 94) dup(1q) and 79 paired remission samples from cases w (*n* = 10)/wo (*n* = 69) dup(1q) at diagnosis were analyzed. The sequence libraries were constructed according to the manufacturer’s instructions and the pooled libraries were sequenced on Nextseq 500 (Illumina). Variant calling was performed with MuTect/1.1.7[24]. In the variant calling of the 32 cases without a remission sample, a reference panel was utilized, created from the available 79 remission samples by calling variants using MuTect/1.1.7, and then merging the variants using CombineVariants from GATK/3.4.0[28–30]. Subsequently, the reference panel was used to identify putative somatic variation in leukemia samples lacking a remission sample. All variants were annotated with Annovar[25].

### Sanger sequencing

For the SNVs selected for verification by Sanger sequencing, forward and reverse primers (Supplementary Table 3) were designed with Primer3 (http://bioinfo.ut.ee/primer3/) and purchased from Termo Fisher Scientific (Waltham, MA, US). ChromasLite 2.6 (Technelysium, South Brisbane, Australia) was used for sequence analysis.

### Bisulfite sequencing

Thirty cases w (*n* = 15)/wo (*n* = 15) 1q gains were bisulfite sequenced. The SureSelect Human Methyl-Seq (Agilent), which targets cancer tissue-specific differentially methylated regions, promoters, CpG islands, shores, and shelves, was used. The library preparations were performed according to the manufacturer’s instructions and the pooled libraries were sequenced on HiSeq 2000 (Illumina) at SciLifeLab. The BS-Seq data were processed through a bioinformatic pipeline developed at SciLifeLab (Supplementary Figure 2). Reference genome ucsc.hg19.fasta (bundle 2.8) was processed with Bismark/0.12.2 to create a suitable human reference genome for alignment of bisulfite-treated reads[31]. Analysis of differentially methylated CpGs was performed using the R package methylKit/0.9.2 and R/3.0-3.2.3[32]. Methylation calls for the cases w/wo dup(1q) were compared for overlapping sites, with each positions coverage required to be in the interval 2 × −50 × . Principal component and hierarchical clustering analyses (PCA and HCA) were performed on CpGs with differential methylation, as ascertained by methylKit/0.9.2. For a site to be considered differentially methylated between the groups w/wo dup(1q), a difference of ≥25% and a *q*-value less than 0.01 were required. Annotations were downloaded from UCSC (assembly hg19) and matched to gene regions, CpG islands, shores, and shelves in the differential methylation data set. Annotation plots of differential methylation in genomic and CpG regions, including both hyper- and hypomethylated sites, were created with methylKit (https://github.com/al2na/methylKit)[32].

### RNA sequencing and gene expression profiling

The RNA-Seq analyses, focusing on fusion genes in 195 pediatric BCP ALL cases, have previously been reported[33]. The RNA-seq data are deposited at the European Genome-phenome Archive under the accession code EGAS00001001795. Qlucore Omics Explorer 3.1 (Qlucore AB, Lund, Sweden) was used for gene expression profiling of HeH cases w (*n* = 7)/wo (*n* = 46) and t(1;19)-positive cases w (*n* = 7)/wo (*n* = 6) 1q gain. Cases w/wo dup(1q) were compared with the paired *t*-test, and a *P*-value of ≤0.01 was used as a cut-off for differentially expressed genes. HCA was performed on the differentially expressed genes in the dup(1q)-positive and -negative HeH and t(1;19) groups. For gene ontology (GO) analysis of the differentially expressed genes, the Panther version 12.0 (http://pantherdb.org/) was used to extract information on their molecular functions, biological processes, and protein classes.

## Results

### Dup(1q) in BCP ALL: near-centromeric breakpoints and whole arm gains

SNP-A analyses of the 27 dup(1q)-positive cases revealed that the 1q gains ranged from 24.4 Mb to 103.9 Mb, with a median size of 97.9 Mb (Supplementary Table 4). One case (#3) had two separate 24.4 Mb and 39.3 Mb duplications, whereas all other cases harbored a single, continuous duplication. The minimal overlapping gain in the HeH subgroup was the 24.4 Mb duplication in case 3, and in the t(1;19) subgroup, the smallest overlapping region was a 84.4 Mb duplication in case 16. In 16 (59%) of the 27 cases and in 13 (81%) of the 16 HeH cases the proximal breakpoints were near-centromeric, occurring in, or proximal to, 1q21.1 (the repetitive nature of the DNA sequence in this region made it impossible exactly to map the breakpoints by SNP-A). In 22 (81%) of the cases, the gains included the telomeric region, i.e., the entire 1q arm distal to the proximal breakpoint was duplicated (Supplementary Figure 3 and Supplementary Tables 1 and 4). In cases 2, 3, 4, and 7 with proximal breakpoints distal to 1q21.1 and/or with distal breakpoints proximal to the telomeric region, the sequence analysis revealed that the breakpoint regions were surrounded by small structural variants that most often were classified as deletions. However, it was not possible to identify the precise locations of the breakpoints.

### Similar somatic mutation frequencies on 1q in cases with/without dup(1q)

Targeted deep sequencing of the breakpoint regions and of all exons (including also intronic flanking sequences) on 1q revealed a total of 231 somatic SNVs in the seven cases analyzed (range, 18 to 74 SNVs per case; Supplementary Table 5). The majority of the SNVs were intergenic (151/231; 65%) and most were novel (144/231; 62%), i.e., not included in dbSNP (https://www.ncbi.nlm.nih.gov/projects/SNP/). Of the 80 intragenic SNVs, 61 (76%) occurred within introns, nine (11%) were located upstream, downstream, or in the 3′ untranslated regions of genes, and ten (12%) were exonic. Of the ten exonic SNVs, three were synonymous and seven non-synonymous. Six of the latter, occurring in the *BLZF1, FMN2, KCNT2, LCE1C*, *NES*, and *PARP1* genes, were detected in 17–66% of the reads and were all verified by Sanger sequencing (Supplementary Table 5). One non-synonymous SNV in the *OR2T34* gene (olfactory receptor family 2 subfamily T member 34 at 1q44) was discarded because it was only present in 4/42 (10%) reads.

The TCA analysis of the *BLZF1, FMN2, KCNT2, LCE1C*, *NES*, and *PARP1* genes in the validation cohort (*n* = 111) revealed SNVs in ≥1 of these genes in 76% (13/17) of the dup(1q)-positive and in 66% (62/94) of the dup(1q)-negative cases (*P* = 0.42; two-tailed Fisher’s exact test) (Supplementary Table 6). When only considering non-synonymous SNVs, the corresponding frequencies were 47% (8/17) and 35% (33/94), respectively (*P* = 0.42).

### Similar methylation patterns on 1q in cases with/without dup(1q)

Of the 30 cases analyzed by BS-Seq, three did not pass the quality score test. Hence, the methylation patterns on 1q could be ascertained in 27 cases. HCA and PCA of the cases w (*n* = 14)/wo (*n* = 13) gain of 1q did not separate these two groups (Supplementary Figure 4). Furthermore, only 201 (0.6%) of the 36,259 CpG sites on 1q were differentially methylated, with 137 (68%) being hypermethylated and 64 (32%) hypomethylated. The distributions of differentially methylated CpGs in promoters, introns, exons, and intergenic regions did not differ between hyper- and hypomethylated CpGs (Supplementary Figure 5).

Comparing the methylation and gene expression data in cases analyzed by both BS-Seq and RNA-Seq, only seven genes displayed both differential methylation of CpGs and differential expression: *RORC* was hypermethylated and underexpressed, whereas the *GALNT2*, *PSMB4*, *SHC1*, *SOX13*, *SSR2*, and *UCK2* genes were hypomethylated and overexpressed in the dup(1q)-positive cases.

### Different deregulated genes in dup(1q)-positive HeH and t(1;19) cases

HCA of the global gene expression profiles as well as of the expression patterns of only genes mapping to 1q in the t(1;19) cases w/wo 1q gain revealed two cluster groups characterized by the presence or absence of dup(1q) (*P* ≤ 0.01), with only one dup(1q)-positive case being grouped together with the non-dup(1q) cases (Supplementary Figure 6a and b). HCA of the global gene expression profiles in the HeH cases also clustered most dup(1q)-positive cases together (Supplementary Figure 6c); such a clustering was not found by HCA of the expression of genes only on 1q in the HeH cases (Supplementary Figure 6d).

**Fig. 1:**
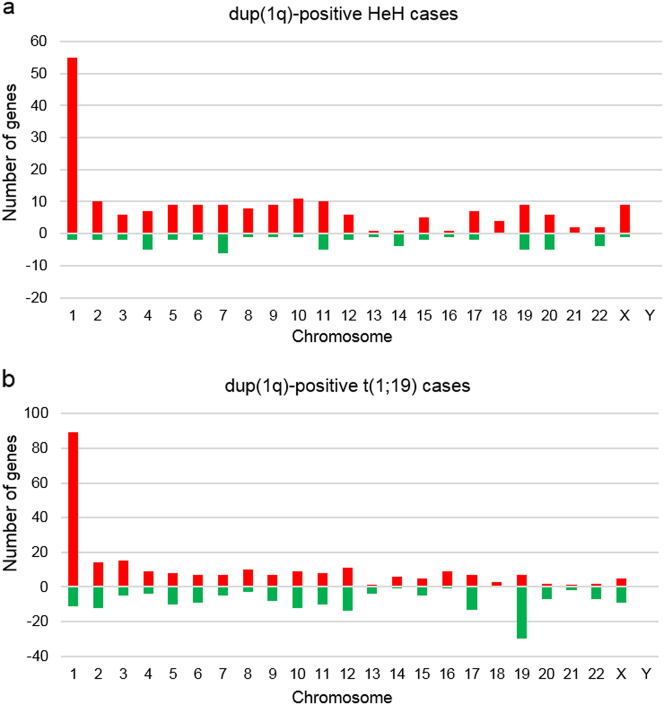
Frequencies of significantly (*P* < 0.01) differentially expressed genes (red denotes upregulated genes whereas green denotes downregulated genes) and their chromosomal locations in dup(1q)-positive pediatric HeH and t(1;19) cases. **a** Gene expression profiling (GEP) analysis of HeH cases w (*n* = 7)/wo (*n* = 46) gain of 1q revealed that 252 genes were differentially expressed between cases w/wo dup(1q): 196 (78%) were overexpressed (46 (23%) of which mapped to 1q) and 56 (22%) were underexpressed. **b** GEP analysis of t(1;19) cases w (*n* = 7)/wo (*n* = 7) gain of 1q showed that 424 genes were differentially expressed between the t(1;19) cases w/wo dup(1q): 242 (57%) were overexpressed (76 (31%) of which mapped to 1q) and 182 (43%) were underexpressed. In the cases with der(19)t(1;19), resulting in loss of distal 19p, 30 genes located on chromosome 19 were downregulated; 16 (53%) of these mapped to the 1.6 Mb region telomeric to the *TCF3* gene at 19p13.3

In the HeH group, 252 genes were differentially expressed between cases w/wo dup(1q); 196 (78%) were overexpressed and 56 (22%) underexpressed (Fig. 1a; Supplementary Table 7). A total of 424 genes were differentially expressed between the t(1;19) cases w/wo dup(1q), of which 242 (57%) were overexpressed and 182 (43%) underexpressed (Fig. 1b; Supplementary Table 8). In the cases with der(19)t(1;19), resulting in loss of distal 19p, 30 genes located on chromosome 19 were downregulated (Fig. 1b); 16 (53%) of these mapped to the 1.6 Mb region telomeric to the *TCF3* gene at 19p13.3, a region comprising only 2.8% of chromosome 19. Of the 252 and 424 differentially expressed genes in the HeH and t(1;19) groups, respectively, only seven overlapped, of which *ELK4* (1q32.1), *GAS5* (1q25.1), *NENF* (1q32.3), and *SNRPE* (1q32.1) were overexpressed and *CAV1* (7q31.2), *POU2AF1* (11q23.1), and *ZNF831* (20q13.32) downregulated in dup(1q)-positive cases.

**Fig. 2:**
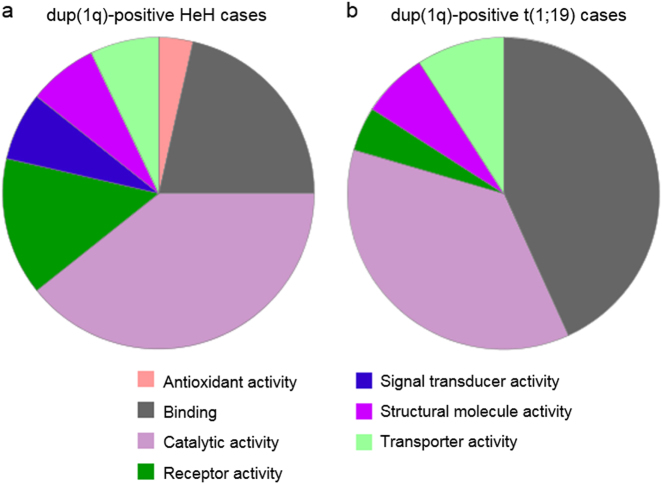
Molecular function ontology classes of genes on 1q upregulated in dup(1q)-positive cases. **a** In the HeH cases, 28 of the 46 upregulated genes on 1q had defined molecular functions, comprising seven classes. **b** In the (1;19) cases, 44 of the 76 overexpressed genes on 1q had defined molecular functions, comprising five ontology classes. Antioxidant activity (GO:0016209); binding (GO:0005488); catalytic activity (GO:0003824); receptor activity (GO:0004872); signal transducer activity (GO:0004871); structural molecule activity (GO:0005198); and transporter activity (GO:0005215)

Among the overexpressed genes in the dup(1q)-positive HeH and t(1;19) groups, 23% (46/196) and 31% (76/242), respectively, were located on 1q (Fig. 1a, b); in comparison, 1,115 (4.8%) of the 23,285 genes included in the analysis were located on 1q. Using Panther for GO analysis, defined molecular functions, biological processes, and protein classes were available for 28, 41, and 37 of the upregulated genes on 1q in the HeH cases and for 44, 74, and 46 of the overexpressed 1q genes in the t(1;19) cases. In both the HeH and t(1;19) groups, the most common molecular functions of the upregulated 1q genes were “binding” (21% and 43%, respectively), comprising nucleic acid binding and protein binding, and “catalytic activity” (39% and 36%, respectively), e.g., hydrolase and transferase activity (Fig. 2a, b). With regard to biological processes of the overexpressed genes in the dup(1q)-positive HeH and t(1;19) cases, cellular and metabolic processes, such as cell communication and primary metabolic activity, were the most frequent (56% and 52%, respectively) (Supplementary Figure 7a and b). Eighteen protein classes were represented among the overexpressed genes, of which 13 were common to both the HeH and t(1;19) groups; the most frequent were receptors/signaling molecules, transcription factors, and nucleic acid binding proteins (Supplementary Figure 8a and b).

## Discussion

The present study—the first to apply several types of next-generation sequencing to ascertain genomic and epigenetic features of dup(1q) in BCP ALL—revealed clustering of near-centromeric breakpoints and gain of the entire chromosome arm in the majority of cases, similar gene mutation frequencies and methylation patterns on 1q in cases w/wo gain of 1q, and distinct gene expression profiles of HeH and t(1;19) cases with dup(1q).

Previous studies of solid tumors as well as of some hematologic malignancies, using chromosome banding, FISH, or SNP-A analyses[12, 13, 21, 34–40], have reported that the proximal breakpoints of dup(1q) in most cases are near-centromeric, often within the satellite II (sat II) domain. In the present study, we identified breakpoints in, or proximal to, 1q21.1 in ~60% of all cases and in ~80% of the HeH cases. This agrees well with prior analyses of 1q gains in other types of neoplasia. However, in contrast to chromosome banding studies suggesting that the distal breakpoints in dup(1q) map proximal to the telomeric 1q44 band in ~75% of pediatric BCP ALL cases with gain of 1q[2], our SNP-A analyses revealed that the entire chromosome arm was gained in more than 80% of cases (Supplementary Figure 3). This clearly shows that chromosome banding analysis underestimates the sizes of the duplicated segments; thus, that method cannot be used reliably to delineate the minimally gained 1q region.

Analyses of carcinomas of the breast, liver, and ovary and Wilms’ tumors with 1q rearrangements have indicated that the near-centromeric 1q region is break-prone due to hypomethylation of sat II DNA[35, 41, 42]. However, we previously showed that sat II DNA on 1q is not hypomethylated in dup(1q)-positive HeH[21], strongly suggesting that other mechanisms underlie breaks in the near-centromeric region of 1q in pediatric BCP ALL, for example palindromic low-copy repeats in the vicinity of the breakpoint cluster region, as previously identified on 17p in idic(17)(p11)-positive malignancies[43–45]. The genomic sequence spanning the centromere and proximal 1q is highly repetitive. This, unfortunately, prevented exact breakpoint designations by the SNP-A and targeted deep sequencing analyses of the dup(1q) breakpoints and made it impossible to ascertain the possible mechanistic role of the genomic architectural features surrounding the 1q21.1 breakpoints. However, in the few cases with more distal breakpoints, we were able to identify small deletions adjacent to the breakpoints. It is presently unknown, and methodologically difficult to address, whether these were present before, possibly increasing the susceptibility for, the dup(1q) formation or occurred at the time of breakage or during the subsequent DNA repair steps.

It is generally assumed that gene-dosage effects affecting gene expression patterns are the pathogenetically important outcome of chromosomal gains. However, there are several examples of gains associated with gene mutations on the chromosomes involved, such as *KIT* mutations and trisomy 4 and internal tandem duplications of *KMT2A* and trisomy 11 in acute myeloid leukemia, *MET* mutations and trisomy 7 in hereditary papillary renal carcinomas, and *JAK2* mutations and trisomy 9 in polycythemia vera[46–49]. In order to investigate whether also gains of 1q are associated with mutations of genes on this chromosome arm, we analyzed the breakpoint regions and all exons on 1q using deep sequencing on a discovery cohort of seven dup(1q)-positive cases followed by TCA analyses of 111 cases w/wo 1q gains. Although non-synonymous somatic SNVs in the *BLZF1*, *FMN2*, *KCNT2*, *LCE1C*, *NES*, and *PARP1* genes were identified in the cases with dup(1q), the TCA analysis of the validation cohort revealed similar SNV frequencies of these genes in cases w/wo gain of 1q. Furthermore, none of the mutated genes was differentially expressed in the RNA-Seq analysis (Supplementary Tables 7 and 8). Thus, we conclude that these SNVs were passengers, without any association with dup(1q) as such.

We and others have previously identified altered methylation patterns in cases with genomic gains, such as hypomethylation of gene-poor regions on the trisomic/tetrasomic chromosomes in HeH, on chromosomes 7 and 14 in colon cancer with trisomies of these chromosomes, on chromosome 8 in constitutional trisomy 8 mosaicism, and on 12p in Pallister–Killian syndrome with gain of i(12p)[50–54]. We hence hypothesized that also gain of 1q might be associated with such methylation changes and ascertained the methylation status of cancer tissue-specific differentially methylated regions, promoters, CpG islands, shores, and shelves on 1q. However, HCA and PCA did not separate HeH and t(1;19) cases w/wo dup(1q) cases based on 1q status (Supplementary Figure 4) and less than 1% of the 36,259 CpG sites on 1q were differentially methylated. Thus, we found no evidence that dup(1q) is associated with any major methylation changes on 1q.

Only a handful of the differentially methylated CpGs were associated with gene expression changes in the cases analyzed by both BS-Seq and RNA-Seq. Interestingly, one of the genes hypomethylated and overexpressed in the dup(1q)-positive cases was *UCK2*, located in 1q24.1 and coding for uridine-cytidine kinase 2. This gene has previously been reported to be upregulated in a few dup(1q)-positive HeH cases[21], and overexpression of *UCK2* was recently shown to correlate with progression/poor prognosis in breast cancer[55]. However, *UCK2* was not among the close to 200 significantly overexpressed genes in the present series of HeH cases with 1q gain (Supplementary Table 7), making this gene an unlikely candidate to play an important pathogenetic or clinical role in HeH.

The RNA-Seq analysis revealed that gain of 1q had a profound impact on both the global gene expression profiles and on the expression of genes on this chromosome arm (Fig. 1; Supplementary Tables 7 and 8). It is noteworthy that the deregulated genes in the dup(1q)-positive HeH and t(1;19) cases were, with only a few exceptions, distinct. Hence, the gene-dosage effects of 1q gains in pediatric BCP ALL are context dependent, varying among genetic subgroups, possibly reflecting the presence of different primary, leukemia-initiating genetic changes and/or distinct differentiation stages of the cells of origin. Because a total of >100 genes on 1q were overexpressed in the HeH and t(1;19) cases with 1q gains, it is impossible to pinpoint a specific target gene. However, *CKS1B*, coding for the CDC28 protein kinase regulatory subunit 1B and known to be overexpressed in multiple myeloma[17, 18], was clearly not overexpressed. Thus, overexpression of *CSK1* is not the functionally important outcome of dup(1q) in HeH and t(1;19) cases, again emphasizing the variable gene-dosage effects of 1q gains in B-lineage malignancies. In our previous, smaller study of dup(1q)-positive HeH, we observed upregulation of the *DAP3* gene in 1q22 in a few cases[21]. This gene was also overexpressed in the present, larger cohort of HeH cases with 1q gain (Supplementary Table 7). DAP3 codes for the death-associated protein 3 that promotes apoptosis. This gene would hence not be expected to be upregulated in cancer; loss of function would be more likely. This notwithstanding, it is overexpressed in several types of malignancy, such as breast cancer, gastric cancer, thymoma, and glioblastoma[56–59]. Although overexpression of *DAP3* may be pathogenetically important, we deem it too simplistic to reduce the functional consequence of dup(1q) in HeH to gain and deregulation of a single gene. In contrast, overexpression of multiple genes, affecting different pathways and cellular processes, may be the essential outcome.

Interestingly, although the deregulated genes in the dup(1q)-positive HeH and t(1;19) cases were, with few exceptions, distinct (Supplementary Tables 7 and 8), GO analyses of the overexpressed genes on 1q revealed similar frequencies and types of molecular functions, biological processes, and protein classes of the upregulated genes in the two groups, with the majority encoding nucleic acid/protein binding factors or proteins with catalytic activity (Fig. 2 and Supplementary Figures 7 and 8). In fact, the frequent overexpression of genes coding for nucleic acid binding proteins, such as transcription factors, may be one reason for the quite pronounced impact of dup(1q) on the global gene expression patterns in both t(1;19) and HeH cases (Fig. 1).

In conclusion, dup(1q) in pediatric BCP ALL is not associated with SNVs or methylation changes on 1q. Instead, the pathogenetically important consequence of dup(1q) is a gene-dosage effect, and although the deregulated genes differ between dup(1q)-positive HeH and t(1;19) cases, the overexpressed genes on 1q are associated with similar molecular functions, biological processes, and protein classes irrespective of genetic subtype.

## Electronic supplementary material

Supplementary Table 1(DOCX 29 kb)

Supplementary Table 2(DOCX 43 kb)

Supplementary Table 3(DOCX 16 kb)

Supplementary Table 4(DOCX 19 kb)

Supplementary Table 5(DOCX 55 kb)

Supplementary Table 6(DOCX 96 kb)

Supplementary Table 7(DOCX 31 kb)

Supplementary Table 8(DOCX 44 kb)

Supplementary Figures(DOCX 8893 kb)
