# Supplementary material for: Mutation, methylation, and gene expression profiles in dup(1q)-positive pediatric B-cell precursor acute lymphoblastic leukemia
Source: Leukemia. 2018 Mar 12;32(10):2117–25. doi: 10.1038/s41375-018-0092-2 (PMC6170391; doi:10.1038/s41375-018-0092-2)
Supplement: Supplementary file 1 — Supplementary Table 1(DOCX 29 kb) [file 41375_2018_92_MOESM1_ESM.docx]

**Supplementary Table 1.** Basic clinical and cytogenetic data and types of analysis performed in 27 dup(1q)-positive BCP ALL cases

| *Case* | *Sex/* | *Sub-* | *Analyses performed* | | | | | *dup(1q)* | *Karyotype [includes also FISH and SNP-A findings]* |
| --- | --- | --- | --- | --- | --- | --- | --- | --- | --- |
| *No.* | *age* | *group* | *Targeted* | *TCA* | *Sanger* | *BS* | *RNA* |  |  |
|  | *(years)* |  | *1q seq/* |  | *seq* | *seq* | *seq* |  |  |
|  |  |  | *mutated* |  |  |  |  |  |  |
|  |  |  | *gene(s)* |  |  |  |  |  |  |
| 1 | M/3 | HeH | Yes/*LCE1C*, | Yes | Yes | Yes | Yes | dup(1)(q21.1q44) | 54,XY,+X,dup(1)(q21.1q44),+4,+6,dup(9)(p12p24),+10,+14,+17,+18, |
|  |  |  | *FMN2* |  |  |  |  |  | +dup(21)(q21.1q22.3)^a^ |
| 2 | M/7 | HeH | Yes/*PARP1* | Yes | Yes | Yes |  | dup(1)(q21.1q41) | 55,XY,+X,dup(1)(q21.1q41),+4,+6,+10,+17,+18,+18,+21,+21^a^ |
| 3 | M/3 | HeH | Yes/*KCNT2* | Yes | Yes | Yes | Yes | dup(1)(q21.1q31.1) | 55,XY,+X,der(1)dup(1)(q21.1q31.1)dup(1)(q31.2q41),+4,+6,+10,+14, |
|  |  |  |  |  |  |  |  | dup(1)(q31.2q41) | +17,+18,+21,+21^a^ |
| 4 | M/14 | t(1;19) | Yes/*NES* | Yes | Yes | Yes | Yes | dup(1)(q23.3q44) | 46,XY,der(19)t(1;19)(q23.3;p13.3)^a^ |
| 5 | M/3 | t(1;19) | Yes/*BLZF1* | Yes | Yes | Yes | Yes | dup(1)(q21.1q44) | 47,XY,+i(1)(q10),del(6)(q14.1q22.1),del(12)(p13.1),del(13)(q13.1), |
|  |  |  |  |  |  |  |  |  | dup(10)(p11.21p15.3),der(19)t(1;19)(q23.3;p13.3)^a^ |
| 6 | F/10 | t(12;21) | Yes/none | Yes |  | Yes |  | dup(1)(q21.1q44) | 46,del(X)(p21.1),dup(X)(q23q28),dup(1)(q21.1q44),del(3) |
|  |  |  |  |  |  |  |  |  | (p21.31p21.31),del(3)(q13.31q13.31),del(3)(q26.32q26.33),dup(4) |
|  |  |  |  |  |  |  |  |  | (q12q35.2),del(6)(q16.1),t(12;21)(p13;q22) |
| 7 | M/11 | B-other | Yes/none | Yes |  | Yes |  | dup(1)(q31.3q44) | 46,XY,del(1)(p21.1p21.2),del(1)(p31.1p31.3),del(1)(p31.3p31.3), |
|  |  |  |  |  |  |  |  |  | dup(1)(q31.3q44),del(3)(q12.1q13.33),del(3)(q13.33q22.1),del(3) |
|  |  |  |  |  |  |  |  |  | (q25.1q26.2),del(3)(q26.31q26.33),del(3)(q27.1q27.2),del(5) |
|  |  |  |  |  |  |  |  |  | (q32q33.1),del(7)(p14.3p14.3),del(7)(p15.3p15.3),del(7)(p21.1p21.1), |
|  |  |  |  |  |  |  |  |  | del(7)(q11.22q11.22),del(9)(p13.2p21.3),del(9)(p21.3p21.3)x2,del(13) |
|  |  |  |  |  |  |  |  |  | (q14.12q14.13),del(13)(q14.2q14.2)x2 |
| 8 | F/14 | B-other |  | Yes |  | Yes |  | dup(1)(q21.3q44) | 47,XX,del(8)(q13.1q13.1),del(8)(q21.3q21.3),dup(10)(q26.3q26.3), |
|  |  |  |  |  |  |  |  |  | dup(10)(q26.3q26.3),del(12)(p12.3p13.31),del(17)(q11.2q11.2),+22, |
|  |  |  |  |  |  |  |  |  | der(22)t(1;22)(q21.3;p11)x2 |
| 9 | M/1 | HeH |  | Yes |  | Yes |  | dup(1)(q21.1q44) | 63,XXY,+Y,-1,dup(1)(q21.1q44),-3,del(6)(q14),-7,-9,-13,-16,dup(16) |
|  |  |  |  |  |  |  |  |  | (q23.1q23.1),-19,-20,+dup(21)(q21.3q21.3)^a^ |
| 10 | M/11 | HeH |  | Yes |  | Yes |  | dup(1)(q21.3q44) | 58,XXY,+Y,-1,der(1)t(1;1)(p36;q21.3),-2,-3,+4,-5,-7,-9,-11,-12,del(12) |
|  |  |  |  |  |  |  |  |  | (p12.1p12.1),-13,-15,-16,-19,-20,+21,-22^a^ |
| 11 | F/16 | HeH |  | Yes |  | Yes | Yes | dup(1)(q21.2q44) | 61,XXX,-1,dup(1)(q21.2q44),-2,-3,-9,del(9)(p21.3p21.3)x2,-11,-12, |
|  |  |  |  |  |  |  |  |  | -13,+14,-15,-16,del(16)(q22.1q22.2),-19,dup(19)(q13.2q13.31),+21, |
|  |  |  |  |  |  |  |  |  | +21,-22^a^ |
| 12 | M/3 | HeH |  | Yes |  | Yes | Yes | dup(1)(q21.1q44) | 55,XY,+X,dup(1)(q21.1q44),+4,+5,+6,idic(7)(p12.1),+8,+10,del(13) |
|  |  |  |  |  |  |  |  |  | (q12.3q12.3),+14,+17,+21^a^ |
| 13 | M/3 | HeH |  | Yes |  | Yes | Yes | dup(1)(q21.1q42.13) | 52,XY,+X,dup(1)(q21.1q42.13),+6,+10,+11,+21,+21^a^ |
| 14 | M/5 | HeH |  | Yes |  | Yes | Yes | dup(1)(q21.1q44) | 57,XY,+X,dup(1)(q21.1q44),+4,+5,+6,dup(8)(q24.21q24.3),+9,+10, |
|  |  |  |  |  |  |  |  |  | dup(11)(p11.2p15.5),+14,+del(16)(p11.2),+18,+21,+21^a^ |
| 15 | F/8 | t(1;19) |  | Yes |  |  | Yes | dup(1)(q23.3q44) | 46,XX,dup(2)(p15p15),i(9)(q10),der(19)t(1;19)(q23.3;p13.3)^a^ |
| 16 | F/9 | t(1;19) |  | Yes |  |  | Yes | dup(1)(q23.3q44) | 46,XX,der(19)t(1;19)(q23.3;p13.3) |
| 17 | F/4 | t(1;19) |  | Yes |  |  |  | dup(1)(q21.1q44) | 47,XX,+idic(1)(p1?3),t(1;19)(q23;p13) |
| 18 | F/12 | HeH |  |  |  | Yes |  | dup(1)(q21.2q42.2) | 52,XX,+X,der(1)dup(1)(q21.2q42.2)del(1)(q42.2),+10,+14,dup(17) |
|  |  |  |  |  |  |  |  |  | (q11q25),+18,+21,+21/54,idem,+6,+18^a^ |
| 19 | M/6 | HeH |  |  |  |  | Yes | dup(1)(q21.1q44) | 59,XXY,add(1)(p11),-2,-3,-5,-7,-8,+10,-11,-12,-13,-15,-16,+18,-19, |
|  |  |  |  |  |  |  |  |  | -20,+21,-22^a^ |
| 20 | M/8 | HeH |  |  |  |  |  | dup(1)(q21.1q44) | 58,XXY,-1,dup(1)(q21.1q44),-2,-3,-7,-9,del(11)(q14q25),-12,-13,-15, |
|  |  |  |  |  |  |  |  |  | -16,i(16)(p10),-19,-20,+21,-22^a^ |
| 21 | M/4 | HeH |  |  |  |  |  | dup(1)(q21.1q44) | 54,XY,+X,dup(1)(q21.1q44),+6,+10,+14,+17,+18,del(19)(p13.3p13.3), |
|  |  |  |  |  |  |  |  |  | -20,+21,+21,+21,dup(22)(q11.23q11.23) |
| 22 | F/8 | HeH |  |  |  |  |  | dup(1)(q21.1q44) | 59,XX,-X,-1,dup(1)(q21.1q44),-2,dup(2)(q11q37),-3,-4,del(6) |
|  |  |  |  |  |  |  |  |  | (q12q22),-9,+10,-13,del(13)(q21),-15,-16,-17,-19,-20,+21,+21,-22^a^ |
| 23 | M/6 | HeH |  |  |  |  |  | dup(1)(q21.1q44) | 54,XY,+X,dup(1)(q21.1q44),+5,+6,del(6)(q14)x2,+8,del(11)(q14),+14, |
|  |  |  |  |  |  |  |  |  | dup(17)(q12q25),+18,+21,+21^a^ |
| 24 | F/3 | HeH |  |  |  |  |  | dup(1)(q21.1q41) | 56,XX,+X,dup(1)(q21.1q41),+4,+6,+8,+10,+14,+14,+18,+21,+21^a^ |
| 25 | M/4 | t(1;19) |  |  |  |  | Yes | dup(1)(q21.2q44) | 50,XY,dup(1)(q21.2q44),+4,+5,+8,+16,der(19)t(1;19)(q23.3;p13.3)^a^ |
| 26 | M/1 | t(1;19) |  |  |  |  | Yes | dup(1)(q23.3q44) | 46,XY,der(19)t(1;19)(q23.3;p13.3) |
| 27 | M/2 | t(1;19) |  |  |  |  | Yes | dup(1)(q23.3q44) | 46,XY,der(19)t(1;19)(q23.3;p13.3) |

Abbreviations: BCP ALL, B-cell precursor acute lymphoblastic leukemia; BS, bisulfite; F, female; FISH, fluorescence in situ hybridization; HeH, high hyperdiploidy (51-67 chromosomes); M, male; seq, sequencing; SNP-A, single nucleotide polymorphism array; TCA, Truseq custom amplicon. ^a^The karyotypes of these cases have previously been published.^1-5^

REFERENCES

1. Paulsson K, Jonson T, Øra I, Olofsson T, Panagopoulos I, Johansson B. Characterisation of genomic translocation breakpoints and identification of an alternative *TCF3*/*PBX1* fusion transcript in t(1;19)(q23;p13)-positive acute lymphoblastic leukaemias. *Br J Haematol* 2007; **138**: 196-201.
2. Paulsson K, Forestier E, Lilljebjörn H, Heldrup J, Behrendtz M, Young BD *et al*. Genetic landscape of high hyperdiploid childhood acute lymphoblastic leukemia. *Proc Natl Acad Sci USA* 2010; **107**: 21719-21724.
3. Andersen MK, Autio K, Barbany G, Borgström G, Cavelier L, Golovleva I *et al*. Paediatric B-cell precursor acute lymphoblastic leukaemia with t(1;19)(q23;p13): clinical and cytogenetic characteristics of 47 cases from the Nordic countries treated according to NOPHO protocols. *Br J Haematol* 2011; **155**: 235-243.
4. Olsson L, Albitar F, Castor A, Behrendtz M, Biloglav A, Paulsson K *et al*. Cooperative genetic changes in pediatric B-cell precursor acute lymphoblastic leukemia with deletions or mutations of *IKZF1*. *Genes Chromosomes Cancer* 2015; **54**: 315-325.
5. Paulsson K, Lilljebjörn H, Biloglav A, Olsson L, Rissler M, Castor A *et al*. The genomic landscape of high hyperdiploid childhood acute lymphoblastic leukemia. *Nat Genet* 2015; **47**: 672-676.
