# Supplementary material for: Mutation, methylation, and gene expression profiles in dup(1q)-positive pediatric B-cell precursor acute lymphoblastic leukemia
Source: Leukemia. 2018 Mar 12;32(10):2117–25. doi: 10.1038/s41375-018-0092-2 (PMC6170391; doi:10.1038/s41375-018-0092-2)
Supplement: Supplementary file 2 — Supplementary Table 2(DOCX 43 kb) [file 41375_2018_92_MOESM2_ESM.docx]

**Supplementary Table 2.** Basic clinical and cytogenetic data and types of analysis performed in 132 dup(1q)-negative BCP ALL cases

| *Case.* | *Sex/* | *Sub-* | *Analyses performed* | | | *Karyotype [includes also FISH and SNP-A findings in some cases]* |
| --- | --- | --- | --- | --- | --- | --- |
| *No.* | *age* | *group* | *TCA* | *BS* | *RNA* |  |
|  | *(years)* |  |  | *seq* | *seq* |  |
| 28 | F/3 | HeH | Yes | Yes | Yes | 58,XXX,-1,-2,-3,-5,-7,-8,-13,-15,-16,dup(17)(p11p13),-19,-20,+21,-22^a^ |
| 29 | F/5 | HeH | Yes | Yes | Yes | 53,XX,+4,+6,+10,+14,+18,+21,+21^a^ |
| 30 | M/1 | HeH | Yes | Yes | Yes | 55,XY,+X,+4,+6,+14,+17,+18,+21,+21,+22^a^ |
| 31 | M/1 | HeH | Yes | Yes | Yes | 55,XY,+X,+4,+6,+14,+15,+17,+18,+21,+dup(21)(q21q22)^a^ |
| 32 | M/2 | HeH | Yes | Yes | Yes | 56,XY,+X,+Y,+4,+6,+10,+14,+17,+18,+21,+21^a^ |
| 33 | F/3 | HeH | Yes | Yes | Yes | 55,XX,+X,+4,+6,+7,+10,+14,+17,+18,+21^a^ |
| 34 | M/2 | HeH | Yes |  | Yes | 56,XY,+X,+Y,+4,t(?5;9)(q?;p13-21),+6,+10,+14,+17,+18,+21,+22^a^ |
| 35 | F/4 | HeH | Yes |  | Yes | 52,XX,+X,+4,+8,+10,+18,+21^a^ |
| 36 | F/11 | HeH | Yes |  | Yes | 56,XX,+X,+4,+6,+10,+10,+14,+17,+18,+21,+21/56,idem,del(12)(p12p13),del(13)(q12q31)^a^ |
| 37 | F/3 | HeH | Yes |  | Yes | 57,XX,+X,+4,+6,+8,+8,+12,+14,+17,+18,+21,+21/58,idem,+10^a^ |
| 38 | M/7 | HeH | Yes |  | Yes | 60,XY,dup(X)(p11p22),-1,-2,-3,-7,-9,+del(10)(q23),-11,-12,-13,der(13;14)(q10;q10)c,-15,-16,+18,-19^a^ |
| 39 | F/3 | HeH | Yes |  | Yes | 56,XX,+X,+6,+8,+10,+11,+18,+18,+20,+21,+mar^a^ |
| 40 | M/2 | HeH | Yes |  | Yes | 52,XY,+4,+6,+17,+18,+21,+21^a^ |
| 41 | M/10 | HeH | Yes |  | Yes | 54,XY,+X,del(1)(p12p36),+6,i(9)(p10),+10,+14,+17,+18,+21,+21^a^ |
| 42 | M/5 | HeH | Yes |  |  | 55,XY,+del(X)(q13),+3,+6,+10,+10,+14,+18,+21,+21^a^ |
| 43 | M/3 | HeH | Yes |  |  | 56,XY,+X,+4,+6,+8,+10,+14,+17,+18,+21,+21^a^ |
| 44 | M/4 | HeH | Yes |  |  | 62,XXY,-1,-2,-7,-9,+10,-11,-12,+14,-15,-16,+18,-19,-20,+21,-22/62,idem,del(17)(p11)^a^ |
| 45 | F/2 | HeH | Yes |  |  | 52,XX,+X,+4,+6,+17,+18,+21^a^ |
| 46 | F/4 | HeH |  | Yes | Yes | 54,XX,+X,+4,+6,+14,+17,+dup(18)(q22q23),+21,+21^a^ |
| 47 | M/13 | HeH |  | Yes | Yes | 55,XY,+X,+4,+6,add(8)(q?22),+10,+14,+der(17)t(17;19)(q?;?)del(17)(p11p13),+18,der(19)t(17;19),+21,+21^a^ |
| 48 | M/4 | HeH |  | Yes | Yes | 56,XY,+X,+3,+4,+6,+8,+10,+14,+16,+18,+21/56,idem,i(7)(q10)^a^ |
| 49 | M/6 | HeH |  | Yes |  | 54,XY,+X,+4,+8,+14,+17,+18,+21,+21^a^ |
| 50 | M/3 | HeH |  |  | Yes | 53,XY,+X,+6,+10,+17,+18,+21,+21 |
| 51 | F/6 | HeH |  |  | Yes | 53,XX,+X,+4,+6,+14,+17,+21,+21 |
| 52 | F/8 | HeH |  |  | Yes | 59,XXX,-1,-2,-3,-5,-7,-8,+10,-11,-12,-13,-16,-17,idic(17)(p11),-19,+21^a^ |
| 53 | F/15 | HeH |  |  | Yes | 57,XX,+X,+X,+4,+6,der(8)t(8;14)(p11;q12),+10,+14,+14,+17,+18,+21,+21^a^ |
| 54 | M/17 | HeH |  |  | Yes | 53-54,XY,+X,+4,+12,+21,+21,+mar,inc |
| 55 | F/2 | HeH |  |  | Yes | 53,XX,+X,+8,+14,+15,+17,+21,+21^a^ |
| 56 | M/5 | HeH |  |  | Yes | 55,XY,+X,+6,+9,+14,+14,+17,+18,+21,+21/55,idem,dup(4)(p15p16),dup(10)(q11q26)^a^ |
| 57 | M/16 | HeH |  |  | Yes | ??,XY,+X,+6,+8,+8,+10,+14,+14,+18,+18,+21,+21,inc^a^ |
| 58 | F/2 | HeH |  |  | Yes | 56,XX,+X,+4,+6,+8,+10,+14,+17,+18,+21,+21^a^ |
| 59 | M/7 | HeH |  |  | Yes | 56,XY,+X,dup(3)(q21q29),+4,+6,+10,+14,+14,+del(17)(p11),+18,+18,+21^a^ |
| 60 | F/5 | HeH |  |  | Yes | 55,XX,+X,+2,+4,+6,+8,+14,+21,+21,+21^a^ |
| 61 | F/7 | HeH |  |  | Yes | 59,XXX,-1,-2,-3,-7,-9,-11,-12,-13,+14,-15,-16,+18,-19,-20,+21,-22^a^ |
| 62 | F/1 | HeH |  |  | Yes | 54,XX,+X,+4,+6,+8,+14,+17,+18,+21^a^ |
| 63 | M/2 | HeH |  |  | Yes | 52,XY,+X,+6,+13,+15,+17,+21 |
| 64 | M/2 | HeH |  |  | Yes | 54,XY,+X,+4,+6,+10,+14,+17,+18,+21/55,idem,+21^a^ |
| 65 | M/6 | HeH |  |  | Yes | 59-60,XXY,-1,-2,-3,-7,-9,add(11)(q23),-13,-15,-16,-18,-19,-20,+21,inc |
| 66 | M/6 | HeH |  |  | Yes | 55,XY,+X,+4,i(7)(q10),+8,+9,+10,+14,+18,+21,+21^a^ |
| 67 | M/3 | HeH |  |  | Yes | 53,XY,+X,+4,+6,dup(7)(p15p22),+14,+17,+18,+add(21)(q22)^a^ |
| 68 | M/3 | HeH |  |  | Yes | 57,XY,+X,+Y,+4,+5,+6,+10,+14,+17,+21,+21,+21^a^ |
| 69 | M/2 | HeH |  |  | Yes | 53,XY,+X,+6,+14,+17,+18,+21,+21^a^ |
| 70 | M/9 | HeH |  |  | Yes | 58,XXY,-1,-2,?del(2)(q11),-3,del(6)(q21),-7,-9,-10,-11,?add(11)(p15),-12,-13,del(14)(q23),-15,-16,-18,+21 |
| 71 | F/4 | HeH |  |  | Yes | 51,XX,+X,+6,+14,+17,+18/53,idem,+21,+21^a^ |
| 72 | M/1 | HeH |  |  | Yes | 63,XXY,+Y,-1,-3,-7,-13,-15,-16,-19,-20,+21 |
| 73 | M/1 | HeH |  |  | Yes | 63,XXY,-1,-2,-3,-8,-9,-13,+14,-15,-19,-20,+21,+21^a^ |
| 74 | M/3 | HeH |  |  | Yes | 55,X,+X,-Y,+4,+6,+?add(7)(p11),-9,+11,+12,+14,+17,+18,+21,+21 |
| 75 | M/3 | HeH |  |  | Yes | ??,XY,+X,+4,+6,+10,+14,+17,+18,+21,+21,+21,inc |
| 76 | F/4 | HeH |  |  | Yes | 54,XX,+X,+4,+6,+10,+17,+18,+21,+21^a^ |
| 77 | F/2 | HeH |  |  | Yes | 55,XX,+X,+4,+6,+10,+14,+17,+18,+21,+21 |
| 78 | F/2 | HeH |  |  | Yes | 54,XX,+X,+6,+10,+14,+17,+18,+21,+21^a^ |
| 79 | F/2 | t(1;19) | Yes | Yes | Yes | 46,XX,t(1;19)(q23;p13) |
| 80 | F/3 | t(1;19) | Yes |  | Yes | 46,XX,t(1;19)(q23;p13)/47,idem,+6 |
| 81 | F/1 | t(1;19) |  | Yes | Yes | 46,XX,t(1;19)(q23;p13) |
| 82 | F/9 | t(1;19) |  |  | Yes | 46,XX,t(1;19)(q23;p13) |
| 83 | F/3 | t(1;19) |  |  | Yes | 46,XX,t(1;19)(q23;p13),inc^a^ |
| 84 | M/14 | t(1;19) |  |  | Yes | 47,XY,t(1;19)(q23;p13),+5 |
| 85 | M/4 | t(12;21) | Yes | Yes |  | 46,XY,del(12)(p13p13),t(12;21)(p13;q22) |
| 86 | M/4 | t(12;21) | Yes |  |  | 46,XY,del(2)(p11),add(3)(p11),add(6)(q21),add(12)(p13),der(12)t(12;21)(p13;q22),ider(21)(q10)t(12;21),+2-3mar,inc^a^ |
| 87 | F/7 | t(12;21) | Yes |  |  | 46,XX,-5,add(6)(q?15),add(7)(q31),add(12)(p11),t(12;21)(p13;q22),-13,-13,+21,?der(21;21)(q10;q10),+2-3mar |
| 88 | M/5 | t(12;21) | Yes |  |  | 46,XY,t(12;21)(p13;q22)/47,idem,+16/47,idem,der(6)t(X;6)(?;q1?),+16^a^ |
| 89 | F/3 | t(12;21) | Yes |  |  | 46,XX,t(12;21)(p13;q22) |
| 90 | M/3 | t(12;21) | Yes |  |  | ??,X?,t(12;21)(p13;q22),+der(?)t(?;21)(?;q?)/??,X?,t(12;21),+21,+der(?)t(?;12)t(12;21),inc |
| 91 | F/10 | t(12;21) | Yes |  |  | ??,X,-X,+4,+4,+6,+6,+8,+8,+10,+10,t(12;21)(p13;q22)x1-2,+14,+14,+17,+17,+18,+18,+21,+21,+21,inc |
| 92 | F/4 | t(12;21) | Yes |  |  | 46,XX,del(12)(p13p13),t(12;21)(p13;q22) |
| 93 | F/3 | t(12;21) | Yes |  |  | ??,X?,del(12)(p13p13),t(12;21)(p13;q22),inc |
| 94 | F/4 | t(12;21) | Yes |  |  | ??,X?,t(12;21)(p13;q22),+der(21)t(12;21),inc |
| 95 | F/5 | t(12;21) | Yes |  |  | 45,XX,add(6)(q15),del(12)(p11),t(12;21)(p13;q22),-13,add(15)(q22)^a^ |
| 96 | F/3 | t(12;21) | Yes |  |  | 46,XX,t(12;21)(p13;q22) |
| 97 | F/4 | t(12;21) | Yes |  |  | 46,XX,t(12;21)(p13;q22) |
| 98 | M/3 | t(12;21) | Yes |  |  | ??,X?,t(12;21)(p13;q22),+der(21)t(12;21),inc |
| 99 | M/5 | t(12;21) | Yes |  |  | 46,XY,dup(5)(q34q35),der(6)t(X;6)(q21;q14),del(11)(q14q25),t(12;21)(p13;q22)^a^ |
| 100 | M/6 | t(12;21) | Yes |  |  | 46,XY,t(12;21)(p13;q22) |
| 101 | F/6 | t(12;21) | Yes |  |  | ??,X?,t(12;21)(p13;q22),inc^a^ |
| 102 | M/5 | t(12;21) | Yes |  |  | 46,dup(X)(q25q28),Y,t(12;21)(p13;q22),add(16)(q21)^a^ |
| 103 | M/3 | t(12;21) | Yes |  |  | 46,XY,del(12)(p13p13),t(12;21)(p13;q22)/47,idem,+der(21)t(12;21) |
| 104 | M/7 | t(12;21) | Yes |  |  | 46,XY,del(6)(q14q22),del(6)(q24q27),dup(8)(q13q24),der(12)t(12;21)(p13;q22),del(13)(q13q31),ider(21)(q10)t(12;21)^a^ |
| 105 | F/4 | t(12;21) | Yes |  |  | 46,XX,del(12)(p12p13),t(12;21)(p13;q22)^a^ |
| 106 | F/3 | t(12;21) | Yes |  |  | 46-47,XX,der(2)t(2;5)(p13;q13),del(4)(q11),del(5)(q13),der(6)t(2;6)(p13;p22),-9,del(12)(p11),der(12)t(4;12)(q11;p12), |
|  |  |  |  |  |  | ?add(13)(q?),-15,+21,der(21)t(12;21)(p13;q22)x2,+der(?)t(?;6)(?;p?),+mar/46-47,idem,del(3)(q27),-?add(13), |
|  |  |  |  |  |  | +der(13)?t(3;13)(q27;q?)^a^ |
| 107 | M/8 | t(12;21) | Yes |  |  | 46,XY,der(12)t(12;21)(p13;q22),ider(21)(q10)t(12;21) |
| 108 | M/0 | t(12;21) | Yes |  |  | 52,XY,+X,+4,der(6)t(6;12)(p1?2;q15),add(8)(p?21),+9,+10,del(12)(q15),der(12)t(6;12)(p1?2;p13)ins(12;21)(p13;q22), |
|  |  |  |  |  |  | +der(21)t(12;21)(p13;q22)x2 |
| 109 | M/4 | t(12;21) | Yes |  |  | ??,X?,t(12;21)(p13;q22),inc^a^ |
| 110 | M/2 | t(12;21) | Yes |  |  | 47,XY,add(12)(p11),t(12;21)(p13;q22),+21 |
| 111 | M/3 | t(12;21) | Yes |  |  | 46,XY,?add(7)(p21),del(12)(p13p13),t(12;21)(p13;q22),add(15)(q21),add(22)(q13) |
| 112 | M/6 | t(12;21) | Yes |  |  | 46,XY,t(12;21)(p13;q22)/46,idem,add(12)(p13) |
| 113 | F/6 | t(12;21) | Yes |  |  | 46,XX,del(6)(q21),t(12;21)(p13;q22)/47,XX,t(12;21),+21/47,XX,t(12;21),+der(21)t(12;21)^a^ |
| 114 | F/3 | t(12;21) | Yes |  |  | 46,XX,del(12)(p13p13),der(21)t(12;21)(p13;q22),inc^a^ |
| 115 | F/2 | t(12;21) | Yes |  |  | 46,XX,del(3)(p23p26),del(12)(p13p13),t(12;21)(p13;q22)/46,XX,der(3)del(3)(p23)del(3)(q27),del(12),t(12;21) |
| 116 | F/3 | t(12;21) | Yes |  |  | 49,XX,+X,t(12;21)(p13;q22),+18,+21^a^ |
| 117 | F/2 | B-other | Yes | Yes |  | 46,XX |
| 118 | F/11 | B-other | Yes |  |  | 46,X,-?X,-13,-17,+3mar^a^ |
| 119 | M/12 | B-other | Yes |  |  | 46,XY,inv(9)(p11q12)c |
| 120 | M/2 | B-other | Yes |  |  | 46,XY |
| 121 | F/1 | B-other | Yes |  |  | 46,XX |
| 122 | F/2 | B-other | Yes |  |  | 46,XX |
| 123 | M/0 | B-other | Yes |  |  | 46,XY |
| 124 | F/0 | B-other | Yes |  |  | 46,XX |
| 125 | M/3 | B-other | Yes |  |  | 46,XY |
| 126 | F/1 | B-other | Yes |  |  | 45,XX,t(7;22)(p15;q11),dic(9;20)(p13;q11)^a^ |
| 127 | F/5 | B-other | Yes |  |  | 47,XX,+X,t(1;12)(q21;p13) |
| 128 | F/4 | B-other | Yes |  |  | 46,XX |
| 129 | F/6 | B-other | Yes |  |  | Failure |
| 130 | M/3 | B-other | Yes |  |  | 47,XY,der(3)t(3;7)(q2?;p?),der(7)t(3;7)(?;p?),+21c,inc^a^ |
| 131 | M/5 | B-other | Yes |  |  | 49,XY,+X,+21c,+21^a^ |
| 132 | M/14 | B-other | Yes |  |  | 46,XY,-12,?t(14;15)(q21;q25),+r |
| 133 | M/6 | B-other | Yes |  |  | 46,XY^a^ |
| 134 | F/8 | B-other | Yes |  |  | 48,XX,+X,del(9)(p21p21),+21c^a^ |
| 135 | M/14 | B-other | Yes |  |  | 46,XY |
| 136 | M/4 | B-other | Yes |  |  | 49,XY,+21,+21,+mar^a^ |
| 137 | M/5 | B-other | Yes |  |  | 49,XY,+?X,?add(10)(q22),+17,+21 |
| 138 | M/0 | B-other | Yes |  |  | 46,XY,add(7)(q3?),inc |
| 139 | F/7 | B-other | Yes |  |  | 46,XX |
| 140 | M/17 | B-other | Yes |  |  | 45,XY,t(1;8)(q25;p23),-9,der(16)t(9;16)(q13;p13)/46,idem,+mar |
| 141 | M/11 | B-other | Yes |  |  | 47,XY,+7 |
| 142 | F/3 | B-other | Yes |  |  | 46,XX |
| 143 | M/10 | B-other | Yes |  |  | 46,XY |
| 144 | F/2 | B-other | Yes |  |  | 48,XX,+X,+21c |
| 145 | F/2 | B-other | Yes |  |  | 45,XX,del(2)(p13),-9,-18,-20,+2-3mar,inc |
| 146 | F/2 | B-other | Yes |  |  | 45,XX,dic(9;20)(p13;q11) |
| 147 | F/1 | B-other |  | Yes |  | 46,XX,+2mar,inc^a^ |
| 148 | M/13 | *KMT2A* | Yes |  |  | 46,XY,t(4;11)(q21;q23)^a^ |
| 149 | F/0 | *KMT2A* | Yes |  |  | 46,XX,t(11;19)(q23;p13) |
| 150 | M/2 | *KMT2A* | Yes |  |  | 47-48,XY,t(9;11)(p21;q23),+1-2mar^a^ |
| 151 | M/1 | *KMT2A* | Yes |  |  | 46,XY,t(4;11)(q21;q23) |
| 152 | F/0 | *KMT2A* | Yes |  |  | 46,XX,t(4;11)(q21;q23)^a^ |
| 153 | F/0 | *KMT2A* | Yes |  |  | 46,XX,t(11;19)(q23;p13)^a^ |
| 154 | F/0 | *KMT2A* | Yes |  |  | ??,X?,der(11)(q23),inc |
| 155 | M/3 | t(9;22) | Yes |  |  | ??,X?,+9,t(9;22)(q34;q11),+18,+der(22)t(9;22),inc^a^ |
| 156 | M/6 | t(9;22) | Yes |  |  | 46,XY,t(9;22)(q34;q11)/47,idem,+der(22)t(9;22)/47,idem,+4 |
| 157 | M/2 | t(9;22) | Yes |  |  | 46,XY,t(9;22)(q34;q11)/45,idem,dic(12;20)(p11;q11)^a^ |
| 158 | M/4 | t(9;22) | Yes |  |  | 50,XY,+4,+5,t(8;14)(q11;q32),t(9;22)(q34;q11),+der(14)t(8;14),der(16)t(14;16)(q11;p13)t(8;14),+21^a^ |
| 159 | M/5 | HoL | Yes |  |  | 38,XY,-2,-3,-4,-5,+6,-7,-8,-9,+10,-12,-15,-17/68,idemx2,-Y,+5,+5,-6,-6,+8,+8,-10,-10,-13,-14,-16,-16,-18,-20,-22 |

Abbreviations: BCP ALL, B-cell precursor acute lymphoblastic leukemia; BS, bisulfite; F, female; FISH, fluorescence in situ hybridization; HeH, high hyperdiploidy (51-67 chromosomes); HoL, low hypodiploidy (30-39 chromosomes); M, male; seq, sequencing; SNP-A; single nucleotide polymorphism array; TCA, Truseq custom amplicon. ^a^The karyotypes of these cases have previously been published.^1-12^

REFERENCES

1. Andreasson P, Höglund M, Bekassy AN, Garwicz S, Heldrup J, Mitelman F *et al*. Cytogenetic and FISH studies of a single center consecutive series of 152 childhood acute lymphoblastic leukemias. *Eur J Haematol* 2000; **65**: 40-51.
2. Davidsson J, Paulsson K, Johansson B. Searching for cryptic chromosomal aberrations in high hyperdiploid childhood acute lymphoblastic leukaemias. *Eur J Haematol* 2006; **76**: 449-450.
3. Lilljebjörn H, Heidenblad M, Nilsson B, Lassen C, Horvat A, Heldrup J *et al*. Combined high-resolution array-based comparative genomic hybridization and expression profiling of *ETV6*/*RUNX1*-positive acute lymphoblastic leukemias reveal a high incidence of cryptic Xq duplications and identify several putative target genes within the commonly gained region. *Leukemia* 2007; **21**: 2137-2144.
4. Paulsson K, Jonson T, Øra I, Olofsson T, Panagopoulos I, Johansson B. Characterisation of genomic translocation breakpoints and identification of an alternative *TCF3*/*PBX1* fusion transcript in t(1;19)(q23;p13)-positive acute lymphoblastic leukaemias. *Br J Haematol* 2007; **138**: 196-201.
5. Forestier E, Gauffin F, Andersen MK, Autio K, Borgström G, Golovleva I *et al*. Clinical and cytogenetic features of pediatric dic(9;20)(p13.2;q11.2)-positive B-cell precursor acute lymphoblastic leukemias: A Nordic series of 24 cases and review of the literature. *Genes Chromosomes Cancer* 2008; **47**: 149-158.
6. Davidsson J, Lilljebjörn H, Andersson A, Veerla S, Heldrup J, Behrendtz M *et al*. The DNA methylome of pediatric acute lymphoblastic leukemia. *Hum Mol Genet* 2009; **18**: 4054-4065.
7. Lundin C, Heldrup J, Ahlgren T, Olofsson T, Johansson B. B-cell precursor t(8;14)(q11;q32)-positive acute lymphoblastic leukemia in children is strongly associated with Down syndrome or with a concomitant Philadelphia chromosome. *Eur J Haematol* 2009; **82**: 46-53.
8. Paulsson K, Forestier E, Lilljebjörn H, Heldrup J, Behrendtz M, Young BD *et al*. Genetic landscape of high hyperdiploid childhood acute lymphoblastic leukemia. *Proc Natl Acad Sci USA* 2010; **107**: 21719-21724.
9. Olsson L, Castor A, Behrendtz M, Biloglav A, Forestier E, Paulsson K *et al*. Deletions of *IKZF1* and *SPRED1* are associated with poor prognosis in a population-based series of pediatric B-cell precursor acute lymphoblastic leukemia diagnosed between 1992 and 2011. *Leukemia* 2014; **28**: 302-310.
10. Lundin C, Forestier E, Klarskov Andersen M, Autio K, Barbany G, Cavelier L *et al*. Clinical and genetic features of pediatric acute lymphoblastic leukemia in Down syndrome in the Nordic countries. *J Hematol Oncol* 2014; **7**: 32.
11. Olsson L, Albitar F, Castor A, Behrendtz M, Biloglav A, Paulsson K *et al*. Cooperative genetic changes in pediatric B-cell precursor acute lymphoblastic leukemia with deletions or mutations of *IKZF1*. *Genes Chromosomes Cancer* 2015; **54**: 315-325.
12. Paulsson K, Lilljebjörn H, Biloglav A, Olsson L, Rissler M, Castor A *et al*. The genomic landscape of high hyperdiploid childhood acute lymphoblastic leukemia. *Nat Genet* 2015; **47**: 672-676.
