# Supplementary material for: Mutation, methylation, and gene expression profiles in dup(1q)-positive pediatric B-cell precursor acute lymphoblastic leukemia
Source: Leukemia. 2018 Mar 12;32(10):2117–25. doi: 10.1038/s41375-018-0092-2 (PMC6170391; doi:10.1038/s41375-018-0092-2)
Supplement: Supplementary file 3 — Supplementary Table 3(DOCX 16 kb) [file 41375_2018_92_MOESM3_ESM.docx]

**Supplementary Table 3.** Primers used for Sanger sequencing

| *Gene oligo* | *Nt position^a^* | *Nt change* | *Oligo sequence (5' to 3')* |
| --- | --- | --- | --- |
| *BLZF1_F* | 169345925 | G>A | CGTTACTTCATCCCCAATCC |
| *BLZF1_R* | 169345925 | G>A | TCTCCTTTATGATGTCCCAGAGA |
| *FMN2_F* | 240497193 | A>T | GGCCAAGACAATCCAAAATG |
| *FMN2_R* | 240497193 | A>T | GCACTGTTCTTTTCCAGCATC |
| *KCNT2_1F* | 196227521 | G>A | CAGTCGCTGCTGGGTTATTT |
| *KCNT2_1R* | 196227521 | G>A | TGCTCTTTCTGTGAAAACTCTCC |
| *KCNT2_2F* | 196205189 | C>T | GCACACAACACATGAAATGC |
| *KCNT2_2R* | 196205189 | C>T | CACAATCATGCTGTGGACAA |
| *LCE1C_F* | 152777863 | G>T | CACTGCAGCAGGAAGAGACA |
| *LCE1C_R* | 152777863 | G>T | TGCCTAGGTCTGACTTGCTGT |
| *NES_1F* | 156641684 | G>A | GCCTCTACGCTCTCTTCTTTGA |
| *NES_1R* | 156641684 | G>A | ATCAGGAACCCCTGAGGTCT |
| *NES_2F* | 156640678 | G>A | ACCCTCTTTGCCTCCAAACT |
| *NES_2R* | 156640678 | G>A | GAAGGGCAATCACAACAGGT |
| *NES_3F* | 156641537 | C>T | TCACTTCCACAGACTCCAGTG |
| *NES_3R* | 156641537 | C>T | AAGACCCCCAGAAAAAGTGG |
| *PARP1_1F* | 226564978 | C>T | TCCAGGGGGTAGAACTTTTTG |
| *PARP1_1R* | 226564978 | C>T | CCATCCTTTTGGGGTGTGTA |
| *PARP1_2F* | 226570767 | G>A | TCCACACCACCAGCAAGTAG |
| *PARP1_2R* | 226570767 | G>A | CTTTGGTCTATGGGGTGGTT |

Abbreviations: F, forward; Nt, nucleotide; R; reverse. ^a^Chromosome 1 positions according to the

GRCh37 genome build.
