# Supplementary material for: Mutation, methylation, and gene expression profiles in dup(1q)-positive pediatric B-cell precursor acute lymphoblastic leukemia
Source: Leukemia. 2018 Mar 12;32(10):2117–25. doi: 10.1038/s41375-018-0092-2 (PMC6170391; doi:10.1038/s41375-018-0092-2)
Supplement: Supplementary file 4 — Supplementary Table 4(DOCX 19 kb) [file 41375_2018_92_MOESM4_ESM.docx]

**Supplementary Table 4.** SNP-A data on the dup(1q)-positive pediatric BCP ALL cases

| *Case* | *dup(1q)* | *Subgroup* | *Start^a^* | *Stop^a^* | *Size (Mb)* |
| --- | --- | --- | --- | --- | --- |
| *No.* |  |  |  |  |  |
| 1 | dup(1)(q21.1q44) | HeH | 146767908 | 249250621 | 102.5 |
| 2 | dup(1)(q21.1q41) | HeH | 145444556 | 229805213 | 84.4 |
| 3 | dup(1)(q21.1q31.1) | HeH | 146978395 | 186315707 | 39.3 |
|  | dup(1)(q31.2q41) |  | 190785818 | 215211418 | 24.4 |
| 4 | dup(1)(q23.3q44) | t(1;19) | 164793178 | 249250621 | 84.5 |
| 5 | dup(1)(q21.1q44) | t(1;19) | 145570627 | 249250621 | 103.7 |
| 6 | dup(1)(q21.1q44) | t(12;21) | 146559165 | 249250621 | 102.7 |
| 7 | dup(1)(q31.3q44) | B-other | 197843204 | 249250621 | 51.4 |
| 8 | dup(1)(q21.3q44) | B-other | 155402347 | 249250621 | 93.8 |
| 9 | dup(1)(q21.1q44) | HeH | 145657708 | 249250621 | 103.6 |
| 10 | dup(1)(q21.3q44) | HeH | 151372035 | 249250621 | 97.9 |
| 11 | dup(1)(q21.2q44) | HeH | 149909495 | 249250621 | 99.3 |
| 12 | dup(1)(q21.1q44) | HeH | 145558824 | 249250621 | 103.7 |
| 13 | dup(1)(q21.1q42.13) | HeH | 145399229 | 227221715 | 81.8 |
| 14 | dup(1)(q21.1q44) | HeH | 146997245 | 249250621 | 102.3 |
| 15 | dup(1)(q23.3q44) | t(1;19) | 164656976 | 249250621 | 84.6 |
| 16 | dup(1)(q23.3q44) | t(1;19) | 164829035 | 249250621 | 84.4 |
| 17 | dup(1)(q21.1q44) | t(1;19) | 145385579 | 249250621 | 103.9 |
| 18 | dup(1)(q21.2q42.2) | HeH | 149881900 | 231730121 | 81.8 |
| 19 | dup(1)(q21.1q44) | HeH | 145388137 | 249250621 | 103.9 |
| 20 | dup(1)(q21.1q44) | HeH | 145586780 | 249250621 | 103.7 |
| 21 | dup(1)(q21.1q44) | HeH | 145395440 | 249250621 | 103.9 |
| 22 | dup(1)(q21.1q44) | HeH | 146523045 | 249250621 | 102.7 |
| 23 | dup(1)(q21.1q44) | HeH | 146559165 | 249250621 | 102.7 |
| 24 | dup(1)(q21.1q41) | HeH | 146571244 | 220264913 | 73.7 |
| 25 | dup(1)(q21.2q44) | t(1;19) | 149763202 | 249250621 | 99.5 |
| 26 | dup(1)(q23.3q44) | t(1;19) | 164765566 | 249250621 | 84.5 |
| 27 | dup(1)(q23.3q44) | t(1;19) | 164695090 | 249250621 | 84.6 |

Abbreviations: BCP ALL, B-cell precursor acute lymphoblastic leukemia; HeH, high hyperdiploidy (51-67 chromosomes); SNP-A, single nucleotide polymorphism array. ^a^Chromosome 1 positions according to the GRCh37 genome build.
