# Supplementary material for: Mutation, methylation, and gene expression profiles in dup(1q)-positive pediatric B-cell precursor acute lymphoblastic leukemia
Source: Leukemia. 2018 Mar 12;32(10):2117–25. doi: 10.1038/s41375-018-0092-2 (PMC6170391; doi:10.1038/s41375-018-0092-2)
Supplement: Supplementary file 5 — Supplementary Table 5(DOCX 55 kb) [file 41375_2018_92_MOESM5_ESM.docx]

**Supplementary Table 5.** SNVs found by sequencing of 1q in seven dup(1q)-positive BCP ALL cases

| *Case* | *Position^a^* | *Nt change* | *Gene/* | *Region* | *Read* | *dbSNP-id^b^* |
| --- | --- | --- | --- | --- | --- | --- |
| *No.* |  |  | *ncRNA* | *(type)* | *count* |  |
|  |  |  |  |  | *(var:ref)* |  |
| 1 | 152777863 | G>T^c^ | *LCE1C* | Exonic (n-syn SNV) | 28:44 | Novel |
| 4 | 156641684 | G>A^c^ | *NES* | Exonic (n-syn SNV) | 17:51 | Novel |
| 5 | 169345925 | G>A^c^ | *BLZF1* | Exonic (n-syn SNV) | 10:48 | Novel |
| 3 | 196227521 | G>A^c^ | *KCNT2* | Exonic (n-syn SNV) | 31:24 | rs190445712 |
| 2 | 226564978 | C>T^c^ | *PARP1* | Exonic (n-syn SNV) | 46:24 | Novel |
| 1 | 240497193 | A>T^c^ | *FMN2* | Exonic (n-syn SNV) | 17:25 | Novel |
| 6 | 248737734 | G>A | *OR2T34* | Exonic (n-syn SNV) | 4:42 | rs139616012 |
| 3 | 152286042 | G>A | *FLG* | Exonic (syn SNV) | 11:173 | Novel |
| 5 | 152770351 | T>A | *LCE1D* | Exonic (syn SNV) | 5:48 | Novel |
| 5 | 152777838 | A>T | *LCE1C* | Exonic (syn SNV) | 4:88 | Novel |
| 7 | 144620842 | G>A | *NBPF8, NBPF9* | Intronic | 6:50 | Novel |
| 6 | 144674455 | G>T | *NBPF8, NBPF9* | Intronic | 4:24 | Novel |
| 4 | 144860862 | A>G | *PDE4DIP* | Intronic | 5:20 | rs111883420 |
| 3 | 144906007 | G>A | *PDE4DIP* | Intronic | 7:25 | Novel |
| 1 | 144967030 | C>G | *PDE4DIP* | Intronic | 4:57 | rs151251938 |
| 4 | 144985742 | G>A | *PDE4DIP* | Intronic | 3:29 | rs2590124 |
| 6 | 144994110 | G>A | *PDE4DIP* | Intronic | 5:23 | rs11809608 |
| 1 | 145220544 | C>T | *NOTCH2NL* | Intronic | 5:41 | rs61790263 |
| 4 | 145230357 | A>C | *NOTCH2NL* | Intronic | 5:21 | rs75002292 |
| 7 | 145264044 | A>G | *NOTCH2NL* | Intronic | 4:59 | rs28564429 |
| 5 | 145319407 | G>T | *NBPF10* | Intronic | 9:78 | Novel |
| 4 | 145355318 | A>C | *NBPF10* | Intronic | 3:7 | rs201514378 |
| 3 | 146113609 | A>G | *NBPF10* | Intronic | 3:17 | rs2746870 |
| 5 | 146401081 | G>A | *NBPF10* | Intronic | 6:45 | Novel |
| 5 | 146441125 | G>A | *NBPF10* | Intronic | 6:98 | Novel |
| 5 | 146441129 | A>G | *NBPF10* | Intronic | 13:91 | Novel |
| 5 | 146441144 | A>G | *NBPF10* | Intronic | 32:72 | Novel |
| 5 | 146441149 | C>G | *NBPF10* | Intronic | 5:100 | Novel |
| 5 | 148009757 | G>A | *NBPF14* | Intronic | 3:21 | rs61801154 |
| 2 | 148012892 | C>G | *NBPF14* | Intronic | 53:503 | Novel |
| 1 | 148559920 | G>A | *NBPF15* | Intronic | 5:85 | rs28360928 |
| 2 | 150360043 | C>T | *RPRD2* | Intronic | 9:17 | rs2487761 |
| 1 | 150389701 | C>G | *RPRD2* | Intronic | 46:25 | Novel |
| 4 | 151679963 | C>T | *CELF3* | Intronic | 6:5 | rs2280473 |
| 7 | 155369922 | A>T | *ASH1L* | Intronic | 13:21 | Novel |
| 6 | 164705020 | C>T | *PBX1* | Intronic | 20:155 | Novel |
| 5 | 164752099 | A>G | *PBX1* | Intronic | 19:105 | Novel |
| 2 | 164794210 | C>T | *PBX1* | Intronic | 161:89 | Novel |
| 6 | 168513129 | C>T | *XCL2* | Intronic | 4:48 | rs3820405 |
| 5 | 168545969 | C>T | *XCL1* | Intronic | 5:63 | Novel |
| 2 | 186839433 | C>A | *PLA2G4A* | Intronic | 41:81 | Novel |
| 2 | 186893200 | G>T | *PLA2G4A* | Intronic | 68:146 | Novel |
| 5 | 186937566 | A>G | *PLA2G4A* | Intronic | 6:16 | rs12063845 |
| 3 | 186951818 | T>C | *PLA2G4A* | Intronic | 5:52 | rs72714316 |
| 2 | 190106249 | A>G | *FAM5C* | Intronic | 8:87 | Novel |
| 4 | 190108001 | A>T | *FAM5C* | Intronic | 5:65 | Novel |
| 2 | 190115826 | C>A | *FAM5C* | Intronic | 29:57 | Novel |
| 5 | 190375699 | A>G | *FAM5C* | Intronic | 4:34 | Novel |
| 5 | 190375700 | G>C | *FAM5C* | Intronic | 4:34 | Novel |
| 7 | 215196988 | T>C | *KCNK2* | Intronic | 57:83 | Novel |
| 3 | 215197292 | G>T | *KCNK2* | Intronic | 38:68 | Novel |
| 3 | 215228313 | C>A | *KCNK2* | Intronic | 19:70 | Novel |
| 3 | 215229866 | G>A | *KCNK2* | Intronic | 15:12 | rs112717986 |
| 3 | 215231945 | C>T | *KCNK2* | Intronic | 57:190 | Novel |
| 6 | 215329426 | A>G | *KCNK2* | Intronic | 9:80 | rs12083839 |
| 5 | 215330562 | C>A | *KCNK2* | Intronic | 8:71 | Novel |
| 4 | 146902596 | G>T | *LINC00624* | Intronic (nc) | 12:0 | rs28626350 |
| 5 | 146935608 | G>T | *LINC00624* | Intronic (nc) | 5:31 | Novel |
| 3 | 148928846 | C>G | *LOC645166* | Intronic (nc) | 5:45 | Novel |
| 7 | 148928846 | C>G | *LOC645166* | Intronic (nc) | 3:35 | Novel |
| 7 | 148928972 | G>A | *LOC645166* | Intronic (nc) | 5:47 | rs184425987 |
| 7 | 148930113 | G>A | *LOC645166* | Intronic (nc) | 5:51 | rs2657993 |
| 2 | 149597293 | G>T | *LINC00623* | Intronic (nc) | 4:55 | rs2994152 |
|  |  |  | *LINC00869* |  |  |  |
| 5 | 149604899 | A>G | *LINC00623* | Intronic (nc) | 31:435 | Novel |
|  |  |  | *LINC00869* |  |  |  |
| 3 | 149653149 | G>A | *LINC00869* | Intronic (nc) | 3:5 | rs520185 |
| 5 | 186336479 | C>G | *MIR548F1* | Intronic (nc) | 3:39 | Novel |
| 3 | 186368837 | T>G | *MIR548F1* | Intronic (nc) | 31:27 | Novel |
| 3 | 186370610 | T>G | *MIR548F1* | Intronic (nc) | 22:56 | rs79691703 |
| 4 | 186370610 | T>G | *MIR548F1* | Intronic (nc) | 25:42 | rs79691703 |
| 3 | 186370618 | C>T | *MIR548F1* | Intronic (nc) | 11:63 | Novel |
| 5 | 190603638 | G>A | *LOC440704* | Intronic (nc) | 4:38 | Novel |
| 1 | 148759085 | C>G | *NBPF16* | Downstream | 4:14 | rs11578129 |
| 6 | 152386756 | G>A | *CRNN* | Upstream | 4:26 | Novel |
| 2 | 248814203 | A>G | *OR2T27* | Upstream | 5:52 | rs1770074 |
| 6 | 151668974 | T>G | *SNX27* | UTR3 | 23:53 | rs4617400 |
| 5 | 153395516 | A>G | *S100A7A* | UTR3 | 12:212 | rs116419971 |
| 5 | 153395523 | C>T | *S100A7A* | UTR3 | 13:207 | Novel |
| 5 | 153395543 | C>T | *S100A7A* | UTR3 | 11:224 | Novel |
| 2 | 162354696 | C>T | *C1orf226* | UTR3 | 38:30 | Novel |
| 1 | 173904764 | A>T | *RC3H1* | UTR3 | 5:83 | Novel |
| 4 | 142542196 | G>A |  | Intergenic | 3:34 | Novel |
| 3 | 142556091 | C>T |  | Intergenic | 8:118 | Novel |
| 5 | 142561077 | A>G |  | Intergenic | 5:48 | rs2841761 |
| 3 | 142601317 | G>A |  | Intergenic | 6:101 | Novel |
| 1 | 142601345 | C>A |  | Intergenic | 9:81 | Novel |
| 1 | 142601455 | T>A |  | Intergenic | 4:30 | Novel |
| 6 | 142601462 | C>A |  | Intergenic | 3:25 | Novel |
| 2 | 142607030 | C>T |  | Intergenic | 9:52 | rs11585021 |
| 2 | 142612092 | C>A |  | Intergenic | 30:293 | Novel |
| 2 | 142616242 | G>A |  | Intergenic | 8:203 | Novel |
| 6 | 142632408 | C>A |  | Intergenic | 4:16 | Novel |
| 6 | 142634692 | G>T |  | Intergenic | 10:160 | Novel |
| 4 | 142635325 | A>G |  | Intergenic | 4:39 | rs74571692 |
| 3 | 142654497 | T>C |  | Intergenic | 7:134 | Novel |
| 3 | 142726607 | A>G |  | Intergenic | 8:231 | rs2494818 |
| 3 | 142787539 | G>A |  | Intergenic | 7:98 | Novel |
| 3 | 142788013 | C>T |  | Intergenic | 6:109 | rs75404041 |
| 3 | 142808107 | G>T |  | Intergenic | 8:107 | Novel |
| 3 | 142808127 | C>T |  | Intergenic | 14:147 | rs78940654 |
| 2 | 142894472 | C>T |  | Intergenic | 3:19 | rs202053015 |
| 1 | 142894494 | T>C |  | Intergenic | 3:33 | Novel |
| 3 | 142896741 | G>T |  | Intergenic | 6:58 | rs138532427 |
| 6 | 142896741 | G>T |  | Intergenic | 4:43 | rs138532427 |
| 3 | 142898872 | G>C |  | Intergenic | 4:53 | rs3003097 |
| 5 | 142901656 | A>T |  | Intergenic | 5:21 | Novel |
| 3 | 142917818 | A>G |  | Intergenic | 4:89 | rs9660569 |
| 5 | 142917818 | A>G |  | Intergenic | 6:66 | rs9660569 |
| 1 | 143124333 | A>G |  | Intergenic | 4:80 | Novel |
| 1 | 143145252 | G>A |  | Intergenic | 6:26 | rs71256485 |
| 1 | 143164164 | C>T |  | Intergenic | 22:650 | Novel |
| 3 | 143186237 | G>C |  | Intergenic | 9:179 | rs71251594 |
| 6 | 143196416 | T>C |  | Intergenic | 4:16 | Novel |
| 6 | 143196427 | A>G |  | Intergenic | 4:16 | Novel |
| 4 | 143205515 | C>T |  | Intergenic | 5:94 | Novel |
| 6 | 143222285 | T>C |  | Intergenic | 30:214 | Novel |
| 6 | 143236183 | A>T |  | Intergenic | 7:46 | Novel |
| 4 | 143241190 | G>A |  | Intergenic | 6:104 | Novel |
| 3 | 143260213 | C>G |  | Intergenic | 5:45 | Novel |
| 3 | 143270487 | C>T |  | Intergenic | 3:17 | Novel |
| 6 | 143275137 | G>A |  | Intergenic | 9:61 | rs10127910 |
| 1 | 143275364 | C>G |  | Intergenic | 3:19 | rs76826390 |
| 2 | 143275633 | C>A |  | Intergenic | 5:22 | Novel |
| 1 | 143275723 | A>T |  | Intergenic | 3:33 | Novel |
| 4 | 143280598 | G>A |  | Intergenic | 3:4 | Novel |
| 5 | 143280882 | G>A |  | Intergenic | 5:82 | Novel |
| 7 | 143281502 | T>C |  | Intergenic | 3:37 | rs78603153 |
| 2 | 143520201 | T>C |  | Intergenic | 8:27 | rs75825911 |
| 3 | 143533977 | A>T |  | Intergenic | 7:54 | Novel |
| 7 | 143535214 | C>A |  | Intergenic | 40:577 | Novel |
| 1 | 143537367 | A>G |  | Intergenic | 4:26 | rs7527145 |
| 3 | 143537409 | A>G |  | Intergenic | 5:80 | Novel |
| 7 | 143537506 | C>T |  | Intergenic | 4:58 | rs7522904 |
| 3 | 143540991 | T>C |  | Intergenic | 14:484 | rs10442955 |
| 3 | 143915191 | T>C |  | Intergenic | 6:93 | Novel |
| 4 | 143915284 | G>A |  | Intergenic | 3:13 | rs9778075 |
| 6 | 144343616 | A>G |  | Intergenic | 6:25 | rs185467208 |
| 3 | 144533715 | G>A |  | Intergenic | 5:52 | rs513171 |
| 3 | 144539414 | A>T |  | Intergenic | 11:97 | rs533400 |
| 6 | 144549554 | G>A |  | Intergenic | 3:32 | rs111496274 |
| 3 | 144552478 | C>T |  | Intergenic | 9:133 | rs1699719 |
| 1 | 144582218 | G>T |  | Intergenic | 3:15 | rs1699736 |
| 3 | 146534676 | A>G |  | Intergenic | 61:66 | Novel |
| 6 | 147311521 | G>T |  | Intergenic | 31:135 | rs61809940 |
| 5 | 147740636 | A>C |  | Intergenic | 6:121 | Novel |
| 7 | 147740911 | A>G |  | Intergenic | 23:8 | rs112986919 |
| 5 | 147752770 | A>G |  | Intergenic | 13:285 | Novel |
| 5 | 147752771 | C>T |  | Intergenic | 14:285 | Novel |
| 5 | 147759291 | A>T |  | Intergenic | 34:537 | Novel |
| 5 | 147759312 | G>A |  | Intergenic | 19:641 | Novel |
| 5 | 147759545 | G>A |  | Intergenic | 22:379 | Novel |
| 5 | 147759547 | C>T |  | Intergenic | 20:381 | Novel |
| 5 | 147759599 | A>C |  | Intergenic | 20:408 | Novel |
| 5 | 147759600 | G>C |  | Intergenic | 22:407 | Novel |
| 5 | 147759766 | G>A |  | Intergenic | 25:433 | rs116575134 |
| 5 | 147759974 | T>C |  | Intergenic | 13:267 | Novel |
| 5 | 147760168 | T>G |  | Intergenic | 11:266 | rs17161672 |
| 5 | 147760193 | T>G |  | Intergenic | 11:215 | Novel |
| 5 | 147760221 | G>A |  | Intergenic | 7:147 | Novel |
| 5 | 147760255 | G>C |  | Intergenic | 8:139 | Novel |
| 5 | 147768238 | T>C |  | Intergenic | 12:291 | rs7514617 |
| 5 | 147768537 | C>A |  | Intergenic | 12:379 | Novel |
| 5 | 147768573 | G>A |  | Intergenic | 21:422 | Novel |
| 5 | 147768618 | T>C |  | Intergenic | 32:429 | Novel |
| 5 | 147768806 | G>C |  | Intergenic | 22:389 | Novel |
| 5 | 147768807 | G>T |  | Intergenic | 22:386 | Novel |
| 5 | 147768859 | A>G |  | Intergenic | 15:385 | Novel |
| 5 | 147769103 | A>T |  | Intergenic | 27:531 | Novel |
| 5 | 147776186 | G>C |  | Intergenic | 11:185 | Novel |
| 5 | 147777308 | C>T |  | Intergenic | 17:430 | Novel |
| 5 | 147777500 | C>A |  | Intergenic | 9:246 | rs200955731 |
| 5 | 147777511 | G>A |  | Intergenic | 13:230 | rs182771617 |
| 5 | 147795404 | G>A |  | Intergenic | 9:209 | Novel |
| 5 | 147795425 | C>G |  | Intergenic | 7:158 | rs141629877 |
| 7 | 148352169 | C>T |  | Intergenic | 4:66 | rs2938042 |
| 6 | 148533991 | G>A |  | Intergenic | 5:75 | rs72477340 |
| 6 | 148534052 | G>A |  | Intergenic | 5:47 | Novel |
| 4 | 148541670 | G>A |  | Intergenic | 4:51 | Novel |
| 5 | 148548578 | T>C |  | Intergenic | 4:96 | Novel |
| 1 | 148683384 | G>C |  | Intergenic | 3:40 | rs6657525 |
| 6 | 148683412 | C>T |  | Intergenic | 4:66 | Novel |
| 2 | 148850601 | T>A |  | Intergenic | 3:20 | Novel |
| 4 | 148877439 | A>G |  | Intergenic | 6:32 | rs381193 |
| 3 | 148878303 | G>A |  | Intergenic | 10:91 | rs200373911 |
| 3 | 148878311 | G>A |  | Intergenic | 9:102 | Novel |
| 1 | 148881284 | T>C |  | Intergenic | 7:76 | Novel |
| 6 | 148893314 | A>G |  | Intergenic | 9:50 | rs111671756 |
| 6 | 148895239 | G>C |  | Intergenic | 22:463 | Novel |
| 2 | 148910464 | C>G |  | Intergenic | 27:381 | Novel |
| 2 | 148923205 | A>T |  | Intergenic | 6:108 | Novel |
| 6 | 149024763 | C>T |  | Intergenic | 8:110 | Novel |
| 6 | 149024801 | G>C |  | Intergenic | 12:80 | Novel |
| 3 | 149028287 | C>A |  | Intergenic | 5:34 | Novel |
| 7 | 149028977 | G>T |  | Intergenic | 5:19 | rs55697632 |
| 1 | 149304729 | A>T |  | Intergenic | 5:32 | Novel |
| 6 | 149308176 | A>G |  | Intergenic | 52:317 | Novel |
| 4 | 149331084 | G>T |  | Intergenic | 7:86 | rs2749362 |
| 5 | 149419327 | T>A |  | Intergenic | 5:33 | Novel |
| 7 | 149454509 | T>C |  | Intergenic | 3:24 | rs9697441 |
| 6 | 149454509 | T>C |  | Intergenic | 4:28 | rs9697441 |
| 6 | 149454608 | T>C |  | Intergenic | 7:46 | rs9697408 |
| 3 | 149680070 | A>G |  | Intergenic | 5:115 | rs574230 |
| 3 | 149697596 | T>C |  | Intergenic | 7:95 | rs139718723 |
| 7 | 149699146 | A>G |  | Intergenic | 4:26 | rs4344365 |
| 6 | 149703075 | A>G |  | Intergenic | 4:63 | rs2084623 |
| 5 | 149751751 | A>G |  | Intergenic | 6:100 | rs113666509 |
| 5 | 150171203 | T>C |  | Intergenic | 4:73 | Novel |
| 5 | 150171326 | T>C |  | Intergenic | 6:124 | Novel |
| 5 | 150173805 | G>A |  | Intergenic | 7:175 | Novel |
| 4 | 150453991 | T>C |  | Intergenic | 8:20 | Novel |
| 7 | 186614115 | A>G |  | Intergenic | 26:68 | Novel |
| 5 | 189485821 | T>G |  | Intergenic | 4:37 | Novel |
| 5 | 189530929 | G>C |  | Intergenic | 4:56 | Novel |
| 5 | 189531097 | G>A |  | Intergenic | 11:77 | Novel |
| 5 | 189532655 | T>C |  | Intergenic | 14:74 | Novel |
| 5 | 189532817 | A>T |  | Intergenic | 6:48 | Novel |
| 5 | 189532823 | G>C |  | Intergenic | 10:44 | Novel |
| 5 | 189555286 | T>C |  | Intergenic | 3:5 | rs61818931 |
| 4 | 189560517 | T>G |  | Intergenic | 49:87 | Novel |
| 7 | 189618813 | T>C |  | Intergenic | 6:29 | rs2789897 |
| 5 | 189618813 | T>C |  | Intergenic | 3:3 | rs2789897 |
| 6 | 189768064 | A>T |  | Intergenic | 6:36 | Novel |
| 6 | 189841679 | A>G |  | Intergenic | 8:64 | Novel |
| 7 | 189862870 | T>A |  | Intergenic | 17:38 | Novel |
| 2 | 189981371 | C>T |  | Intergenic | 18:43 | Novel |
| 5 | 190851471 | C>T |  | Intergenic | 4:28 | rs7414066 |
| 4 | 190855304 | C>G |  | Intergenic | 39:82 | Novel |
| 5 | 224747843 | C>T |  | Intergenic | 7:171 | Novel |
| 5 | 224747844 | A>G |  | Intergenic | 7:172 | Novel |
| 5 | 224750829 | T>G |  | Intergenic | 9:217 | Novel |
| 6 | 249239504 | C>T |  | Intergenic | 4:48 | Novel |
| 4 | 249239760 | A>C |  | Intergenic | 5:47 | Novel |

Abbreviations: BCP ALL, B-cell precursor acute lymphoblastic leukemia; dbSNP, data base of single nucleotide polymorphisms; nc, non-coding; n-syn, non-synonymous; Nt, nucleotide; ref, reference; rs-ID, reference SNP-ID; SNV, single nucleotide variant; syn, synonymous; var, variant. ^a^Chromosome 1 positions according to the GRCh37 genome build. ^b^The dbSNP detection was based on build 138. ^c^These variants were verified by Sanger sequencing.
