# Supplementary material for: Mutation, methylation, and gene expression profiles in dup(1q)-positive pediatric B-cell precursor acute lymphoblastic leukemia
Source: Leukemia. 2018 Mar 12;32(10):2117–25. doi: 10.1038/s41375-018-0092-2 (PMC6170391; doi:10.1038/s41375-018-0092-2)
Supplement: Supplementary file 6 — Supplementary Table 6(DOCX 96 kb) [file 41375_2018_92_MOESM6_ESM.docx]

**Supplementary Table 6.** SNVs identified with Truseq custom amplification analysis in 111 BCP ALL cases

| *Case* | *dup(1q)* | *Reference^a^* | *Position^b^* | *Nt* | *Gene* | *Region* | *Read* | *dbSNP-id^c^* |
| --- | --- | --- | --- | --- | --- | --- | --- | --- |
| *No.* |  |  |  | *change* |  | *(type)* | *count* |  |
|  |  |  |  |  |  |  | (var:ref) |  |
| 1 | Yes | Panel | 152777863 | G>T | *LCE1C* | Exonic (n-syn SNV) | 60:68 | Novel |
| 1 | Yes | Panel | 152777868 | A>G | *LCE1C* | Exonic (syn SNV) | 67:60 | rs36107483 |
| 1 | Yes | Panel | 156640503 | C>T | *NES* | Exonic syn SNV | 154:257 | rs3828043 |
| 1 | Yes | Panel | 156641537 | C>T | *NES* | Exonic n-syn SNV | 546:322 | rs951781 |
| 1 | Yes | Panel | 196205117 | C>A | *KCNT2* | Exonic n-syn SNV | 593:353 | Novel |
| 1 | Yes | Panel | 226555302 | A>G | *PARP1* | Exonic n-syn SNV | 349:338 | rs1136410 |
| 1 | Yes | Panel | 226589958 | G>A | *PARP1* | Exonic syn SNV | 241:240 | rs1805404 |
| 1 | Yes | Panel | 226595647 | C>G | *PARP1* | UTR5 | 28:11 | rs907187 |
| 1 | Yes | Panel | 240256822 | C>A | *FMN2* | Exonic syn SNV | 243:377 | rs10926124 |
| 1 | Yes | Panel | 240341258 | C>A | *FMN2* | Exonic n-syn SNV | 186:757 | NA |
| 1 | Yes | Panel | 240341259 | C>A | *FMN2* | Exonic syn SNV | 120:821 | Novel |
| 1 | Yes | Panel | 240341268 | A>G | *FMN2* | Exonic syn SNV | 47:867 | rs3765588 |
| 1 | Yes | Panel | 240370752 | T>A | *FMN2* | Exonic syn SNV | 20:490 | Novel |
| 1 | Yes | Panel | 240497193 | A>T | *FMN2* | Exonic n-syn SNV | 305:482 | Novel |
| 2 | Yes | Paired | 240370752 | T>A | *FMN2* | Exonic syn SNV | 34:752 | Novel |
| 3 | Yes | Paired | 240370752 | T>A | *FMN2* | Exonic syn SNV | 21:579 | Novel |
| 5 | Yes | Paired | 240370752 | T>A | *FMN2* | Exonic syn SNV | 42:915 | Novel |
| 7 | Yes | Paired | 240370752 | T>A | *FMN2* | Exonic syn SNV | 30:926 | Novel |
| 8 | Yes | Panel | 152777868 | A>G | *LCE1C* | Exonic syn SNV | 59:0 | rs36107483 |
| 8 | Yes | Panel | 156642823 | G>C | *NES* | Exonic n-syn SNV | 15:96 | NA |
| 8 | Yes | Panel | 226570840 | T>C | *PARP1* | Exonic syn SNV | 694:6 | rs1805415 |
| 8 | Yes | Panel | 240256822 | C>A | *FMN2* | Exonic syn SNV | 257:527 | rs10926124 |
| 8 | Yes | Panel | 240370752 | T>A | *FMN2* | Exonic syn SNV | 45:919 | Novel |
| 9 | Yes | Panel | 152777386 | G>T | *LCE1C* | UTR3 | 96:616 | Novel |
| 9 | Yes | Panel | 156639636 | G>A | *NES* | Exonic syn SNV | 196:139 | rs11549292 |
| 9 | Yes | Panel | 156640308 | C>T | *NES* | Exonic syn SNV | 451:437 | rs3748571 |
| 9 | Yes | Panel | 156640503 | C>T | *NES* | Exonic syn SNV | 352:261 | rs3828043 |
| 9 | Yes | Panel | 156642823 | G>C | *NES* | Exonic n-syn SNV | 12:73 | NA |
| 9 | Yes | Panel | 169365148 | A>T | *BLZF1* | UTR3 | 31:151 | Novel |
| 9 | Yes | Panel | 226552733 | A>C | *PARP1* | Exonic syn SNV | 182:710 | NA |
| 9 | Yes | Panel | 226570840 | T>C | *PARP1* | Exonic syn SNV | 790:3 | rs1805415 |
| 9 | Yes | Panel | 226595754 | A>C | *PARP1* | UTR5 | 83:430 | Novel |
| 9 | Yes | Panel | 240601397 | A>T | *FMN2* | Exonic syn SNV | 89:876 | Novel |
| 10 | Yes | Panel | 152777386 | G>T | *LCE1C* | UTR3 | 104:627 | Novel |
| 10 | Yes | Panel | 152777868 | A>G | *LCE1C* | Exonic syn SNV | 54:40 | rs36107483 |
| 10 | Yes | Panel | 156639300 | A>T | *NES* | Exonic syn SNV | 68:887 | Novel |
| 10 | Yes | Panel | 196577323 | A>G | *KCNT2* | Intronic | 281:659 | Novel |
| 10 | Yes | Panel | 240341258 | C>A | *FMN2* | Exonic n-syn SNV | 180:737 | NA |
| 10 | Yes | Panel | 240341259 | C>A | *FMN2* | Exonic syn SNV | 118:800 | Novel |
| 10 | Yes | Panel | 240370752 | T>A | *FMN2* | Exonic syn SNV | 28:691 | Novel |
| 11 | Yes | Panel | 152777868 | A>G | *LCE1C* | Exonic syn SNV | 45:48 | rs36107483 |
| 11 | Yes | Panel | 156639636 | G>A | *NES* | Exonic syn SNV | 167:123 | rs11549292 |
| 11 | Yes | Panel | 156640308 | C>T | *NES* | Exonic syn SNV | 421:236 | rs3748571 |
| 11 | Yes | Panel | 156640503 | C>T | *NES* | Exonic syn SNV | 245:203 | rs3828043 |
| 11 | Yes | Panel | 156640678 | G>A | *NES* | Exonic n-syn SNV | 338:233 | rs2886443 |
| 11 | Yes | Panel | 156641537 | C>T | *NES* | Exonic n-syn SNV | 351:568 | rs951781 |
| 11 | Yes | Panel | 226552733 | A>C | *PARP1* | Exonic syn SNV | 252:707 | NA |
| 11 | Yes | Panel | 226555302 | A>G | *PARP1* | Exonic n-syn SNV | 259:364 | rs1136410 |
| 11 | Yes | Panel | 226589958 | G>A | *PARP1* | Exonic syn SNV | 359:460 | rs1805404 |
| 11 | Yes | Panel | 226595647 | C>G | *PARP1* | UTR5 | 27:15 | rs907187 |
| 11 | Yes | Panel | 240370752 | T>A | *FMN2* | Exonic syn SNV | 26:551 | Novel |
| 11 | Yes | Panel | 240458201 | T>C | *FMN2* | Intronic | 280:390 | rs200957712 |
| 11 | Yes | Panel | 240519208 | G>A | *FMN2* | Exonic n-syn SNV | 113:117 | NA |
| 13 | Yes | Paired | 240370752 | T>A | *FMN2* | Exonic syn SNV | 18:445 | Novel |
| 14 | Yes | Paired | 156638913 | T>C | *NES* | UTR3 | 31:909 | Novel |
| 14 | Yes | Paired | 240256008 | A>C | *FMN2* | Exonic n-syn SNV | 41:476 | Novel |
| 15 | Yes | Panel | 152777868 | A>G | *LCE1C* | Exonic syn SNV | 22:12 | rs36107483 |
| 15 | Yes | Panel | 156639636 | G>A | *NES* | Exonic syn SNV | 178:145 | rs11549292 |
| 15 | Yes | Panel | 156640308 | C>T | *NES* | Exonic syn SNV | 382:388 | rs3748571 |
| 15 | Yes | Panel | 156640503 | C>T | *NES* | Exonic syn SNV | 350:362 | rs3828043 |
| 15 | Yes | Panel | 156641537 | C>T | *NES* | Exonic n-syn SNV | 387:487 | rs951781 |
| 15 | Yes | Panel | 156642823 | G>C | *NES* | Exonic n-syn SNV | 21:98 | NA |
| 15 | Yes | Panel | 226552733 | A>C | *PARP1* | Exonic syn SNV | 226:716 | NA |
| 15 | Yes | Panel | 226555302 | A>G | *PARP1* | Exonic n-syn SNV | 779:1 | rs1136410 |
| 15 | Yes | Panel | 226595647 | C>G | *PARP1* | UTR5 | 68:0 | rs907187 |
| 15 | Yes | Panel | 240256822 | C>A | *FMN2* | Exonic syn SNV | 253:528 | rs10926124 |
| 15 | Yes | Panel | 240492414 | T>C | *FMN2* | Exonic syn SNV | 674:273 | rs6677726 |
| 16 | Yes | Panel | 152777908 | C>T | *LCE1C* | Exonic n-syn SNV | 32:36 | rs2006940 |
| 16 | Yes | Panel | 156641537 | C>T | *NES* | Exonic n-syn SNV | 398:496 | rs951781 |
| 16 | Yes | Panel | 156647152 | A>C | *NES* | UTR5 | 57:480 | Novel |
| 16 | Yes | Panel | 196197373 | C>T | *KCNT2* | Exonic n-syn SNV | 35:553 | NA |
| 16 | Yes | Panel | 226552733 | A>C | *PARP1* | Exonic syn SNV | 212:695 | NA |
| 16 | Yes | Panel | 226570840 | T>C | *PARP1* | Exonic syn SNV | 883:2 | rs1805415 |
| 16 | Yes | Panel | 240341258 | C>A | *FMN2* | Exonic n-syn SNV | 174:756 | NA |
| 16 | Yes | Panel | 240341259 | C>A | *FMN2* | Exonic syn SNV | 102:830 | Novel |
| 16 | Yes | Panel | 240370752 | T>A | *FMN2* | Exonic syn SNV | 33:781 | Novel |
| 16 | Yes | Panel | 240492734 | G>A | *FMN2* | Exonic n-syn SNV | 748:217 | rs3795677 |
| 28 | No | Paired | 240370752 | T>A | *FMN2* | Exonic syn SNV | 23:567 | Novel |
| 29 | No | Paired | 152777964 | G>A | *LCE1C* | UTR5 | 43:90 | rs17624493 |
| 29 | No | Paired | 226595647 | C>G | *PARP1* | UTR5 | 23:45 | rs907187 |
| 29 | No | Paired | 240370752 | T>A | *FMN2* | Exonic syn SNV | 29:926 | Novel |
| 30 | No | Paired | 240370752 | T>A | *FMN2* | Exonic syn SNV | 38:923 | Novel |
| 31 | No | Paired | 240370752 | T>A | *FMN2* | Exonic syn SNV | 29:597 | Novel |
| 32 | No | Panel | 156639636 | G>A | *NES* | Exonic syn SNV | 222:289 | rs11549292 |
| 32 | No | Panel | 156640308 | C>T | *NES* | Exonic syn SNV | 497:408 | rs3748571 |
| 32 | No | Panel | 156642823 | G>C | *NES* | Exonic n-syn SNV | 16:77 | NA |
| 32 | No | Panel | 226570840 | T>C | *PARP1* | Exonic syn SNV | 663:3 | rs1805415 |
| 32 | No | Panel | 226595754 | A>C | *PARP1* | UTR5 | 56:247 | Novel |
| 32 | No | Panel | 240256822 | C>A | *FMN2* | Exonic syn SNV | 342:329 | rs10926124 |
| 32 | No | Panel | 240370752 | T>A | *FMN2* | Exonic syn SNV | 26:943 | Novel |
| 32 | No | Panel | 240519208 | G>A | *FMN2* | Exonic n-syn SNV | 147:297 | NA |
| 32 | No | Panel | 240601397 | A>T | *FMN2* | Exonic syn SNV | 100:871 | Novel |
| 33 | No | Panel | 152777386 | G>T | *LCE1C* | UTR3 | 82:603 | Novel |
| 33 | No | Panel | 152777868 | A>G | *LCE1C* | Exonic syn SNV | 232:2 | rs36107483 |
| 33 | No | Panel | 156639636 | G>A | *NES* | Exonic syn SNV | 131:160 | rs11549292 |
| 33 | No | Panel | 156640308 | C>T | *NES* | Exonic syn SNV | 410:368 | rs3748571 |
| 33 | No | Panel | 156641537 | C>T | *NES* | Exonic n-syn SNV | 313:516 | rs951781 |
| 33 | No | Panel | 156642823 | G>C | *NES* | Exonic n-syn SNV | 20:125 | NA |
| 33 | No | Panel | 226567556 | T>G | *PARP1* | Intronic | 53:805 | Novel |
| 33 | No | Panel | 226570840 | T>C | *PARP1* | Exonic syn SNV | 899:5 | rs1805415 |
| 33 | No | Panel | 240341258 | C>A | *FMN2* | Exonic n-syn SNV | 199:721 | NA |
| 33 | No | Panel | 240341259 | C>A | *FMN2* | Exonic syn SNV | 131:790 | Novel |
| 33 | No | Panel | 240492734 | G>A | *FMN2* | Exonic n-syn SNV | 356:580 | rs3795677 |
| 33 | No | Panel | 240601397 | A>T | *FMN2* | Exonic syn SNV | 113:853 | Novel |
| 34 | No | Paired | 240370752 | T>A | *FMN2* | Exonic syn SNV | 35:408 | Novel |
| 35 | No | Panel | 156640678 | G>A | *NES* | Exonic n-syn SNV | 403:338 | rs2886443 |
| 35 | No | Panel | 156641537 | C>T | *NES* | Exonic n-syn SNV | 407:450 | rs951781 |
| 35 | No | Panel | 156641871 | A>T | *NES* | Exonic syn SNV | 61:870 | Novel |
| 35 | No | Panel | 156642823 | G>C | *NES* | Exonic n-syn SNV | 15:72 | NA |
| 35 | No | Panel | 226552733 | A>C | *PARP1* | Exonic syn SNV | 162:714 | NA |
| 35 | No | Panel | 226570840 | T>C | *PARP1* | Exonic syn SNV | 828:6 | rs1805415 |
| 35 | No | Panel | 240256822 | C>A | *FMN2* | Exonic syn SNV | 308:400 | rs10926124 |
| 35 | No | Panel | 240370752 | T>A | *FMN2* | Exonic syn SNV | 50:911 | Novel |
| 36 | No | Panel | 152777908 | C>T | *LCE1C* | Exonic n-syn SNV | 34:45 | rs2006940 |
| 36 | No | Panel | 156639636 | G>A | *NES* | Exonic syn SNV | 165:201 | rs11549292 |
| 36 | No | Panel | 156640308 | C>T | *NES* | Exonic syn SNV | 312:392 | rs3748571 |
| 36 | No | Panel | 156640503 | C>T | *NES* | Exonic syn SNV | 174:206 | rs3828043 |
| 36 | No | Panel | 156641537 | C>T | *NES* | Exonic n-syn SNV | 496:394 | rs951781 |
| 36 | No | Panel | 156642823 | G>C | *NES* | Exonic n-syn SNV | 16:48 | NA |
| 36 | No | Panel | 226570840 | T>C | *PARP1* | Exonic syn SNV | 620:4 | rs1805415 |
| 36 | No | Panel | 226595754 | A>C | *PARP1* | UTR5 | 60:324 | Novel |
| 36 | No | Panel | 240256822 | C>A | *FMN2* | Exonic syn SNV | 221:247 | rs10926124 |
| 36 | No | Panel | 240370752 | T>A | *FMN2* | Exonic syn SNV | 27:496 | Novel |
| 36 | No | Panel | 240492734 | G>A | *FMN2* | Exonic n-syn SNV | 717:248 | rs3795677 |
| 37 | No | Panel | 156639636 | G>A | *NES* | Exonic syn SNV | 183:233 | rs11549292 |
| 37 | No | Panel | 156640308 | C>T | *NES* | Exonic syn SNV | 461:402 | rs3748571 |
| 37 | No | Panel | 156641537 | C>T | *NES* | Exonic n-syn SNV | 453:439 | rs951781 |
| 37 | No | Panel | 226552733 | A>C | *PARP1* | Exonic syn SNV | 229:697 | NA |
| 37 | No | Panel | 226570840 | T>C | *PARP1* | Exonic syn SNV | 752:9 | rs1805415 |
| 37 | No | Panel | 240256822 | C>A | *FMN2* | Exonic syn SNV | 315:404 | rs10926124 |
| 37 | No | Panel | 240370752 | T>A | *FMN2* | Exonic syn SNV | 37:935 | Novel |
| 38 | No | Panel | 156641537 | C>T | *NES* | Exonic n-syn SNV | 425:480 | rs951781 |
| 38 | No | Panel | 226552733 | A>C | *PARP1* | Exonic syn SNV | 175:714 | NA |
| 38 | No | Panel | 226595647 | C>G | *PARP1* | UTR5 | 98:1 | rs907187 |
| 38 | No | Panel | 226595754 | A>C | *PARP1* | UTR5 | 78:389 | Novel |
| 38 | No | Panel | 240341259 | C>A | *FMN2* | Exonic syn SNV | 119:831 | Novel |
| 38 | No | Panel | 240370752 | T>A | *FMN2* | Exonic syn SNV | 34:932 | Novel |
| 38 | No | Panel | 240601397 | A>T | *FMN2* | Exonic syn SNV | 75:891 | Novel |
| 40 | No | Paired | 240370752 | T>A | *FMN2* | Exonic syn SNV | 18:496 | Novel |
| 41 | No | Panel | 156640678 | G>A | *NES* | Exonic n-syn SNV | 65:48 | rs2886443 |
| 41 | No | Panel | 156641537 | C>T | *NES* | Exonic n-syn SNV | 409:467 | rs951781 |
| 41 | No | Panel | 226552733 | A>C | *PARP1* | Exonic syn SNV | 235:709 | NA |
| 41 | No | Panel | 226570840 | T>C | *PARP1* | Exonic syn SNV | 564:5 | rs1805415 |
| 41 | No | Panel | 240256822 | C>A | *FMN2* | Exonic syn SNV | 325:345 | rs10926124 |
| 41 | No | Panel | 240519208 | G>A | *FMN2* | Exonic n-syn SNV | 95:111 | NA |
| 42 | No | Panel | 152777386 | G>T | *LCE1C* | UTR3 | 87:595 | Novel |
| 42 | No | Panel | 152777868 | A>G | *LCE1C* | Exonic syn SNV | 37:39 | rs36107483 |
| 42 | No | Panel | 156639300 | A>T | *NES* | Exonic syn SNV | 84:881 | Novel |
| 42 | No | Panel | 156639636 | G>A | *NES* | Exonic syn SNV | 267:246 | rs11549292 |
| 42 | No | Panel | 226570840 | T>C | *PARP1* | Exonic syn SNV | 822:3 | rs1805415 |
| 42 | No | Panel | 240256822 | C>A | *FMN2* | Exonic syn SNV | 423:434 | rs10926124 |
| 42 | No | Panel | 240370362 | A>T | *FMN2* | Exonic n-syn SNV | 63:855 | Novel |
| 42 | No | Panel | 240370752 | T>A | *FMN2* | Exonic syn SNV | 30:940 | Novel |
| 44 | No | Panel | 152777868 | A>G | *LCE1C* | Exonic syn SNV | 81:108 | rs36107483 |
| 44 | No | Panel | 156639636 | G>A | *NES* | Exonic syn SNV | 197:1 | rs11549292 |
| 44 | No | Panel | 156640678 | G>A | *NES* | Exonic n-syn SNV | 536:0 | rs2886443 |
| 44 | No | Panel | 156642823 | G>C | *NES* | Exonic n-syn SNV | 10:30 | NA |
| 44 | No | Panel | 226595754 | A>C | *PARP1* | UTR5 | 63:339 | Novel |
| 44 | No | Panel | 240256822 | C>A | *FMN2* | Exonic syn SNV | 235:253 | rs10926124 |
| 44 | No | Panel | 240370752 | T>A | *FMN2* | Exonic syn SNV | 23:496 | Novel |
| 44 | No | Panel | 240601397 | A>T | *FMN2* | Exonic syn SNV | 80:892 | Novel |
| 45 | No | Panel | 156640503 | C>T | *NES* | Exonic syn SNV | 721:4 | rs3828043 |
| 45 | No | Panel | 226570840 | T>C | *PARP1* | Exonic syn SNV | 376:339 | rs1805415 |
| 45 | No | Panel | 226595647 | C>G | *PARP1* | UTR5 | 55:0 | rs907187 |
| 45 | No | Panel | 226595754 | A>C | *PARP1* | UTR5 | 86:408 | Novel |
| 45 | No | Panel | 240256822 | C>A | *FMN2* | Exonic syn SNV | 360:373 | rs10926124 |
| 45 | No | Panel | 240519208 | G>A | *FMN2* | Exonic n-syn SNV | 109:530 | NA |
| 45 | No | Panel | 240601397 | A>T | *FMN2* | Exonic syn SNV | 103:847 | Novel |
| 80 | No | Panel | 156639636 | G>A | *NES* | Exonic syn SNV | 120:238 | rs11549292 |
| 80 | No | Panel | 156640308 | C>T | *NES* | Exonic syn SNV | 186:207 | rs3748571 |
| 80 | No | Panel | 156640503 | C>T | *NES* | Exonic syn SNV | 420:528 | rs3828043 |
| 80 | No | Panel | 156640678 | G>A | *NES* | Exonic n-syn SNV | 382:513 | rs2886443 |
| 80 | No | Panel | 156641537 | C>T | *NES* | Exonic n-syn SNV | 394:531 | rs951781 |
| 80 | No | Panel | 156646668 | A>G | *NES* | Exonic n-syn SNV | 765:2 | rs4278369 |
| 80 | No | Panel | 226552733 | A>C | *PARP1* | Exonic syn SNV | 156:703 | NA |
| 85 | No | Paired | 152777964 | G>A | *LCE1C* | UTR5 | 10:33 | rs17624493 |
| 85 | No | Paired | 156640503 | C>T | *NES* | Exonic syn SNV | 531:3 | rs3828043 |
| 85 | No | Paired | 196311253 | A>G | *KCNT2* | Exonic syn SNV | 461:477 | rs145756671 |
| 85 | No | Paired | 240256895 | G>A | *FMN2* | Exonic n-syn SNV | 4:13 | Novel |
| 85 | No | Paired | 240341268 | A>G | *FMN2* | Exonic syn SNV | 139:766 | rs3765588 |
| 85 | No | Paired | 240370752 | T>A | *FMN2* | Exonic syn SNV | 22:491 | Novel |
| 85 | No | Paired | 240497265 | A>C | *FMN2* | Intronic | 250:272 | rs10157874 |
| 86 | No | Panel | 152777868 | A>G | *LCE1C* | Exonic syn SNV | 39:38 | rs36107483 |
| 86 | No | Panel | 156640308 | C>T | *NES* | Exonic syn SNV | 865:1 | rs3748571 |
| 86 | No | Panel | 226552733 | A>C | *PARP1* | Exonic syn SNV | 236:727 | NA |
| 86 | No | Panel | 226570840 | T>C | *PARP1* | Exonic syn SNV | 831:5 | rs1805415 |
| 86 | No | Panel | 240256822 | C>A | *FMN2* | Exonic syn SNV | 403:343 | rs10926124 |
| 86 | No | Panel | 240341258 | C>A | *FMN2* | Exonic n-syn SNV | 178:758 | NA |
| 86 | No | Panel | 240341259 | C>A | *FMN2* | Exonic syn SNV | 115:824 | Novel |
| 86 | No | Panel | 240601397 | A>T | *FMN2* | Exonic syn SNV | 92:882 | Novel |
| 87 | No | Paired | 196205189 | C>T^d^ | *KCNT2* | Exonic n-syn SNV | 163:308 | Novel |
| 89 | No | Paired | 240370752 | T>A | *FMN2* | Exonic syn SNV | 30:533 | Novel |
| 90 | No | Paired | 240370752 | T>A | *FMN2* | Exonic syn SNV | 19:545 | Novel |
| 91 | No | Paired | 240255608 | A>T | *FMN2* | Exonic stopgain | 12:118 | Novel |
| 95 | No | Panel | 169337486 | A>G | *BLZF1* | UTR5 | 349:357 | Novel |
| 95 | No | Panel | 226552733 | A>C | *PARP1* | Exonic syn SNV | 121:694 | NA |
| 95 | No | Panel | 240256008 | A>C | *FMN2* | Exonic n-syn SNV | 42:536 | Novel |
| 95 | No | Panel | 240256822 | C>A | *FMN2* | Exonic syn SNV | 376:365 | rs10926124 |
| 95 | No | Panel | 240341258 | C>A | *FMN2* | Exonic n-syn SNV | 165:774 | NA |
| 95 | No | Panel | 240341259 | C>A | *FMN2* | Exonic syn SNV | 132:809 | Novel |
| 95 | No | Panel | 240370752 | T>A | *FMN2* | Exonic syn SNV | 31:927 | Novel |
| 95 | No | Panel | 240492734 | G>A | *FMN2* | Exonic n-syn SNV | 729:214 | rs3795677 |
| 97 | No | Panel | 226552733 | A>C | *PARP1* | Exonic syn SNV | 146:692 | NA |
| 97 | No | Panel | 226555302 | A>G | *PARP1* | Exonic n-syn SNV | 393:477 | rs1136410 |
| 97 | No | Panel | 226589958 | G>A | *PARP1* | Exonic syn SNV | 452:510 | rs1805404 |
| 97 | No | Panel | 226595647 | C>G | *PARP1* | UTR5 | 42:34 | rs907187 |
| 97 | No | Panel | 240341258 | C>A | *FMN2* | Exonic n-syn SNV | 149:794 | NA |
| 97 | No | Panel | 240341259 | C>A | *FMN2* | Exonic syn SNV | 112:836 | Novel |
| 97 | No | Panel | 240601397 | A>T | *FMN2* | Exonic syn SNV | 76:889 | Novel |
| 100 | No | Paired | 240370752 | T>A | *FMN2* | Exonic syn SNV | 30:917 | Novel |
| 101 | No | Panel | 156642823 | G>C | *NES* | Exonic n-syn SNV | 19:78 | NA |
| 101 | No | Panel | 169338721 | A>T | *BLZF1* | UTR5 | 60:900 | Novel |
| 101 | No | Panel | 226552771 | A>G | *PARP1* | Exonic n-syn SNV | 43:811 | NA |
| 101 | No | Panel | 226595754 | A>C | *PARP1* | UTR5 | 152:713 | Novel |
| 101 | No | Panel | 240256822 | C>A | *FMN2* | Exonic syn SNV | 377:476 | rs10926124 |
| 101 | No | Panel | 240341268 | A>G | *FMN2* | Exonic syn SNV | 164:751 | rs3765588 |
| 101 | No | Panel | 240370362 | A>T | *FMN2* | Exonic n-syn SNV | 80:848 | Novel |
| 102 | No | Paired | 240370752 | T>A | *FMN2* | Exonic syn SNV | 43:910 | Novel |
| 103 | No | Paired | 240370752 | T>A | *FMN2* | Exonic syn SNV | 30:837 | Novel |
| 105 | No | Paired | 240370752 | T>A | *FMN2* | Exonic syn SNV | 29:901 | Novel |
| 106 | No | Panel | 152777868 | A>G | *LCE1C* | Exonic syn SNV | 78:5 | rs36107483 |
| 106 | No | Panel | 156640069 | T>C | *NES* | Exonic n-syn SNV | 39:830 | Novel |
| 106 | No | Panel | 156642823 | G>C | *NES* | Exonic n-syn SNV | 14:87 | NA |
| 106 | No | Panel | 226552733 | A>C | *PARP1* | Exonic syn SNV | 244:714 | NA |
| 106 | No | Panel | 226555302 | A>G | *PARP1* | Exonic n-syn SNV | 354:343 | rs1136410 |
| 106 | No | Panel | 226570840 | T>C | *PARP1* | Exonic syn SNV | 363:365 | rs1805415 |
| 106 | No | Panel | 226595647 | C>G | *PARP1* | UTR5 | 52:68 | rs907187 |
| 106 | No | Panel | 226595754 | A>C | *PARP1* | UTR5 | 113:622 | Novel |
| 106 | No | Panel | 240370752 | T>A | *FMN2* | Exonic syn SNV | 31:933 | Novel |
| 106 | No | Panel | 240519208 | G>A | *FMN2* | Exonic n-syn SNV | 183:237 | NA |
| 106 | No | Panel | 240519229 | T>A | *FMN2* | Intronic | 143:453 | Novel |
| 107 | No | Panel | 152777868 | A>G | *LCE1C* | Exonic syn SNV | 48:67 | rs36107483 |
| 107 | No | Panel | 152777908 | C>T | *LCE1C* | Exonic n-syn SNV | 69:44 | rs2006940 |
| 107 | No | Panel | 156639300 | A>T | *NES* | Exonic syn SNV | 88:864 | Novel |
| 107 | No | Panel | 156639310 | A>T | *NES* | Exonic n-syn SNV | 66:885 | Novel |
| 107 | No | Panel | 156641537 | C>T | *NES* | Exonic n-syn SNV | 424:452 | rs951781 |
| 107 | No | Panel | 156642823 | G>C | *NES* | Exonic n-syn SNV | 16:71 | NA |
| 107 | No | Panel | 226552733 | A>C | *PARP1* | Exonic syn SNV | 165:686 | NA |
| 107 | No | Panel | 226570840 | T>C | *PARP1* | Exonic syn SNV | 809:3 | rs1805415 |
| 107 | No | Panel | 226595647 | C>G | *PARP1* | UTR5 | 95:45 | rs907187 |
| 107 | No | Panel | 240256822 | C>A | *FMN2* | Exonic syn SNV | 379:363 | rs10926124 |
| 107 | No | Panel | 240601397 | A>T | *FMN2* | Exonic syn SNV | 83:886 | Novel |
| 110 | No | Paired | 240370752 | T>A | *FMN2* | Exonic syn SNV | 36:930 | Novel |
| 112 | No | Paired | 240370752 | T>A | *FMN2* | Exonic syn SNV | 36:919 | Novel |
| 114 | No | Paired | 240341268 | A>G | *FMN2* | Exonic syn SNV | 82:833 | rs3765588 |
| 114 | No | Paired | 240370752 | T>A | *FMN2* | Exonic syn SNV | 38:909 | Novel |
| 116 | No | Paired | 240370752 | T>A | *FMN2* | Exonic syn SNV | 35:932 | Novel |
| 119 | No | Paired | 240341229 | A>G | *FMN2* | Exonic syn SNV | 6:33 | Novel |
| 119 | No | Paired | 240341337 | T>A | *FMN2* | Exonic n-syn SNV | 7:33 | Novel |
| 120 | No | Panel | 156639636 | G>A | *NES* | Exonic syn SNV | 154:157 | rs11549292 |
| 120 | No | Panel | 156640308 | C>T | *NES* | Exonic syn SNV | 477:403 | rs3748571 |
| 120 | No | Panel | 156640678 | G>A | *NES* | Exonic n-syn SNV | 462:301 | rs2886443 |
| 120 | No | Panel | 156641537 | C>T | *NES* | Exonic n-syn SNV | 375:490 | rs951781 |
| 120 | No | Panel | 226552733 | A>C | *PARP1* | Exonic syn SNV | 146:720 | NA |
| 120 | No | Panel | 240370752 | T>A | *FMN2* | Exonic syn SNV | 31:919 | Novel |
| 120 | No | Panel | 240601397 | A>T | *FMN2* | Exonic syn SNV | 93:870 | Novel |
| 121 | No | Paired | 156639310 | A>T | *NES* | Exonic n-syn SNV | 68:894 | Novel |
| 121 | No | Paired | 240370752 | T>A | *FMN2* | Exonic syn SNV | 31:927 | Novel |
| 122 | No | Panel | 152777386 | G>T | *LCE1C* | UTR3 | 107:621 | Novel |
| 122 | No | Panel | 156640803 | C>T | *NES* | Exonic syn SNV | 51:819 | Novel |
| 122 | No | Panel | 156641537 | C>T | *NES* | Exonic n-syn SNV | 411:470 | rs951781 |
| 122 | No | Panel | 226595754 | A>C | *PARP1* | UTR5 | 90:393 | Novel |
| 122 | No | Panel | 240256822 | C>A | *FMN2* | Exonic syn SNV | 384:423 | rs10926124 |
| 122 | No | Panel | 240341258 | C>A | *FMN2* | Exonic n-syn SNV | 182:767 | NA |
| 122 | No | Panel | 240341259 | C>A | *FMN2* | Exonic syn SNV | 121:826 | Novel |
| 122 | No | Panel | 240519208 | G>A | *FMN2* | Exonic n-syn SNV | 219:199 | NA |
| 123 | No | Paired | 240370752 | T>A | *FMN2* | Exonic syn SNV | 33:936 | Novel |
| 126 | No | Paired | 240370752 | T>A | *FMN2* | Exonic syn SNV | 27:745 | Novel |
| 127 | No | Paired | 240370752 | T>A | *FMN2* | Exonic syn SNV | 34:921 | Novel |
| 128 | No | Panel | 152777386 | G>T | *LCE1C* | UTR3 | 107:673 | Novel |
| 128 | No | Panel | 152777666 | T>A | *LCE1C* | Exonic n-syn SNV | 17:51 | Novel |
| 128 | No | Panel | 226552733 | A>C | *PARP1* | Exonic syn SNV | 261:694 | NA |
| 128 | No | Panel | 226555302 | A>G | *PARP1* | Exonic n-syn SNV | 737:1 | rs1136410 |
| 128 | No | Panel | 226595647 | C>G | *PARP1* | UTR5 | 173:0 | rs907187 |
| 128 | No | Panel | 226595754 | A>C | *PARP1* | UTR5 | 136:641 | Novel |
| 128 | No | Panel | 240341268 | A>G | *FMN2* | Exonic syn SNV | 526:5 | rs3765588 |
| 128 | No | Panel | 240370752 | T>A | *FMN2* | Exonic syn SNV | 38:923 | Novel |
| 128 | No | Panel | 240492414 | T>C | *FMN2* | Exonic syn SNV | 931:4 | rs6677726 |
| 128 | No | Panel | 240601397 | A>T | *FMN2* | Exonic syn SNV | 76:886 | Novel |
| 131 | No | Panel | 152777386 | G>T | *LCE1C* | UTR3 | 111:585 | Novel |
| 131 | No | Panel | 152777666 | T>A | *LCE1C* | Exonic n-syn SNV | 15:45 | Novel |
| 131 | No | Panel | 156639300 | A>T | *NES* | Exonic syn SNV | 92:834 | Novel |
| 131 | No | Panel | 156639310 | A>T | *NES* | Exonic n-syn SNV | 69:857 | Novel |
| 131 | No | Panel | 156640520 | A>T | *NES* | Exonic n-syn SNV | 117:823 | Novel |
| 131 | No | Panel | 156640536 | A>T | *NES* | Exonic syn SNV | 101:825 | Novel |
| 131 | No | Panel | 156641537 | C>T | *NES* | Exonic n-syn SNV | 421:398 | rs951781 |
| 133 | No | Paired | 240370752 | T>A | *FMN2* | Exonic syn SNV | 17:502 | Novel |
| 134 | No | Paired | 240370659 | T>A | *FMN2* | Exonic n-syn SNV | 54:713 | Novel |
| 134 | No | Paired | 240370708 | T>C | *FMN2* | Exonic n-syn SNV | 35:888 | Novel |
| 134 | No | Paired | 240370752 | T>A | *FMN2* | Exonic syn SNV | 26:889 | Novel |
| 135 | No | Paired | 240370752 | T>A | *FMN2* | Exonic syn SNV | 33:882 | Novel |
| 139 | No | Paired | 240370752 | T>A | *FMN2* | Exonic syn SNV | 35:920 | Novel |
| 143 | No | Panel | 156639448 | T>C | *NES* | Exonic n-syn SNV | 44:895 | Novel |
| 143 | No | Panel | 156640308 | C>T | *NES* | Exonic syn SNV | 490:422 | rs3748571 |
| 143 | No | Panel | 156640678 | G>A | *NES* | Exonic n-syn SNV | 862:0 | rs2886443 |
| 143 | No | Panel | 226573274 | G>A | *PARP1* | Exonic syn SNV | 302:559 | rs61750984 |
| 143 | No | Panel | 226595754 | A>C | *PARP1* | UTR5 | 52:257 | Novel |
| 146 | No | Paired | 226574111 | G>T | *PARP1* | Exonic n-syn SNV | 11:187 | Novel |
| 146 | No | Paired | 240370717 | T>A | *FMN2* | Exonic n-syn SNV | 4:17 | Novel |
| 148 | No | Paired | 156641502 | T>C | *NES* | Exonic syn SNV | 45:827 | Novel |
| 148 | No | Paired | 156641537 | C>T^d^ | *NES* | Exonic n-syn SNV | 325:513 | rs951781 |
| 148 | No | Paired | 226595647 | C>G | *PARP1* | UTR5 | 69:30 | rs907187 |
| 148 | No | Paired | 240370752 | T>A | *FMN2* | Exonic syn SNV | 40:832 | Novel |
| 148 | No | Paired | 240370752 | T>A | *FMN2* | Exonic syn SNV | 30:739 | Novel |
| 149 | No | Paired | 152777964 | G>A | *LCE1C* | UTR5 | 53:113 | rs17624493 |
| 149 | No | Paired | 156640678 | G>A^d^ | *NES* | Exonic n-syn SNV | 425:304 | rs2886443 |
| 149 | No | Paired | 226570767 | G>A^d^ | *PARP1* | Exonic n-syn SNV | 542:426 | rs2230484 |
| 149 | No | Paired | 226580021 | G>T | *PARP1* | Intronic | 228:315 | rs1805405 |
| 150 | No | Paired | 240370752 | T>A | *FMN2* | Exonic syn SNV | 31:909 | Novel |
| 151 | No | Paired | 240370752 | T>A | *FMN2* | Exonic syn SNV | 33:928 | Novel |
| 153 | No | Panel | 152777908 | C>T | *LCE1C* | Exonic n-syn SNV | 75:74 | rs2006940 |
| 153 | No | Panel | 226552733 | A>C | *PARP1* | Exonic syn SNV | 187:721 | NA |
| 153 | No | Panel | 226595647 | C>G | *PARP1* | UTR5 | 81:46 | rs907187 |
| 153 | No | Panel | 226595754 | A>C | *PARP1* | UTR5 | 29:96 | Novel |
| 153 | No | Panel | 240256822 | C>A | *FMN2* | Exonic syn SNV | 392:400 | rs10926124 |
| 153 | No | Panel | 240341268 | A>G | *FMN2* | Exonic syn SNV | 169:729 | rs3765588 |
| 153 | No | Panel | 240492734 | G>A | *FMN2* | Exonic n-syn SNV | 725:204 | rs3795677 |
| 154 | No | Paired | 240370752 | T>A | *FMN2* | Exonic syn SNV | 29:933 | Novel |
| 155 | No | Panel | 156639300 | A>T | *NES* | Exonic syn SNV | 80:863 | Novel |
| 155 | No | Panel | 156639472 | T>C | *NES* | Exonic n-syn SNV | 44:831 | Novel |
| 155 | No | Panel | 156639636 | G>A | *NES* | Exonic syn SNV | 487:374 | rs11549292 |
| 155 | No | Panel | 156639732 | T>A | *NES* | Exonic n-syn SNV | 36:104 | Novel |
| 155 | No | Panel | 156640100 | T>C | *NES* | Exonic n-syn SNV | 66:720 | Novel |
| 155 | No | Panel | 156640520 | A>T | *NES* | Exonic n-syn SNV | 129:810 | Novel |
| 155 | No | Panel | 156640536 | A>T | *NES* | Exonic syn SNV | 96:839 | Novel |
| 155 | No | Panel | 226555302 | A>G | *PARP1* | Exonic n-syn SNV | 444:433 | rs1136410 |
| 155 | No | Panel | 226595647 | C>G | *PARP1* | UTR5 | 181:93 | rs907187 |
| 157 | No | Panel | 152777908 | C>T | *LCE1C* | Exonic n-syn SNV | 14:23 | rs2006940 |
| 157 | No | Panel | 156641537 | C>T | *NES* | Exonic n-syn SNV | 848:4 | rs951781 |
| 157 | No | Panel | 226567449 | G>A | *PARP1* | Intronic | 358:361 | rs369338473 |
| 157 | No | Panel | 240256822 | C>A | *FMN2* | Exonic syn SNV | 386:359 | rs10926124 |
| 157 | No | Panel | 240341258 | C>A | *FMN2* | Exonic n-syn SNV | 159:766 | NA |
| 157 | No | Panel | 240370634 | A>G | *FMN2* | Exonic n-syn SNV | 323:392 | Novel |
| 157 | No | Panel | 240601397 | A>T | *FMN2* | Exonic syn SNV | 81:890 | Novel |
| 158 | No | Paired | 240370752 | T>A | *FMN2* | Exonic syn SNV | 35:919 | Novel |
| 159 | No | Paired | 240255048 | T>C | *FMN2* | Upstream | 104:651 | Novel |

Abbreviations: BCP ALL, B-cell precursor acute lymphoblastic leukemia; dbSNP, data base of single nucleotide polymorphisms; n-syn, non-synonymous; Nt, nucleotide; ref, reference; rs-ID, reference SNP-ID; SNV, single nucleotide variant; syn, synonymous; var, variant. ^a^The reference used in the mutation detection analysis (Mutect) was based on paired remission samples or a reference panel created using the available 79 remission samples. ^b^Chromosome 1 positions according to the GRCh37 genome build. ^c^The dbSNP detection was based on build 138. ^d^These variants were verified by Sanger sequencing.
