# Supplementary material for: Mutation, methylation, and gene expression profiles in dup(1q)-positive pediatric B-cell precursor acute lymphoblastic leukemia
Source: Leukemia. 2018 Mar 12;32(10):2117–25. doi: 10.1038/s41375-018-0092-2 (PMC6170391; doi:10.1038/s41375-018-0092-2)
Supplement: Supplementary file 7 — Supplementary Table 7(DOCX 31 kb) [file 41375_2018_92_MOESM7_ESM.docx]

**Supplementary Table 7.** Deregulated genes in high hyperdiploid pediatric B-cell precursor acute lymphoblastic leukemia with 1q gain

| *Gene symbol* | *Gene name* | *Locus* | *Up or down* | *P* | *Fold* |
| --- | --- | --- | --- | --- | --- |
|  |  |  | *regulated* | *value* | *change* |
| *SNORD81* | small nucleolar RNA, C/D box 81 | 1q25.1 | Up | <0.01 | 14.1 |
| *SNORD78* | small nucleolar RNA, C/D box 78 | 1q25.1 | Up | <0.01 | 11.8 |
| *SNORD38A* | small nucleolar RNA, C/D box 38A | 1p34.1 | Up | <0.01 | 10.6 |
| *MIR3687* | microRNA 3687-1 | 21p11.2 | Up | <0.01 | 10.4 |
| *KCNIP1* | potassium voltage-gated channel interacting protein 1 | 5q35.1 | Up | <0.001 | 9.4 |
| *MIR186* | microRNA 186 | 1p31.1 | Up | <0.01 | 9.2 |
| *SNORD5* | small nucleolar RNA, C/D box 5 | 11q21 | Up | <0.01 | 9.0 |
| *SNORD48* | small nucleolar RNA, C/D box 48 | 6p21.33 | Up | <0.01 | 9.0 |
| *MIR130B* | microRNA 130b | 22q11.21 | Up | <0.01 | 8.5 |
| *MIR647* | microRNA 647 | 20q13.33 | Up | <0.01 | 8.4 |
| *SNORD119* | small nucleolar RNA, C/D box 119 | 20p13 | Up | <0.01 | 8.4 |
| *MIR4745* | microRNA 4745 | 19p13.3 | Up | <0.01 | 7.4 |
| *MIR3620* | microRNA 3620 | 1q42.13 | Up | <0.01 | 7.2 |
| *GPR75-ASB3* | GPR75-ASB3 readthrough | 2p16.2 | Up | <0.01 | 7.1 |
| *SNORD9* | small nucleolar RNA, C/D box 9 | 14q11.2 | Up | <0.01 | 6.8 |
| *RTL3* | retrotransposon Gag like 3 | Xq21.1 | Up | <0.01 | 6.4 |
| *CTAG2* | cancer/testis antigen 2 | Xq28 | Up | <0.0001 | 6.1 |
| *OR1J1* | olfactory receptor family 1 subfamily J member 1 | 9q33.2 | Up | <0.0001 | 6.0 |
| *CDHR3* | cadherin related family member 3 | 7q22.3 | Up | <0.01 | 5.4 |
| *ANKRD34C-AS1* | ANKRD34C antisense RNA 1 | 15q25.1 | Up | <0.01 | 5.2 |
| *CEACAM22P* | carcinoembryonic antigen related cell adhesion molecule 22, pseudogene | 19q13.31 | Up | <0.0001 | 4.5 |
| *DPH3P1* | diphthamide biosynthesis 3 pseudogene 1 | 20q13.33 | Up | <0.01 | 4.2 |
| *CYP2W1* | cytochrome P450 family 2 subfamily W member 1 | 7p22.3 | Up | <0.01 | 4.2 |
| *MYOT* | myotilin | 5q31.2 | Up | <0.01 | 3.9 |
| *TBX10* | T-box 10 | 11q13.2 | Up | <0.01 | 3.7 |
| *ANKRD45* | ankyrin repeat domain 45 | 1q25.1 | Up | <0.01 | 3.5 |
| *LEP* | leptin | 7q32.1 | Up | <0.01 | 3.5 |
| *HFE2* | hemochromatosis type 2 (juvenile) | 1q21.1 | Up | <0.001 | 3.3 |
| *SMPX* | small muscle protein, X-linked | Xp22.12 | Up | <0.01 | 3.2 |
| *HILS1* | histone linker H1 domain, spermatid-specific 1 (pseudogene) | 17q21.33 | Up | <0.01 | 3.1 |
| *SPDYC* | speedy/RINGO cell cycle regulator family member C | 11q13.1 | Up | <0.01 | 2.9 |
| *STK32A* | serine/threonine kinase 32A | 5q32 | Up | <0.0001 | 2.9 |
| *GTF2A1L* | general transcription factor IIA subunit 1 like | 2p16.3 | Up | <0.01 | 2.9 |
| *TCFL5* | transcription factor like 5 | 20q13.33 | Up | <0.01 | 2.9 |
| *SNORA70G* | small nucleolar RNA, H/ACA box 70G | 12q14 | Up | <0.01 | 2.9 |
| *CTAG1B* | cancer/testis antigen 1B | Xq28 | Up | <0.01 | 2.9 |
| *CYB561* | cytochrome b561 | 17q23.3 | Up | <0.01 | 2.8 |
| *SPATA12* | spermatogenesis associated 12 | 3p14.3 | Up | <0.01 | 2.8 |
| *PDC* | phosducin | 1q31.1 | Up | <0.001 | 2.8 |
| *CSF2* | colony stimulating factor 2 | 5q31.1 | Up | <0.01 | 2.6 |
| *SLC39A12* | solute carrier family 39 member 12 | 10p12.33 | Up | <0.01 | 2.6 |
| *HOXD-AS2* | HOXD cluster antisense RNA 2 | 2q31.1 | Up | <0.01 | 2.6 |
| *RAB13* | RAB13, member RAS oncogene family | 1q21.3 | Up | <0.01 | 2.6 |
| *NME5* | NME/NM23 family member 5 | 5q31.2 | Up | <0.01 | 2.6 |
| *TRIM31* | tripartite motif containing 31 | 6p22.1 | Up | <0.01 | 2.5 |
| *CSMD3* | CUB and Sushi multiple domains 3 | 8q23.3 | Up | <0.001 | 2.4 |
| *CDKN2A-AS1* | CDKN2A antisense RNA 1 (head to head) | 9p21.3 | Up | <0.01 | 2.4 |
| *GAS5* | growth arrest specific 5 (non-protein coding) | 1q25.1 | Up | <0.001 | 2.4 |
| *FATE1* | fetal and adult testis expressed 1 | Xq28 | Up | <0.01 | 2.3 |
| *C1orf194* | chromosome 1 open reading frame 194 | 1p13.3 | Up | <0.01 | 2.2 |
| *GSTA4* | glutathione S-transferase alpha 4 | 6p12.2 | Up | <0.01 | 2.2 |
| *SLC9A11* | solute carrier family 9 member C2 (putative) | 1q25.1 | Up | <0.01 | 2.2 |
| *CYP2F1* | cytochrome P450 family 2 subfamily F member 1 | 19q13.2 | Up | <0.0001 | 2.2 |
| *LOC646813* | DExH-box helicase 9 pseudogene | 11p11.12 | Up | <0.01 | 2.1 |
| *OVCH1* | ovochymase 1 | 12p11.22 | Up | <0.01 | 2.1 |
| *LINC00473* | long intergenic non-protein coding RNA 473 | 6q27 | Up | <0.001 | 2.0 |
| *RABGAP1L* | RAB GTPase activating protein 1 like | 1q25.1 | Up | <0.001 | 2.0 |
| *OR1J4* | olfactory receptor family 1 subfamily J member 4 | 9q33.2 | Up | <0.01 | 2.0 |
| *SNRPE* | small nuclear ribonucleoprotein polypeptide E | 1q32.1 | Up | <0.01 | 2.0 |
| *C11orf41* | KIAA1549 like | 11p13 | Up | <0.01 | 2.0 |
| *LOC100128993* | uncharacterized LOC100128993 | 8p21.3-22 | Up | <0.01 | 2.0 |
| *DGKB* | diacylglycerol kinase beta | 7p21.2 | Up | <0.01 | 2.0 |
| *APELA* | apelin receptor early endogenous ligand | 4q32.3 | Up | <0.001 | 2.0 |
| *LINC01405* | long intergenic non-protein coding RNA 1405 | 12q24.11 | Up | <0.01 | 1.9 |
| *NENF* | neudesin neurotrophic factor | 1q32.3 | Up | <0.01 | 1.9 |
| *PPA1* | pyrophosphatase (inorganic) 1 | 10q22.1 | Up | <0.01 | 1.9 |
| *KIF9* | kinesin family member 9 | 3p21.31 | Up | <0.01 | 1.9 |
| *NPM3* | nucleophosmin/nucleoplasmin 3 | 10q24.32 | Up | <0.01 | 1.9 |
| *LINC00908* | long intergenic non-protein coding RNA 908 | 18q23 | Up | <0.001 | 1.9 |
| *RIMS2* | regulating synaptic membrane exocytosis 2 | 8q22.3 | Up | <0.01 | 1.9 |
| *ADAM15* | ADAM metallopeptidase domain 15 | 1q21.3 | Up | <0.01 | 1.9 |
| *DMRT1* | doublesex and mab-3 related transcription factor 1 | 9p24.3 | Up | <0.001 | 1.9 |
| *TAAR1* | trace amine associated receptor 1 | 6q23.2 | Up | <0.001 | 1.8 |
| *SFXN4* | sideroflexin 4 | 10q26.11 | Up | <0.01 | 1.8 |
| *DPM3* | dolichyl-phosphate mannosyltransferase subunit 3 | 1q22 | Up | <0.01 | 1.8 |
| *PLEKHO1* | pleckstrin homology domain containing O1 | 1q21.2 | Up | <0.01 | 1.8 |
| *SLC39A1* | solute carrier family 39 member 1 | 1q21.3 | Up | <0.01 | 1.8 |
| *OACYLP* | O-acyltransferase like, pseudogene | 18q21.32 | Up | <0.01 | 1.8 |
| *MCHR2-AS1* | MCHR2 antisense RNA 1 | 6q16.2-16.3 | Up | <0.01 | 1.7 |
| *ASH1L-AS1* | ASH1L antisense RNA 1 | 1q22 | Up | <0.001 | 1.7 |
| *C1orf43* | chromosome 1 open reading frame 43 | 1q21.3 | Up | <0.0001 | 1.7 |
| *LGALS16* | galectin 16 | 19q13.2 | Up | <0.01 | 1.7 |
| *FABP7* | fatty acid binding protein 7 | 6q22.31 | Up | <0.01 | 1.7 |
| *UFC1* | ubiquitin-fold modifier conjugating enzyme 1 | 1q23.3 | Up | <0.001 | 1.7 |
| *SMYD3* | SET and MYND domain containing 3 | 1q44 | Up | <0.01 | 1.7 |
| *IGDCC3* | immunoglobulin superfamily DCC subclass member 3 | 15q22.31 | Up | <0.001 | 1.7 |
| *LOC152578* | long intergenic non-protein coding RNA 1618 | 4q12 | Up | <0.01 | 1.7 |
| *PRDX6* | peroxiredoxin 6 | 1q25.1 | Up | <0.001 | 1.7 |
| *SFTA1P* | surfactant associated 1, pseudogene | 10p14 | Up | <0.01 | 1.6 |
| *ELK4* | ELK4, ETS transcription factor | 1q32.1 | Up | <0.01 | 1.6 |
| *ACBD6* | acyl-CoA binding domain containing 6 | 1q25.2-25.3 | Up | <0.001 | 1.6 |
| *HSPB3* | heat shock protein family B (small) member 3 | 5q11.2 | Up | <0.01 | 1.6 |
| *SERPINB3* | serpin family B member 3 | 18q21.33 | Up | <0.01 | 1.6 |
| *FAM90A2P* | family with sequence similarity 90 member A2, pseudogene | 8p23.1 | Up | <0.01 | 1.6 |
| *C9orf153* | chromosome 9 open reading frame 153 | 9q21.33 | Up | <0.01 | 1.6 |
| *GALP* | galanin like peptide | 19q13.43 | Up | <0.01 | 1.6 |
| *LINC00229* | long intergenic non-protein coding RNA 229 | 22q13.3 | Up | <0.01 | 1.6 |
| *TEX46* | testis expressed 46 | 1p36.12 | Up | <0.01 | 1.6 |
| *SCAMP3* | secretory carrier membrane protein 3 | 1q22 | Up | <0.01 | 1.6 |
| *DAP3* | death associated protein 3 | 1q22 | Up | <0.001 | 1.6 |
| *TAS2R38* | taste 2 receptor member 38 | 7q34 | Up | <0.01 | 1.6 |
| *PIP5K1A* | phosphatidylinositol-4-phosphate 5-kinase type 1 alpha | 1q21.3 | Up | <0.01 | 1.6 |
| *LINC00710* | long intergenic non-protein coding RNA 710 | 10p14 | Up | <0.01 | 1.6 |
| *OR7G3* | olfactory receptor family 7 subfamily G member 3 | 19p13.2 | Up | <0.01 | 1.6 |
| *POLR3C* | RNA polymerase III subunit C | 1q21.1 | Up | <0.001 | 1.6 |
| *VENTXP7* | VENT homeobox pseudogene 7 | 3p24.3 | Up | <0.01 | 1.6 |
| *EIF2D* | eukaryotic translation initiation factor 2D | 1q32.1 | Up | <0.01 | 1.5 |
| *PIPSL* | PIP5K1A and PSMD4-like, pseudogene | 10q23.33 | Up | <0.01 | 1.5 |
| *KRTCAP2* | keratinocyte associated protein 2 | 1q22 | Up | <0.01 | 1.5 |
| *OR13C5* | olfactory receptor family 13 subfamily C member 5 | 9q31.1 | Up | <0.01 | 1.5 |
| *PSMD4* | proteasome 26S subunit, non-ATPase 4 | 1q21.3 | Up | <0.01 | 1.5 |
| *C1orf105* | chromosome 1 open reading frame 105 | 1q24.3 | Up | <0.01 | 1.5 |
| *OR1N2* | olfactory receptor family 1 subfamily N member 2 | 9q33.2 | Up | <0.01 | 1.5 |
| *PPIAP46* | peptidylprolyl isomerase A pseudogene 46 | 15q24.1 | Up | <0.01 | 1.5 |
| *PFDN2* | prefoldin subunit 2 | 1q23.3 | Up | <0.01 | 1.5 |
| *ADAR* | adenosine deaminase, RNA specific | 1q21.3 | Up | <0.01 | 1.5 |
| *WNT2* | Wnt family member 2 | 7q31.2 | Up | <0.01 | 1.5 |
| *CCL14-CCL15* | CCL15-CCL14 readthrough (NMD candidate) | 17q12 | Up | <0.01 | 1.5 |
| *KRTAP10-6* | keratin associated protein 10-6 | 21q22.3 | Up | <0.01 | 1.5 |
| *OR52N5* | olfactory receptor family 52 subfamily N member 5 | 11p15.4 | Up | <0.01 | 1.5 |
| *LINC00421* | long intergenic non-protein coding RNA 421 | 13q12.11 | Up | <0.01 | 1.5 |
| *CERS2* | ceramide synthase 2 | 1q21.3 | Up | <0.01 | 1.5 |
| *PSMB4* | proteasome subunit beta 4 | 1q21.3 | Up | <0.01 | 1.5 |
| *DCAF8L1* | DDB1 and CUL4 associated factor 8 like 1 | Xp21.3 | Up | <0.001 | 1.5 |
| *JTB* | jumping translocation breakpoint | 1q21.3 | Up | <0.01 | 1.5 |
| *OR7E91P* | olfactory receptor family 7 subfamily E member 91 pseudogene | 2p13.3 | Up | <0.01 | 1.5 |
| *CDC123* | cell division cycle 123 | 10p13-14 | Up | <0.01 | 1.5 |
| *LINC00272* | long intergenic non-protein coding RNA 272 | 1q25.3 | Up | <0.01 | 1.5 |
| *TMEM61* | transmembrane protein 61 | 1p32.3 | Up | <0.01 | 1.5 |
| *ANXA7* | annexin A7 | 10q22.2 | Up | <0.01 | 1.5 |
| *MRPL9* | mitochondrial ribosomal protein L9 | 1q21.3 | Up | <0.01 | 1.4 |
| *SPRR1A* | small proline rich protein 1A | 1q21.3 | Up | <0.01 | 1.4 |
| *CLDN24* | claudin 24 | 4q35.1 | Up | <0.01 | 1.4 |
| *LINC02372* | long intergenic non-protein coding RNA 2372 | 12q24.32 | Up | <0.01 | 1.4 |
| *LHFPL3-AS1* | LHFPL3 antisense RNA 1 | 7q22.2 | Up | <0.01 | 1.4 |
| *CXCL17* | C-X-C motif chemokine ligand 17 | 19q13.2 | Up | <0.01 | 1.4 |
| *LHX9* | LIM homeobox 9 | 1q31.3 | Up | <0.01 | 1.4 |
| *MAPT-IT1* | keratin associated protein 10-6 | 17q21.31 | Up | <0.01 | 1.4 |
| *TRIM64B* | tripartite motif containing 64B | 11q14.3 | Up | <0.01 | 1.4 |
| *RFWD2* | ring finger and WD repeat domain 2 | 1q25.1-25.2 | Up | <0.01 | 1.4 |
| *UBE2Q1* | ubiquitin conjugating enzyme E2 Q1 | 1q21.3 | Up | <0.001 | 1.4 |
| *SETDB1* | SET domain bifurcated 1 | 1q21.3 | Up | <0.01 | 1.4 |
| *C12orf50* | chromosome 12 open reading frame 50 | 12q21.32 | Up | <0.01 | 1.4 |
| *OR51B4* | olfactory receptor family 51 subfamily B member 4 | 11p15.4 | Up | <0.01 | 1.4 |
| *HMGB4* | high mobility group box 4 | 1p35.1 | Up | <0.01 | 1.4 |
| *PRAMEF12* | PRAME family member 12 | 1p36.21 | Up | <0.01 | 1.4 |
| *OR2T27* | olfactory receptor family 2 subfamily T member 27 | 1q44 | Up | <0.01 | 1.4 |
| *ESRRG* | estrogen related receptor gamma | 1q41 | Up | <0.01 | 1.4 |
| *DCAF8L2* | DDB1 and CUL4 associated factor 8 like 2 | Xp21.3 | Up | <0.01 | 1.4 |
| *SATL1* | spermidine/spermine N1-acetyl transferase like 1 | Xq21.1 | Up | <0.01 | 1.4 |
| *C4orf51* | chromosome 4 open reading frame 51 | 4q31.21 | Up | <0.01 | 1.4 |
| *NOL4L-DT* | NOL4L divergent transcript | 20q11.21 | Up | <0.01 | 1.4 |
| *POTEA* | POTE ankyrin domain family member A | 8p11.1 | Up | <0.01 | 1.4 |
| *CYP4B1* | cytochrome P450 family 4 subfamily B member 1 | 1p33 | Up | <0.01 | 1.4 |
| *DMRT3* | doublesex and mab-3 related transcription factor 3 | 9p24.3 | Up | <0.01 | 1.4 |
| *TAAR3* | trace amine associated receptor 3, pseudogene | 6q23.2 | Up | <0.01 | 1.4 |
| *NCR2* | natural cytotoxicity triggering receptor 2 | 6p21.1 | Up | <0.01 | 1.4 |
| *LINC00298* | long intergenic non-protein coding RNA 298 | 2p25.1 | Up | <0.01 | 1.4 |
| *OR13C8* | olfactory receptor family 13 subfamily C member 8 | 9q31.1 | Up | <0.01 | 1.4 |
| *SLC26A3* | solute carrier family 26 member 3 | 7q22.3-31.1 | Up | <0.01 | 1.4 |
| *VAX2* | ventral anterior homeobox 2 | 2p13.3 | Up | <0.01 | 1.3 |
| *SLC15A5* | solute carrier family 15 member 5 | 12p12.3 | Up | <0.01 | 1.3 |
| *KRTAP9-8* | keratin associated protein 9-8 | 17q21.2 | Up | <0.01 | 1.3 |
| *LINC01532* | long intergenic non-protein coding RNA 1532 | 19q12 | Up | <0.01 | 1.3 |
| *ALPPL2* | alkaline phosphatase, placental like 2 | 2q37.1 | Up | <0.01 | 1.3 |
| *LINC00857* | long intergenic non-protein coding RNA 857 | 10q22.3 | Up | <0.01 | 1.3 |
| *TBC1D21* | TBC1 domain family member 21 | 15q24.1 | Up | <0.01 | 1.3 |
| *KRT17P5* | keratin 17 pseudogene 5 | 17p11.2 | Up | <0.01 | 1.3 |
| *PRSS38* | protease, serine 38 | 1q42.13 | Up | <0.01 | 1.3 |
| *PRDM9* | PRDM9 | 5p14.2 | Up | <0.01 | 1.3 |
| *RBM46* | RNA binding motif protein 46 | 4q32.1 | Up | <0.01 | 1.3 |
| *GABRB1* | gamma-aminobutyric acid type A receptor beta1 subunit | 4p12 | Up | <0.01 | 1.3 |
| *TTPA* | alpha tocopherol transfer protein | 8q12.3 | Up | <0.01 | 1.3 |
| *CSTT* | cystatin 13, pseudogene | 20p11.21 | Up | <0.01 | 1.3 |
| *ZNF735* | zinc finger protein 735 | 7q11.21 | Up | <0.01 | 1.3 |
| *PROP1* | PROP paired-like homeobox 1 | 5q35.3 | Up | <0.01 | 1.3 |
| *LINC00992* | long intergenic non-protein coding RNA 992 | 5q23.1 | Up | <0.01 | 1.3 |
| *PTF1A* | pancreas specific transcription factor, 1a | 10p12.2 | Up | <0.01 | 1.3 |
| *KCNIP4-IT1* | KCNIP4 intronic transcript 1 | 4p15.2 | Up | <0.001 | 1.3 |
| *AADACL4* | arylacetamide deacetylase like 4 | 1p36.21 | Up | <0.01 | 1.2 |
| *HTR3E* | 5-hydroxytryptamine receptor 3E | 3q27.1 | Up | <0.01 | 1.2 |
| *FLJ36000* | uncharacterized FLJ36000 | 17p11.2 | Up | <0.01 | 1.2 |
| *NLRP4* | NLR family pyrin domain containing 4 | 19q13.43 | Up | <0.01 | 1.2 |
| *FAM155B* | family with sequence similarity 155 member B | Xq13.1 | Up | <0.01 | 1.2 |
| *KCNA4* | potassium voltage-gated channel subfamily A member 4 | 11p14.1 | Up | <0.01 | 1.2 |
| *CHRNB3* | cholinergic receptor nicotinic beta 3 subunit | 8p11.21 | Up | <0.01 | 1.2 |
| *LINC02346* | long intergenic non-protein coding RNA 2346 | 15q12 | Up | <0.01 | 1.2 |
| *UGT1A3* | UDP glucuronosyltransferase family 1 member A3 | 2q37.1 | Up | <0.01 | 1.2 |
| *UGT1A1* | UDP glucuronosyltransferase family 1 member A1 | 2q37.1 | Up | <0.01 | 1.2 |
| *TCF24* | transcription factor 24 | 8q13.1 | Up | <0.01 | 1.2 |
| *CCDC83* | coiled-coil domain containing 83 | 11q14.1-14.2 | Up | <0.01 | 1.2 |
| *LINC01630* | long intergenic non-protein coding RNA 1630 | 18q21.2 | Up | <0.01 | 1.1 |
| *PRR23C* | proline rich 23C | 3q23 | Up | <0.01 | 1.1 |
| *LOC400940* | uncharacterized LOC400940 | 2p25.2 | Up | <0.01 | 1.1 |
| *ZIC4* | Zic family member 4 | 3q24 | Up | <0.01 | 1.1 |
| *PKD1L2* | polycystin 1 like 2 (gene/pseudogene) | 16q23.2 | Up | <0.01 | 1.0 |
| *ZNF527* | zinc finger protein 527 | 19q13.12 | Down | <0.01 | 0.7 |
| *CABLES2* | Cdk5 and Abl enzyme substrate 2 | 20q13.33 | Down | <0.01 | 0.7 |
| *CCNG2* | cyclin G2 | 4q21.1 | Down | <0.01 | 0.6 |
| *FLJ39534* | KIF9 antisense RNA 1 | 3p21.31 | Down | <0.01 | 0.6 |
| *ANKHD1-EIF4EBP3* | ANKHD1-EIF4EBP3 readthrough | 5q31.3 | Down | <0.01 | 0.5 |
| *DOCK10* | dedicator of cytokinesis 10 | 2q36.2 | Down | <0.01 | 0.5 |
| *LBH* | limb bud and heart development | 2p23.1 | Down | <0.01 | 0.5 |
| *BBS4* | Bardet-Biedl syndrome 4 | 15q24.1 | Down | <0.01 | 0.5 |
| *ACTR5* | ACTR5 | 20q11.23 | Down | <0.01 | 0.5 |
| *CAMK2D* | calcium/calmodulin dependent protein kinase II delta | 4q26 | Down | <0.01 | 0.5 |
| *CCDC96* | coiled-coil domain containing 96 | 4p16.1 | Down | <0.01 | 0.5 |
| *SMTN* | smoothelin | 22q12.2 | Down | <0.01 | 0.4 |
| *LOXL4* | lysyl oxidase like 4 | 10q24.2 | Down | <0.01 | 0.4 |
| *POU2AF1* | POU class 2 associating factor 1 | 11q23.1 | Down | <0.01 | 0.4 |
| *GSPT2* | G1 to S phase transition 2 | Xp11.22 | Down | <0.01 | 0.4 |
| *GPR68* | G protein-coupled receptor 68 | 14q32.11 | Down | <0.01 | 0.4 |
| *C11orf82* | DNA damage induced apoptosis suppressor | 11q14.1 | Down | <0.01 | 0.4 |
| *ZNF853* | zinc finger protein 853 | 7p22.1 | Down | <0.01 | 0.4 |
| *RDH13* | retinol dehydrogenase 13 | 19q13.42 | Down | <0.01 | 0.3 |
| *ACACB* | acetyl-CoA carboxylase beta | 12q24.11 | Down | <0.01 | 0.3 |
| *APBB1* | amyloid beta precursor protein binding family B member 1 | 11p15.4 | Down | <0.01 | 0.3 |
| *ATG9B* | autophagy related 9B | 7q36.1 | Down | <0.01 | 0.3 |
| *MAPT* | microtubule associated protein tau | 17q21.31 | Down | <0.01 | 0.3 |
| *MEI1* | meiotic double-stranded break formation protein 1 | 22q13.2 | Down | <0.01 | 0.3 |
| *PYHIN1* | pyrin and HIN domain family member 1 | 1q23.1 | Down | <0.001 | 0.3 |
| *LOC389641* | uncharacterized LOC389641 | 8p21.3 | Down | <0.001 | 0.3 |
| *ZNF831* | zinc finger protein 831 | 20q13.32 | Down | <0.01 | 0.3 |
| *TMEM30B* | transmembrane protein 30B | 14q23.1 | Down | <0.01 | 0.3 |
| *MIOX* | myo-inositol oxygenase | 22q13.33 | Down | <0.01 | 0.3 |
| *SPAG4* | sperm associated antigen 4 | 20q11.22 | Down | <0.01 | 0.3 |
| *PGM5* | phosphoglucomutase 5 | 9q21.11 | Down | <0.01 | 0.3 |
| *DYNC2H1* | dynein cytoplasmic 2 heavy chain 1 | 11q22.3 | Down | <0.01 | 0.2 |
| *LMTK3* | lemur tyrosine kinase 3 | 19q13.33 | Down | <0.0001 | 0.2 |
| *FSTL4* | follistatin like 4 | 5q31.1 | Down | <0.01 | 0.2 |
| *SHISA3* | shisa family member 3 | 4p13 | Down | <0.01 | 0.2 |
| *JAG2* | jagged 2 | 14q32.33 | Down | <0.01 | 0.2 |
| *BAI2* | adhesion G protein-coupled receptor B2 | 1p35.2 | Down | <0.001 | 0.2 |
| *FKBP9L* | FK506 binding protein 9 pseudogene 1 | 7p11.2 | Down | <0.01 | 0.2 |
| *TUBB4A* | tubulin beta 4A class Iva | 19p13.3 | Down | <0.01 | 0.2 |
| *TMEM63C* | transmembrane protein 63C | 14q24.3 | Down | <0.0001 | 0.2 |
| *TRIM47* | tripartite motif containing 47 | 17q25 | Down | <0.01 | 0.2 |
| *FKBP9* | FK506 binding protein 9 | 7p14.3 | Down | <0.01 | 0.2 |
| *TGM5* | transglutaminase 5 | 15q15.2 | Down | <0.01 | 0.2 |
| *GPR162* | G protein-coupled receptor 162 | 12p13 | Down | <0.01 | 0.2 |
| *PPM1N* | protein phosphatase, Mg2+/Mn2+ dependent 1N (putative) | 19q13.32 | Down | <0.0001 | 0.2 |
| *FBLN2* | fibulin 2 | 3p25.1 | Down | <0.01 | 0.1 |
| *OR1F2P* | olfactory receptor family 1 subfamily F member 2 pseudogene | 16p13.3 | Down | <0.01 | 0.1 |
| *CAV1* | caveolin 1 | 7q31.2 | Down | <0.01 | 0.1 |
| *ARHGAP32* | Rho GTPase activating protein 32 | 11q24.3 | Down | <0.01 | 0.1 |
| *PRL* | prolactin | 6p22.3 | Down | <0.01 | 0.1 |
| *KCNQ2* | potassium voltage-gated channel subfamily Q member 2 | 20q13.33 | Down | <0.01 | 0.1 |
| *LINC00544* | long intergenic non-protein coding RNA 544 | 13q12.3 | Down | <0.01 | 0.1 |
| *BMP3* | bone morphogenetic protein 3 | 4q21.21 | Down | <0.01 | 0.1 |
| *DFNA5* | gasdermin E | 7p15.3 | Down | <0.01 | 0.1 |
| *AKAP12* | A-kinase anchoring protein 12 | 6q25.1 | Down | <0.001 | 0.1 |
| *TPTEP1* | transmembrane phosphatase with tensin homology pseudogene 1 | 22q11.1 | Down | <0.001 | 0.1 |
