# Supplementary material for: Mutation, methylation, and gene expression profiles in dup(1q)-positive pediatric B-cell precursor acute lymphoblastic leukemia
Source: Leukemia. 2018 Mar 12;32(10):2117–25. doi: 10.1038/s41375-018-0092-2 (PMC6170391; doi:10.1038/s41375-018-0092-2)
Supplement: Supplementary file 8 — Supplementary Table 8(DOCX 44 kb) [file 41375_2018_92_MOESM8_ESM.docx]

**Supplementary Table 8.** Deregulated genes in t(1;19)(q23;p13)-positive pediatric B-cell precursor acute lymphoblastic leukemia with 1q gain

| *Gene symbol* | *Gene name* | *Locus* | *Up or down* | *P* | *Fold* |
| --- | --- | --- | --- | --- | --- |
|  |  |  | *regulated* | *value* | *change* |
| *SNORA62* | small nucleolar RNA, H/ACA box 62 | 3p22.1 | Up | <0.01 | 289.2 |
| *LY6H* | lymphocyte antigen 6 family member H | 8q24.3 | Up | <0.001 | 161.8 |
| *HAMP* | hepcidin antimicrobial peptide | 19q13.12 | Up | <0.01 | 61.4 |
| *PVALB* | parvalbumin | 22q12.3 | Up | <0.01 | 60.0 |
| *GALNT9* | polypeptide N-acetylgalactosaminyltransferase 9 | 12q24.33 | Up | <0.01 | 45.3 |
| *TNFRSF12A* | TNF receptor superfamily member 12A | 16p13.3 | Up | <0.01 | 35.1 |
| *ARHGAP29* | Rho GTPase activating protein 29 | 1p22.1 | Up | <0.01 | 32.4 |
| *MAP1B* | microtubule associated protein 1B | 5q13.2 | Up | <0.001 | 32.1 |
| *PDGFA* | platelet derived growth factor subunit A | 7p22.3 | Up | <0.01 | 25.6 |
| *DACT3-AS1* | DACT3 antisense RNA 1 | 19q13.32 | Up | <0.01 | 23.6 |
| *LMO1* | LIM domain only 1 | 11p15.4 | Up | <0.0001 | 22.2 |
| *KRTAP5-8* | keratin associated protein 5-8 | 11q13.4 | Up | <0.001 | 22.0 |
| *TMEM31* | transmembrane protein 31 | Xq22.2 | Up | <0.001 | 20.4 |
| *FAM150B* | ALK and LTK ligand 2 | 2p25.3 | Up | <0.01 | 19.6 |
| *GRB7* | growth factor receptor bound protein 7 | 17q12 | Up | <0.001 | 19.4 |
| *HR* | HR, lysine demethylase and nuclear receptor corepressor | 8p21.3 | Up | <0.001 | 19.3 |
| *TUBB3* | tubulin beta 3 class III | 16q24.3 | Up | <0.01 | 16.0 |
| *KRTAP5-9* | keratin associated protein 5-9 | 11q13.4 | Up | <0.01 | 16.0 |
| *OLFML2B* | olfactomedin like 2B | 1q23.3 | Up | <0.0001 | 15.7 |
| *PKP1* | plakophilin 1 | 1q32.1 | Up | <0.01 | 15.0 |
| *ASB9* | ankyrin repeat and SOCS box containing 9 | Xp22.2 | Up | <0.01 | 14.3 |
| *GJA4* | gap junction protein alpha 4 | 1p34.3 | Up | <0.01 | 14.0 |
| *TCTE3* | t-complex-associated-testis-expressed 3 | 6q27 | Up | <0.01 | 13.4 |
| *SAG* | S-antigen visual arrestin | 2q37.1 | Up | <0.01 | 12.5 |
| *ADORA1* | adenosine A1 receptor | 1q32.1 | Up | <0.01 | 11.8 |
| *EGFL7* | EGF like domain multiple 7 | 9q34.3 | Up | <0.001 | 11.7 |
| *AIF1L* | allograft inflammatory factor 1 like | 9q34.12-34.13 | Up | <0.01 | 11.3 |
| *FNBP1L* | formin binding protein 1 like | 1p22.1 | Up | <0.01 | 10.1 |
| *MYEF2* | myelin expression factor 2 | 15q21.1 | Up | <0.01 | 10.1 |
| *CAPN8* | calpain 8 | 1q41 | Up | <0.001 | 9.4 |
| *DIRAS1* | DIRAS family GTPase 1 | 19p13.3 | Up | <0.01 | 9.2 |
| *NCMAP* | non-compact myelin associated protein | 1p36.11 | Up | <0.01 | 8.9 |
| *LINC02145* | long intergenic non-protein coding RNA 2145 | 5p15.31 | Up | <0.0001 | 8.9 |
| *SP7* | Sp7 transcription factor | 12q13.13 | Up | <0.01 | 8.8 |
| *PGM5P2* | phosphoglucomutase 5 pseudogene 2 | 9p11.2 | Up | <0.01 | 8.6 |
| *QSOX2* | quiescin sulfhydryl oxidase 2 | 9q34.3 | Up | <0.01 | 8.4 |
| *PRMT8* | protein arginine methyltransferase 8 | 12p13.32 | Up | <0.01 | 8.1 |
| *OPALIN* | oligodendrocytic myelin paranodal and inner loop protein | 10q24.1 | Up | <0.01 | 8.0 |
| *RPA4* | replication protein A4 | Xq21.33 | Up | <0.01 | 7.9 |
| *ACPT* | acid phosphatase 4 | 19q13.33 | Up | <0.01 | 7.6 |
| *SYT5* | synaptotagmin 5 | 19q13.42 | Up | <0.01 | 7.5 |
| *GAD2* | glutamate decarboxylase 2 | 10p12.1 | Up | <0.01 | 7.3 |
| *GNG12* | G protein subunit gamma 12 | 1p31.3 | Up | <0.01 | 7.2 |
| *PRR15* | proline rich 15 | 7p14.3 | Up | <0.01 | 6.8 |
| *LINC00260* | long intergenic non-protein coding RNA 260 | 1q32.1 | Up | <0.01 | 6.8 |
| *GPR146* | G protein-coupled receptor 146 | 7p22.3 | Up | <0.01 | 6.8 |
| *SSTR2* | somatostatin receptor 2 | 17q25.1 | Up | <0.01 | 6.7 |
| *FAM110C* | family with sequence similarity 110 member C | 2p25.3 | Up | <0.001 | 6.7 |
| *C1orf54* | chromosome 1 open reading frame 54 | 1q21.2 | Up | <0.01 | 6.4 |
| *KCNH7* | potassium voltage-gated channel subfamily H member 7 | 2q24.2 | Up | <0.001 | 6.4 |
| *ELFN1* | extracellular leucine rich repeat and fibronectin type III domain containing 1 | 7p22.3 | Up | <0.01 | 5.9 |
| *WNT2B* | Wnt family member 2B | 1p13.2 | Up | <0.01 | 5.7 |
| *ECT2L* | epithelial cell transforming 2 like | 6q24.1 | Up | <0.01 | 5.7 |
| *C20orf197* | chromosome 20 open reading frame 197 | 20q13.33 | Up | <0.01 | 5.6 |
| *LINC01686* | long intergenic non-protein coding RNA 1686 | 1q25.3 | Up | <0.01 | 5.6 |
| *ECM1* | extracellular matrix protein 1 | 1q21.2 | Up | <0.01 | 5.4 |
| *OPRK1* | opioid receptor kappa 1 | 8q11.23 | Up | <0.01 | 5.3 |
| *CHRNG* | cholinergic receptor nicotinic gamma subunit | 2q37.1 | Up | <0.01 | 5.3 |
| *VASH2* | vasohibin 2 | 1q32.3 | Up | <0.01 | 5.1 |
| *C1orf27* | chromosome 1 open reading frame 27 | 1q31.1 | Up | <0.01 | 4.9 |
| *ZNF10* | zinc finger protein 10 | 12q24.33 | Up | <0.01 | 4.8 |
| *PLEKHG1* | pleckstrin homology and RhoGEF domain containing G1 | 6q25.1 | Up | <0.01 | 4.7 |
| *CD55* | CD55 molecule (Cromer blood group) | 1q32.2 | Up | <0.01 | 4.3 |
| *AHCTF1* | G protein-coupled receptor 146 | 1q44 | Up | <0.01 | 4.3 |
| *GNA15* | G protein subunit alpha 15 | 19p13.3 | Up | <0.01 | 4.2 |
| *ZHX1-C8ORF76* | ZHX1-C8orf76 readthrough | 8q24.13 | Up | <0.001 | 4.2 |
| *CSDA* | Y-box binding protein 3 | 12p13.2 | Up | <0.01 | 4.0 |
| *DUSP26* | dual specificity phosphatase 26 | 8p12 | Up | <0.01 | 4.0 |
| *CD109* | CD109 molecule | 6q13 | Up | <0.01 | 3.9 |
| *NEIL1* | nei like DNA glycosylase 1 | 15q24.2 | Up | <0.01 | 3.9 |
| *MYH15* | myosin heavy chain 15 | 3q13.13 | Up | <0.01 | 3.8 |
| *LRRC70* | leucine rich repeat containing 70 | 5q12.1 | Up | <0.01 | 3.8 |
| *ASPM* | abnormal spindle microtubule assembly | 1q31.3 | Up | <0.01 | 3.8 |
| *PTBP2* | polypyrimidine tract binding protein 2 | 1p21.3 | Up | <0.001 | 3.7 |
| *GORAB* | golgin, RAB6 interacting | 1q24.2 | Up | <0.001 | 3.6 |
| *RETREG1* | reticulophagy regulator 1 | 5p15.1 | Up | <0.01 | 3.6 |
| *C1orf101* | catsper channel auxiliary subunit epsilon | 1q44 | Up | <0.01 | 3.5 |
| *LCN6* | lipocalin 6 | 9q34.3 | Up | <0.01 | 3.5 |
| *NEK3* | NIMA related kinase 3 | 13q14.3 | Up | <0.001 | 3.5 |
| *PLXDC2* | plexin domain containing 2 | 10p12.31 | Up | <0.01 | 3.5 |
| *MUC1* | mucin 1, cell surface associated | 1q22 | Up | <0.01 | 3.4 |
| *CEP170* | centrosomal protein 170 | 1q43 | Up | <0.001 | 3.4 |
| *RGPD4* | RANBP2-like and GRIP domain containing 4 | 2q12.3 | Up | <0.01 | 3.3 |
| *ASGR1* | asialoglycoprotein receptor 1 | 17p13.1 | Up | <0.01 | 3.3 |
| *ABCA17P* | ATP binding cassette subfamily A member 17, pseudogene | 16p13.3 | Up | <0.01 | 3.3 |
| *C1orf96* | centriole, cilia and spindle associated protein | 1q42.13 | Up | <0.01 | 3.3 |
| *MAGI3* | membrane associated guanylate kinase, WW and PDZ domain containing 3 | 1p13.2 | Up | <0.01 | 3.3 |
| *ERO1LB* | endoplasmic reticulum oxidoreductase 1 beta | 1q42.3 | Up | <0.01 | 3.1 |
| *TOMM20* | translocase of outer mitochondrial membrane 20 | 1q42.3 | Up | <0.01 | 3.0 |
| *ZNF195* | zinc finger protein 195 | 11p15.5 | Up | <0.01 | 3.0 |
| *FRG2C* | FRG2C | 3p12.3 | Up | <0.01 | 2.9 |
| *RFPL3-AS1* | RFPL3 antisense | 22q12.3 | Up | <0.01 | 2.9 |
| *FRG2EP* | FSHD region gene 2 family member E, pseudogene | 20q11.1 | Up | <0.01 | 2.9 |
| *TCF4* | transcription factor 4 | 18q21.2 | Up | <0.01 | 2.9 |
| *CEP350* | centrosomal protein 350 | 1q25.2 | Up | <0.01 | 2.9 |
| *TFB2M* | transcription factor B2, mitochondrial | 1q44 | Up | <0.01 | 2.8 |
| *ABCC2* | ATP binding cassette subfamily C member 2 | 10q24.2 | Up | <0.01 | 2.8 |
| *TIMM17A* | translocase of inner mitochondrial membrane 17A | 1q32.1 | Up | <0.01 | 2.8 |
| *ELK4* | ELK4, ETS transcription factor | 1q32.1 | Up | <0.001 | 2.8 |
| *RC3H1* | ring finger and CCCH-type domains 1 | 1q25.1 | Up | <0.01 | 2.8 |
| *PPFIA4* | PTPRF interacting protein alpha 4 | 1q32.1 | Up | <0.01 | 2.8 |
| *CAPN3* | calpain 3 | 15q15.1 | Up | <0.01 | 2.8 |
| *RRP15* | ribosomal RNA processing 15 homolog | 1q41 | Up | <0.001 | 2.8 |
| *KCTD3* | potassium channel tetramerization domain containing 3 | 1q41 | Up | <0.001 | 2.7 |
| *FAM35B2* | FAM35DP | 10q11.22 | Up | <0.01 | 2.7 |
| *NRIP1* | nuclear receptor interacting protein 1 | 21q11.2-21.1 | Up | <0.01 | 2.7 |
| *RPL39* | ribosomal protein L39 | Xq24 | Up | <0.01 | 2.7 |
| *SNRPE* | small nuclear ribonucleoprotein polypeptide E | 1q32.1 | Up | <0.001 | 2.6 |
| *C2orf48* | chromosome 2 open reading frame 48 | 2p25.1 | Up | <0.01 | 2.6 |
| *LIN9* | lin-9 DREAM MuvB core complex component | 1q42.12 | Up | <0.01 | 2.6 |
| *RBM34* | RNA binding motif protein 34 | 1q42.3 | Up | <0.001 | 2.6 |
| *SNHG8* | small nucleolar RNA host gene 8 | 4q26 | Up | <0.01 | 2.5 |
| *ACBD3* | acyl-CoA binding domain containing 3 | 1q42.12 | Up | <0.001 | 2.5 |
| *FBXO28* | F-box protein 28 | 1q42.11 | Up | <0.001 | 2.5 |
| *RABGGTB* | Rab geranylgeranyltransferase beta subunit | 1p31.1 | Up | <0.01 | 2.4 |
| *CENPL* | centromere protein L | 1q25.1 | Up | <0.01 | 2.4 |
| *SEMA3F* | semaphorin 3F | 3p21.31 | Up | <0.01 | 2.4 |
| *EXO1* | exonuclease 1 | 1q43 | Up | <0.01 | 2.4 |
| *CRIM1* | cysteine rich transmembrane BMP regulator 1 | 2p22.2 | Up | <0.01 | 2.4 |
| *ANGEL2* | angel homolog 2 | 1q32.3 | Up | <0.001 | 2.4 |
| *TRIM23* | tripartite motif containing 23 | 5q12.3 | Up | <0.01 | 2.4 |
| *ZNF567* | zinc finger protein 567 | 19q13.12 | Up | <0.01 | 2.4 |
| *CENPN* | centromere protein N | 16q23.2 | Up | <0.01 | 2.4 |
| *RP9P* | RP9 pseudogene | 7p14.3 | Up | <0.01 | 2.4 |
| *PPP2R5A* | protein phosphatase 2 regulatory subunit B'alpha | 1q32.3 | Up | <0.01 | 2.4 |
| *NFE2L2* | nuclear factor, erythroid 2 like 2 | 2q31.2 | Up | <0.01 | 2.3 |
| *PSMC6* | proteasome 26S subunit, ATPase 6 | 14q22.1 | Up | <0.01 | 2.3 |
| *ZC3H11A* | zinc finger CCCH-type containing 11A | 1q32.1 | Up | <0.01 | 2.3 |
| *KLHL20* | kelch like family member 20 | 1q25.1 | Up | <0.001 | 2.3 |
| *GAS5* | growth arrest specific 5 (non-protein coding) | 1q25.1 | Up | <0.01 | 2.3 |
| *WDR26* | WD repeat domain 26 | 1q42.11-42.12 | Up | <0.01 | 2.3 |
| *DHX9* | DExH-box helicase 9 | 1q25.3 | Up | <0.01 | 2.3 |
| *NUP54* | nucleoporin 54 | 4q21.1 | Up | <0.01 | 2.3 |
| *TADA1* | transcriptional adaptor 1 | 1q24.1 | Up | <0.01 | 2.3 |
| *CNST* | consortin, connexin sorting protein | 1q44 | Up | <0.01 | 2.3 |
| *RAB3GAP2* | RAB3 GTPase activating non-catalytic protein subunit 2 | 1q41 | Up | <0.01 | 2.3 |
| *TRIM11* | tripartite motif containing 11 | 1q42.13 | Up | <0.01 | 2.3 |
| *RCOR3* | REST corepressor 3 | 1q32.2-32.3 | Up | <0.001 | 2.3 |
| *MATR3* | matrin 3 | 5q31.2 | Up | <0.01 | 2.3 |
| *ZNF678* | zinc finger protein 678 | 1q42.13 | Up | <0.01 | 2.3 |
| *SDHAP1* | succinate dehydrogenase complex flavoprotein subunit A pseudogene 1 | 3q29 | Up | <0.01 | 2.2 |
| *B3GALNT2* | beta-1,3-N-acetylgalactosaminyltransferase 2 | 1q42.3 | Up | <0.01 | 2.2 |
| *TRAF5* | TNF receptor associated factor 5 | 1q32.3 | Up | <0.01 | 2.2 |
| *SEC61A2* | Sec61 translocon alpha 2 subunit | 10p14 | Up | <0.01 | 2.2 |
| *EIF2A* | eukaryotic translation initiation factor 2A | 3q25.1 | Up | <0.01 | 2.2 |
| *DUSP12* | dual specificity phosphatase 12 | 1q23.3 | Up | <0.01 | 2.2 |
| *CRNKL1* | crooked neck pre-mRNA splicing factor 1 | 20p11.23 | Up | <0.01 | 2.2 |
| *CDC73* | cell division cycle 73 | 1q31.2 | Up | <0.001 | 2.2 |
| *NENF* | neudesin neurotrophic factor | 1q32.3 | Up | <0.01 | 2.2 |
| *MTF2* | metal response element binding transcription factor 2 | 1p22.1 | Up | <0.01 | 2.2 |
| *ZBTB18* | zinc finger and BTB domain containing 18 | 1q44 | Up | <0.0001 | 2.2 |
| *ELOC* | elongin C | 8q21.11 | Up | <0.01 | 2.2 |
| *LPGAT1* | lysophosphatidylglycerol acyltransferase 1 | 1q32.3 | Up | <0.01 | 2.1 |
| *HCFC2* | host cell factor C2 | 12q23.3 | Up | <0.01 | 2.1 |
| *FXR1* | FMR1 autosomal homolog 1 | 3q26.33 | Up | <0.01 | 2.1 |
| *DESI2* | desumoylating isopeptidase 2 | 1q44 | Up | <0.001 | 2.1 |
| *THOC1* | THO complex 1 | 18p11.32 | Up | <0.01 | 2.1 |
| *ZNF75A* | zinc finger protein 75a | 16p13.3 | Up | <0.01 | 2.1 |
| *TPR* | translocated promoter region, nuclear basket protein | 1q31.1 | Up | <0.01 | 2.1 |
| *FYTTD1* | forty-two-three domain containing 1 | 3q29 | Up | <0.01 | 2.1 |
| *TMEM159* | transmembrane protein 159 | 16p12.3 | Up | <0.01 | 2.1 |
| *RNF2* | ring finger protein 2 | 1q25.3 | Up | <0.01 | 2.0 |
| *TMEM57* | transmembrane protein 57 | 1p36.11 | Up | <0.01 | 2.0 |
| *SEC63* | SEC63 homolog, protein translocation regulator | 6q21 | Up | <0.01 | 2.0 |
| *GABPB1* | GA binding protein transcription factor beta subunit 1 | 15q21.2 | Up | <0.01 | 2.0 |
| *SRP72* | signal recognition particle 72 | 4q12 | Up | <0.01 | 2.0 |
| *MIA3* | MIA family member 3, ER export factor | 1q41 | Up | <0.001 | 2.0 |
| *KPNA4* | karyopherin subunit alpha 4 | 3q25.33 | Up | <0.01 | 2.0 |
| *GNL2* | G protein nucleolar 2 | 1p34.3 | Up | <0.01 | 2.0 |
| *EFNA3* | ephrin A3 | 1q21.3 | Up | <0.01 | 2.0 |
| *MRPL55* | mitochondrial ribosomal protein L55 | 1q42.13 | Up | <0.01 | 2.0 |
| *CNOT2* | CCR4-NOT transcription complex subunit 2 | 12q15 | Up | <0.01 | 2.0 |
| *CKAP2L* | cytoskeleton associated protein 2 like | 2q14.1 | Up | <0.01 | 2.0 |
| *C16orf91* | chromosome 16 open reading frame 91 | 16p13.3 | Up | <0.01 | 2.0 |
| *TTC13* | tetratricopeptide repeat domain 13 | 1q42.2 | Up | <0.01 | 2.0 |
| *DNM1L* | dynamin 1 like | 12p11.21 | Up | <0.01 | 2.0 |
| *TOR3A* | torsin family 3 member A | 1q25.2 | Up | <0.01 | 1.9 |
| *BDH2* | 3-hydroxybutyrate dehydrogenase 2 | 4q24 | Up | <0.01 | 1.9 |
| *NUP133* | nucleoporin 133 | 1q42.13 | Up | <0.01 | 1.9 |
| *OPA1* | OPA1, mitochondrial dynamin like GTPase | 3q29 | Up | <0.01 | 1.9 |
| *CDC27* | cell division cycle 27 | 17q21.32 | Up | <0.01 | 1.9 |
| *C3orf17* | nucleolus and neural progenitor protein | 3q13.2 | Up | <0.01 | 1.9 |
| *CEP57* | centrosomal protein 57 | 11q21 | Up | <0.01 | 1.9 |
| *XPR1* | xenotropic and polytropic retrovirus receptor 1 | 1q25.3 | Up | <0.01 | 1.9 |
| *C1orf35* | chromosome 1 open reading frame 35 | 1q42.13 | Up | <0.001 | 1.9 |
| *PPP4R2* | protein phosphatase 4 regulatory subunit 2 | 3p13 | Up | <0.01 | 1.9 |
| *WAC* | WW domain containing adaptor with coiled-coil | 10p12.1 | Up | <0.01 | 1.9 |
| *ADIPOR1* | adiponectin receptor 1 | 1q32.1 | Up | <0.01 | 1.9 |
| *DHX40* | DEAH-box helicase 40 | 17q23.1 | Up | <0.01 | 1.9 |
| *HEATR1* | HEAT repeat containing 1 | 1q43 | Up | <0.01 | 1.9 |
| *VAMP4* | vesicle associated membrane protein 4 | 1q24.3 | Up | <0.01 | 1.9 |
| *UBXN2A* | UBX domain protein 2A | 2p23.3 | Up | <0.01 | 1.9 |
| *ECD* | ecdysoneless cell cycle regulator | 10q22.2 | Up | <0.01 | 1.9 |
| *METAP2* | methionyl aminopeptidase 2 | 12q22 | Up | <0.01 | 1.8 |
| *SUZ12* | SUZ12 polycomb repressive complex 2 subunit | 17q11.2 | Up | <0.01 | 1.8 |
| *ATP13A3* | ATPase 13A3 | 3q29 | Up | <0.01 | 1.8 |
| *DDX59* | DEAD-box helicase 59 | 1q32.1 | Up | <0.001 | 1.8 |
| *TRIM24* | tripartite motif containing 24 | 7q33-34 | Up | <0.01 | 1.8 |
| *RPS6KC1* | ribosomal protein S6 kinase C1 | 1q32.3 | Up | <0.01 | 1.8 |
| *RAD21* | RAD21 cohesin complex component | 8q24.11 | Up | <0.001 | 1.8 |
| *HNRNPA1* | heterogeneous nuclear ribonucleoprotein A1 | 12q13.13 | Up | <0.01 | 1.8 |
| *PAIP2* | poly(A) binding protein interacting protein 2 | 5q31.2 | Up | <0.01 | 1.8 |
| *YME1L1* | YME1 like 1 ATPase | 10p12.1 | Up | <0.01 | 1.8 |
| *ZFP1* | ZFP1 zinc finger protein | 16q23.1 | Up | <0.01 | 1.8 |
| *CSDE1* | cold shock domain containing E1 | 1p13.2 | Up | <0.01 | 1.8 |
| *GLUL* | glutamate-ammonia ligase | 1q25.3 | Up | <0.01 | 1.8 |
| *FAM177A1* | family with sequence similarity 177 member A1 | 14q13.2 | Up | <0.0001 | 1.8 |
| *USP46* | ubiquitin specific peptidase 46 | 4q12 | Up | <0.01 | 1.8 |
| *UTP23* | UTP23, small subunit processome component | 8q24.11 | Up | <0.01 | 1.8 |
| *PIAS2* | protein inhibitor of activated STAT 2 | 18q21.1 | Up | <0.01 | 1.7 |
| *TTF1* | transcription termination factor 1 | 9q34.13 | Up | <0.01 | 1.7 |
| *DIEXF* | digestive organ expansion factor homolog | 1q32.2 | Up | <0.01 | 1.7 |
| *LYRM1* | LYR motif containing 1 | 16p12.3 | Up | <0.01 | 1.7 |
| *SRGAP2* | SLIT-ROBO Rho GTPase activating protein 2 | 1q32.1 | Up | <0.01 | 1.7 |
| *CEP70* | centrosomal protein 70 | 3q22.3 | Up | <0.01 | 1.7 |
| *BAZ1A* | bromodomain adjacent to zinc finger domain 1A | 14q13.1-13.2 | Up | <0.01 | 1.7 |
| *SMG7* | SMG7, nonsense mediated mRNA decay factor | 1q25.3 | Up | <0.001 | 1.7 |
| *ENY2* | ENY2, transcription and export complex 2 subunit | 8q23.1 | Up | <0.01 | 1.7 |
| *NOL10* | nucleolar protein 10 | 2p25.1 | Up | <0.001 | 1.7 |
| *COPB1* | coatomer protein complex subunit beta 1 | 11p15.2 | Up | <0.01 | 1.7 |
| *RWDD4* | RWD domain containing 4 | 4q35.1 | Up | <0.01 | 1.7 |
| *PUM2* | pumilio RNA binding family member 2 | 2p24.1 | Up | <0.01 | 1.6 |
| *DYNC2LI1* | dynein cytoplasmic 2 light intermediate chain 1 | 2p21 | Up | <0.01 | 1.6 |
| *PPP2R2A* | protein phosphatase 2 regulatory subunit Balpha | 8p21.2 | Up | <0.01 | 1.6 |
| *IVNS1ABP* | influenza virus NS1A binding protein | 1q25.3 | Up | <0.01 | 1.6 |
| *TCEAL1* | transcription elongation factor A like 1 | Xq22.2 | Up | <0.01 | 1.6 |
| *CCT2* | chaperonin containing TCP1 subunit 2 | 12q15 | Up | <0.01 | 1.6 |
| *DHX15* | DEAH-box helicase 15 | 4p15.2 | Up | <0.001 | 1.6 |
| *SNW1* | SNW domain containing 1 | 14q24.3 | Up | <0.01 | 1.6 |
| *TCP1* | t-complex 1 | 6q25.3 | Up | <0.01 | 1.6 |
| *UBE3A* | ubiquitin protein ligase E3A | 15q11.2 | Up | <0.01 | 1.5 |
| *PPP2R3C* | protein phosphatase 2 regulatory subunit B''gamma | 14q13.2 | Up | <0.01 | 1.5 |
| *MMAA* | methylmalonic aciduria (cobalamin deficiency) cblA type | 4q31.21 | Up | <0.01 | 1.5 |
| *EIF3M* | eukaryotic translation initiation factor 3 subunit M | 11p13 | Up | <0.01 | 1.5 |
| *TRIM37* | tripartite motif containing 37 | 17q22 | Up | <0.01 | 1.5 |
| *KATNA1* | katanin catalytic subunit A1 | 6q25.1 | Up | <0.01 | 1.4 |
| *LUC7L2* | LUC7 like 2, pre-mRNA splicing factor | 7q34 | Up | <0.01 | 1.4 |
| *HNRNPK* | heterogeneous nuclear ribonucleoprotein K | 9q21.32 | Up | <0.01 | 1.4 |
| *SNX6* | sorting nexin 6 | 14q13.1 | Up | <0.01 | 1.4 |
| *MTRR* | 5-methyltetrahydrofolate-homocysteine methyltransferase reductase | 5p15.31 | Up | <0.01 | 1.4 |
| *PICALM* | phosphatidylinositol binding clathrin assembly protein | 11q14.2 | Up | <0.01 | 1.4 |
| *RCHY1* | ring finger and CHY zinc finger domain containing 1 | 4q21.1 | Up | <0.01 | 1.3 |
| *CAMK2G* | calcium/calmodulin dependent protein kinase II gamma | 10q22.2 | Down | <0.01 | 0.8 |
| *CDC37* | cell division cycle 37 | 19p13.2 | Down | <0.01 | 0.8 |
| *C10orf76* | chromosome 10 open reading frame 76 | 10q24.32 | Down | <0.01 | 0.7 |
| *BCL2L12* | BCL2 like 12 | 19q13.33 | Down | <0.01 | 0.7 |
| *NAA40* | N(alpha)-acetyltransferase 40, NatD catalytic subunit | 11q13.1 | Down | <0.01 | 0.7 |
| *ATXN7L3B* | ataxin 7 like 3B | 12q21.1 | Down | <0.01 | 0.7 |
| *DEF6* | DEF6, guanine nucleotide exchange factor | 6p21.31 | Down | <0.01 | 0.7 |
| *EIF2AK1* | eukaryotic translation initiation factor 2 alpha kinase 1 | 7p22.1 | Down | <0.01 | 0.7 |
| *PHKA2* | phosphorylase kinase regulatory subunit alpha 2 | Xp22.13 | Down | <0.01 | 0.7 |
| *TAF8* | TATA-box binding protein associated factor 8 | 6p21.1 | Down | <0.01 | 0.7 |
| *TXLNA* | taxilin alpha | 1p35.2 | Down | <0.01 | 0.7 |
| *ABHD14B* | abhydrolase domain containing 14B | 3p21.2 | Down | <0.01 | 0.7 |
| *MRPS27* | mitochondrial ribosomal protein S27 | 5q13.2 | Down | <0.01 | 0.7 |
| *EPB41* | Erythrocyte membrane protein band 4.1 | 1p35.3 | Down | <0.01 | 0.7 |
| *POLR1E* | RNA polymerase I subunit E | 9p13.2 | Down | <0.01 | 0.7 |
| *THOC5* | THO complex 5 | 22q12.2 | Down | <0.01 | 0.7 |
| *GNL3L* | G protein nucleolar 3 like | Xp11.22 | Down | <0.01 | 0.7 |
| *TUBGCP2* | tubulin gamma complex associated protein 2 | 10q26.3 | Down | <0.01 | 0.7 |
| *ANKRD27* | ankyrin repeat domain 27 | 19q13.11 | Down | <0.01 | 0.7 |
| *CTDSP2* | CTD small phosphatase 2 | 12q14.1 | Down | <0.01 | 0.6 |
| *FAM160A2* | family with sequence similarity 160 member A2 | 11p15.4 | Down | <0.01 | 0.6 |
| *HEXA* | hexosaminidase subunit alpha | 15q23 | Down | <0.01 | 0.6 |
| *PARP16* | poly(ADP-ribose) polymerase family member 16 | 15q22.31 | Down | <0.01 | 0.6 |
| *EIF4EBP2* | eukaryotic translation initiation factor 4E binding protein 2 | 10q22.1 | Down | <0.01 | 0.6 |
| *SMCR7L* | mitochondrial elongation factor 1 | 22q13.1 | Down | <0.01 | 0.6 |
| *SFXN5* | sideroflexin 5 | 2p13.2 | Down | <0.01 | 0.6 |
| *TIMELESS* | timeless circadian clock | 12q13.3 | Down | <0.01 | 0.6 |
| *LARP1* | La ribonucleoprotein domain family member 1 | 5q33.2 | Down | <0.01 | 0.6 |
| *MCU* | mitochondrial calcium uniporter | 10q22.1 | Down | <0.01 | 0.6 |
| *ACAA1* | acetyl-CoA acyltransferase 1 | 3p22.2 | Down | <0.001 | 0.6 |
| *PDCD4-AS1* | PDCD4 antisense RNA 1 | 10q25.2 | Down | <0.01 | 0.6 |
| *ACSF2* | acyl-CoA synthetase family member 2 | 17q21.33 | Down | <0.01 | 0.6 |
| *TMEM164* | transmembrane protein 164 | Xq23 | Down | <0.01 | 0.6 |
| *WASF2* | WAS protein family member 2 | 1p36.11 | Down | <0.01 | 0.6 |
| *ACAD10* | acyl-CoA dehydrogenase family member 10 | 12q24.12 | Down | <0.01 | 0.6 |
| *UBA1* | ubiquitin like modifier activating enzyme 1 | Xp11.3 | Down | <0.01 | 0.6 |
| *TRIM25* | tripartite motif containing 25 | 17q23.1 | Down | <0.01 | 0.6 |
| *UBE3B* | ubiquitin protein ligase E3B | 12q24.11 | Down | <0.01 | 0.6 |
| *SPI1* | Spi-1 proto-oncogene | 11p11.2 | Down | <0.01 | 0.6 |
| *NDRG3* | NDRG family member 3 | 20q11.23 | Down | <0.01 | 0.6 |
| *HEMK1* | HemK methyltransferase family member 1 | 3p21.31 | Down | <0.01 | 0.6 |
| *MMS19* | MMS19 homolog, cytosolic iron-sulfur assembly component | 10q24.1 | Down | <0.01 | 0.6 |
| *DROSHA* | drosha ribonuclease III | 5p13.3 | Down | <0.01 | 0.6 |
| *C19orf24* | chromosome 19 open reading frame 24 | 19p13.3 | Down | <0.01 | 0.6 |
| *TCOF1* | treacle ribosome biogenesis factor 1 | 5q32-33.1 | Down | <0.01 | 0.6 |
| *ARHGAP35* | Rho GTPase activating protein 35 | 19q13.32 | Down | <0.01 | 0.6 |
| *C22orf25* | transport and golgi organization 2 homolog | 22q11.21 | Down | <0.01 | 0.6 |
| *GSS* | glutathione synthetase | 20q11.22 | Down | <0.01 | 0.6 |
| *STIM1* | stromal interaction molecule 1 | 11p15.4 | Down | <0.01 | 0.6 |
| *TTC31* | TTC31 | 2p13.1 | Down | <0.01 | 0.6 |
| *SLC35E2* | solute carrier family 35 member E2 | 1p36.33 | Down | <0.01 | 0.6 |
| *ELOVL5* | ELOVL fatty acid elongase 5 | 6p12.1 | Down | <0.01 | 0.6 |
| *SLC25A42* | solute carrier family 25 member 42 | 19p13.11 | Down | <0.01 | 0.6 |
| *PARP4* | poly(ADP-ribose) polymerase family member 4 | 13q12.12 | Down | <0.01 | 0.5 |
| *YIPF1* | Yip1 domain family member 1 | 1p32.3 | Down | <0.01 | 0.5 |
| *HUWE1* | HECT, UBA and WWE domain containing 1, E3 ubiquitin protein ligase | Xp11.22 | Down | <0.01 | 0.5 |
| *GSTZ1* | glutathione S-transferase zeta 1 | 14q24.3 | Down | <0.01 | 0.5 |
| *TMEM129* | TMEM129: transmembrane protein 129 | 4p16.3 | Down | <0.01 | 0.5 |
| *NFRKB* | nuclear factor related to kappaB binding protein | 11q24.3 | Down | <0.01 | 0.5 |
| *TEX261* | testis expressed 261 | 2p13.3 | Down | <0.01 | 0.5 |
| *TPP1* | tripeptidyl peptidase 1 | 11p15.4 | Down | <0.01 | 0.5 |
| *C6orf89* | chromosome 6 open reading frame 89 | 6p21.2 | Down | <0.01 | 0.5 |
| *PIP4K2B* | phosphatidylinositol-5-phosphate 4-kinase type 2 beta | 17q12 | Down | <0.01 | 0.5 |
| *ATP5D* | ATP synthase, H+ transporting, mitochondrial F1 complex, delta subunit | 19p13.3 | Down | <0.01 | 0.5 |
| *GHDC* | GH3 domain containing | 17q21.2 | Down | <0.01 | 0.5 |
| *DCTN1* | dynactin subunit 1 | 2p13.1 | Down | <0.01 | 0.5 |
| *HDGFRP2* | HDGF like 2 | 19p13.3 | Down | <0.01 | 0.5 |
| *RALB* | RAS like proto-oncogene B | 2q14.2 | Down | <0.01 | 0.5 |
| *TMEM140* | transmembrane protein 140 | 7q33 | Down | <0.01 | 0.5 |
| *RAI1* | retinoic acid induced 1 | 17p11.2 | Down | <0.01 | 0.5 |
| *ARHGAP25* | Rho GTPase activating protein 25 | 2p13.3 | Down | <0.01 | 0.5 |
| *SCAMP2* | secretory carrier membrane protein 2 | 15q24.1 | Down | <0.01 | 0.5 |
| *ERCC2* | ERCC excision repair 2, TFIIH core complex helicase subunit | 19q13.32 | Down | <0.01 | 0.5 |
| *MAP3K5* | mitogen-activated protein kinase kinase kinase 5 | 6q23.3 | Down | <0.01 | 0.5 |
| *ABCC10* | ATP binding cassette subfamily C member 10 | 6p21.1 | Down | <0.01 | 0.5 |
| *PGM1* | phosphoglucomutase 1 | 1p31.3 | Down | <0.01 | 0.5 |
| *NCKAP1L* | NCK associated protein 1 like | 12q13.13-13.2 | Down | <0.01 | 0.5 |
| *UQCR11* | ubiquinol-cytochrome c reductase, complex III subunit XI | 19p13.3 | Down | <0.01 | 0.5 |
| *SMYD4* | SET and MYND domain containing 4 | 17p13.3 | Down | <0.01 | 0.5 |
| *LAS1L* | LAS1 like, ribosome biogenesis factor | Xq12 | Down | <0.01 | 0.5 |
| *MAOA* | monoamine oxidase A | Xp11.3 | Down | <0.01 | 0.5 |
| *RFFL* | ring finger and FYVE like domain containing E3 ubiquitin protein ligase | 17q12 | Down | <0.01 | 0.5 |
| *C5orf63* | chromosome 5 open reading frame 63 | 5q23.2 | Down | <0.01 | 0.5 |
| *SCLY* | selenocysteine lyase | 2q37.3 | Down | <0.01 | 0.5 |
| *CAMKK2* | calcium/calmodulin dependent protein kinase kinase 2 | 12q24.31 | Down | <0.01 | 0.5 |
| *ARAP1* | ArfGAP with RhoGAP domain, ankyrin repeat and PH domain 1 | 11q13.4 | Down | <0.01 | 0.5 |
| *THG1L* | tRNA-histidine guanylyltransferase 1 like | 5q33.3 | Down | <0.01 | 0.5 |
| *VPS33A* | VPS33A, CORVET/HOPS core subunit | 12q24.31 | Down | <0.01 | 0.5 |
| *MLEC* | malectin | 12q24.31 | Down | <0.01 | 0.5 |
| *PINK1* | PTEN induced putative kinase 1 | 1p36.12 | Down | <0.001 | 0.5 |
| *SLC36A1* | solute carrier family 36 member 1 | 5q33.1 | Down | <0.01 | 0.5 |
| *SLC25A15* | solute carrier family 25 member 15 | 13q14.11 | Down | <0.01 | 0.5 |
| *TPGS1* | tubulin polyglutamylase complex subunit 1 | 19p13.3 | Down | <0.01 | 0.5 |
| *MAVS* | mitochondrial antiviral signaling protein | 20p13 | Down | <0.01 | 0.5 |
| *GALM* | galactose mutarotase | 2p22.1 | Down | <0.01 | 0.5 |
| *POLR2E* | RNA polymerase II subunit E | 19p13.3 | Down | <0.01 | 0.5 |
| *DFFA* | DNA fragmentation factor subunit alpha | 1p36.22 | Down | <0.01 | 0.5 |
| *LOC100507373* | uncharacterized LOC100507373 | 19p13.12 | Down | <0.01 | 0.5 |
| *PPM1F* | protein phosphatase, Mg2+/Mn2+ dependent 1F | 22q11.22 | Down | <0.01 | 0.5 |
| *PICK1* | protein interacting with PRKCA 1 | 22q13.1 | Down | <0.01 | 0.5 |
| *NUDT16* | nudix hydrolase 16 | 3q22.1 | Down | <0.01 | 0.5 |
| *RETSAT* | retinol saturase | 2p11.2 | Down | <0.01 | 0.5 |
| *APOL2* | apolipoprotein L2 | 22q12.3 | Down | <0.01 | 0.4 |
| *URB1* | URB1 ribosome biogenesis 1 homolog (S. cerevisiae) | 21q22.11 | Down | <0.01 | 0.4 |
| *METTL7A* | methyltransferase like 7A | 12q13.12 | Down | <0.01 | 0.4 |
| *PTPRH* | protein tyrosine phosphatase, receptor type H | 19q13.4 | Down | <0.01 | 0.4 |
| *RHOBTB2* | Rho related BTB domain containing 2 | 8p21.3 | Down | <0.01 | 0.4 |
| *TBC1D4* | TBC1 domain family member 4 | 13q22.2 | Down | <0.01 | 0.4 |
| *TRANK1* | tetratricopeptide repeat and ankyrin repeat containing 1 | 3p22.2 | Down | <0.01 | 0.4 |
| *GLB1L* | galactosidase beta 1 like | 2q35 | Down | <0.01 | 0.4 |
| *FSTL3* | follistatin like 3 | 19p13.3 | Down | <0.01 | 0.4 |
| *ACSL5* | acyl-CoA synthetase long chain family member 5 | 10q25.2 | Down | <0.01 | 0.4 |
| *GBA2* | glucosylceramidase beta 2 | 9p13.3 | Down | <0.01 | 0.4 |
| *FHOD1* | formin homology 2 domain containing 1 | 16q22.1 | Down | <0.01 | 0.4 |
| *TLN1* | talin 1 | 9p13.3 | Down | <0.01 | 0.4 |
| *UNC45A* | unc-45 myosin chaperone A | 15q26.1 | Down | <0.01 | 0.4 |
| *POU2AF1* | POU class 2 associating factor 1 | 11q23.1 | Down | <0.01 | 0.4 |
| *CHMP7* | charged multivesicular body protein 7 | 8p21.3 | Down | <0.01 | 0.4 |
| *PRSS16* | protease, serine 16 | 6p22.1 | Down | <0.01 | 0.4 |
| *RCC1L* | RCC1 like | 7q11.23 | Down | <0.01 | 0.4 |
| *STK11* | serine/threonine kinase 11 | 19p13.3 | Down | <0.0001 | 0.4 |
| *MBD3* | methyl-CpG binding domain protein 3 | 19p13 | Down | <0.01 | 0.4 |
| *AXDND1* | axonemal dynein light chain domain containing 1 | 1q25.2 | Down | <0.01 | 0.4 |
| *APOL1* | apolipoprotein L1 | 22q12.3 | Down | <0.01 | 0.4 |
| *ACBD4* | acyl-CoA binding domain containing 4 | 17q21.31 | Down | <0.001 | 0.4 |
| *SUFU* | SUFU negative regulator of hedgehog signaling | 10q24.32 | Down | <0.01 | 0.4 |
| *SMUG1* | single-strand-selective monofunctional uracil-DNA glycosylase 1 | 12q13.13 | Down | <0.01 | 0.4 |
| *MX2* | MX dynamin like GTPase 2 | 21q22.3 | Down | <0.01 | 0.4 |
| *POLRMT* | RNA polymerase mitochondrial | 19p13.3 | Down | <0.01 | 0.4 |
| *ARHGAP45* | Rho GTPase activating protein 45 | 19p13.3 | Down | <0.001 | 0.3 |
| *FITM2* | fat storage inducing transmembrane protein 2 | 20q13.12 | Down | <0.01 | 0.3 |
| *FUT2* | fucosyltransferase 2 | 19q13.33 | Down | <0.01 | 0.3 |
| *SHROOM3* | shroom family member 3 | 4q21.1 | Down | <0.01 | 0.3 |
| *MUM1* | melanoma associated antigen (mutated) 1 | 19p13.3 | Down | <0.01 | 0.3 |
| *ABCA7* | ATP binding cassette subfamily A member 7 | 19p13.3 | Down | <0.01 | 0.3 |
| *MED16* | mediator complex subunit 16 | 19p13.3 | Down | <0.01 | 0.3 |
| *RGP1* | RGP1 homolog, RAB6A GEF complex partner 1 | 9p13.3 | Down | <0.01 | 0.3 |
| *P4HA2* | prolyl 4-hydroxylase subunit alpha 2 | 5q31.1 | Down | <0.01 | 0.3 |
| *ARID3A* | AT-rich interaction domain 3A | 19p13.3 | Down | <0.01 | 0.3 |
| *PCSK4* | proprotein convertase subtilisin/kexin type 4 | 19p13.3 | Down | <0.01 | 0.3 |
| *TNFAIP8L3* | TNF alpha induced protein 8 like 3 | 15q21.2 | Down | <0.01 | 0.3 |
| *ZNF831* | zinc finger protein 831 | 20q13.32 | Down | <0.01 | 0.3 |
| *FERMT1* | fermitin family member 1 | 20p12.3 | Down | <0.001 | 0.3 |
| *C19orf25* | chromosome 19 open reading frame 25 | 19p13.3 | Down | <0.01 | 0.3 |
| *NOX3* | NOX3 | 6q25.3 | Down | <0.01 | 0.2 |
| *VDR* | vitamin D receptor | 12q13.11 | Down | <0.01 | 0.2 |
| *CAV1* | caveolin 1 | 7q31.2 | Down | <0.01 | 0.2 |
| *C9orf125* | transmembrane protein 246 | 9q31.1 | Down | <0.01 | 0.2 |
| *SAMHD1* | SAM and HD domain containing deoxynucleoside triphosphate triphosphohydrolase 1 | 20q11.23 | Down | <0.01 | 0.2 |
| *LOC100499484* | SUGT1-1300002K09Rik pseudogene | 9q22.33 | Down | <0.01 | 0.2 |
| *MAMDC2* | MAM domain containing 2 | 9q21.12 | Down | <0.01 | 0.2 |
| *TEX11* | testis expressed 11 | Xp11 | Down | <0.001 | 0.2 |
| *ICAM5* | intercellular adhesion molecule 5 | 19p13.2 | Down | <0.01 | 0.2 |
| *BMPR1A* | bone morphogenetic protein receptor type 1A | 10q23.2 | Down | <0.01 | 0.2 |
| *GNAT2* | G protein subunit alpha transducin 2 | 1p13.3 | Down | <0.01 | 0.2 |
| *RPL34-AS1* | RPL34 antisense RNA 1 (head to head) | 4q25 | Down | <0.001 | 0.2 |
| *FAM86B2* | family with sequence similarity 86 member B2 | 8p23.1 | Down | <0.01 | 0.2 |
| *NLRP12* | NLR family pyrin domain containing 12 | 19q13.42 | Down | <0.01 | 0.2 |
| *SCML4* | Scm polycomb group protein like 4 | 6q21 | Down | <0.01 | 0.2 |
| *ANKRD53* | ankyrin repeat domain 53 | 2p13.3 | Down | <0.01 | 0.2 |
| *IGJ* | joining chain of multimeric IgA and IgM | 4q13.3 | Down | <0.01 | 0.2 |
| *ASPDH* | aspartate dehydrogenase domain containing | 19q13.33 | Down | <0.01 | 0.2 |
| *MCOLN2* | mucolipin 2 | 1p22.3 | Down | <0.01 | 0.2 |
| *CHSY3* | chondroitin sulfate synthase 3 | 5q23.3 | Down | <0.01 | 0.2 |
| *RNF157-AS1* | RNF157 antisense RNA 1 | 17q25.1 | Down | <0.01 | 0.1 |
| *LINC00839* | long intergenic non-protein coding RNA 839 | 10q11.21 | Down | <0.01 | 0.1 |
| *CYSLTR2* | cysteinyl leukotriene receptor 2 | 13q14.2 | Down | <0.01 | 0.1 |
| *KCNMA1* | potassium calcium-activated channel subfamily M alpha 1 | 10q22.3 | Down | <0.01 | 0.1 |
| *PCDHGA11* | protocadherin gamma subfamily A, 11 | 5q31.3 | Down | <0.01 | 0.1 |
| *RHOD* | ras homolog family member D | 11q13.2 | Down | <0.01 | 0.1 |
| *TRIL* | TLR4 interactor with leucine rich repeats | 7p14.3 | Down | <0.01 | 0.1 |
| *PRR15L* | proline rich 15 like | 17q21.32 | Down | <0.001 | 0.1 |
| *STX17-AS1* | STX17 antisense RNA 1 | 9q31.1 | Down | <0.01 | 0.1 |
| *CHAD* | chondroadherin | 17q21.33 | Down | <0.01 | 0.1 |
| *PRICKLE1* | prickle planar cell polarity protein 1 | 12q12 | Down | <0.001 | 0.1 |
| *WFDC21P* | WAP four-disulfide core domain 21, pseudogene | 17q23.1 | Down | <0.01 | 0.1 |
| *ADAM23* | ADAM metallopeptidase domain 23 | 2q33.3 | Down | <0.001 | 0.1 |
| *HRASLS2* | HRAS like suppressor 2 | 11q12.3 | Down | <0.01 | 0.1 |
| *ZNF157* | zinc finger protein 157 | Xp11.3 | Down | <0.001 | 0.1 |
| *LOC100128568* | Uncharacterized LOC100128568 | 19p13.3 | Down | <0.01 | 0.1 |
| *KLRC2* | killer cell lectin like receptor C2 | 12p13 | Down | <0.001 | 0.1 |
| *HOXB4* | homeobox B4 | 17q21.32 | Down | <0.01 | 0.1 |
