# Supplementary material for: Mutation, methylation, and gene expression profiles in dup(1q)-positive pediatric B-cell precursor acute lymphoblastic leukemia
Source: Leukemia. 2018 Mar 12;32(10):2117–25. doi: 10.1038/s41375-018-0092-2 (PMC6170391; doi:10.1038/s41375-018-0092-2)
Supplement: Supplementary file 9 — Supplementary Figures(DOCX 8893 kb) [file 41375_2018_92_MOESM9_ESM.docx]

**Supplementary Figures**


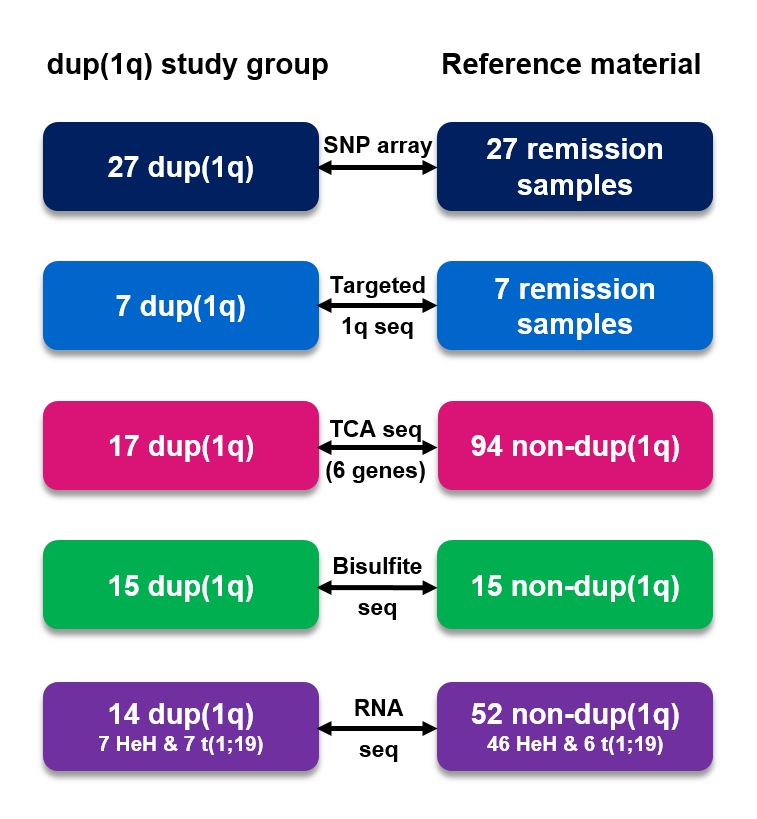


Supplementary Figure 1. Flowchart of the number of cases investigated in the various analyses in the present study. The left and right panels show the number of dup(1q)-positive and reference samples included in each analysis. HeH, high hyperdiploid cases (51-67 chromosomes); SNP, single nucleotide polymorphism; TCA, Truseq custom amplicon.


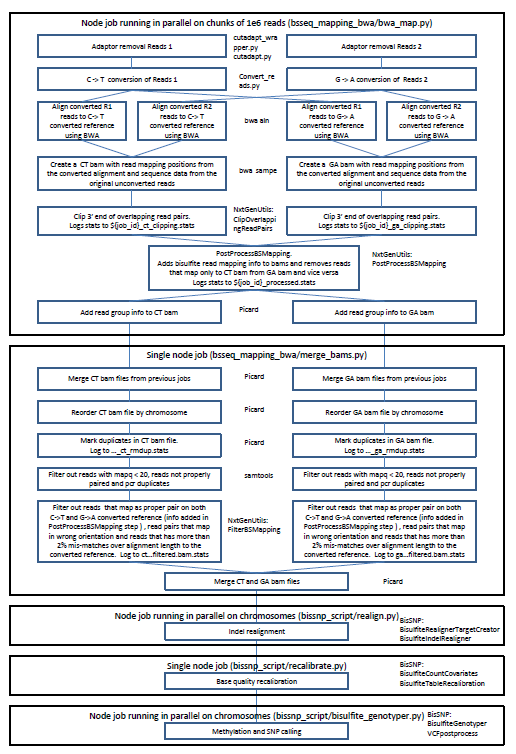


Supplementary Figure 2. Overview of the bisulfite sequencing pipeline.

**
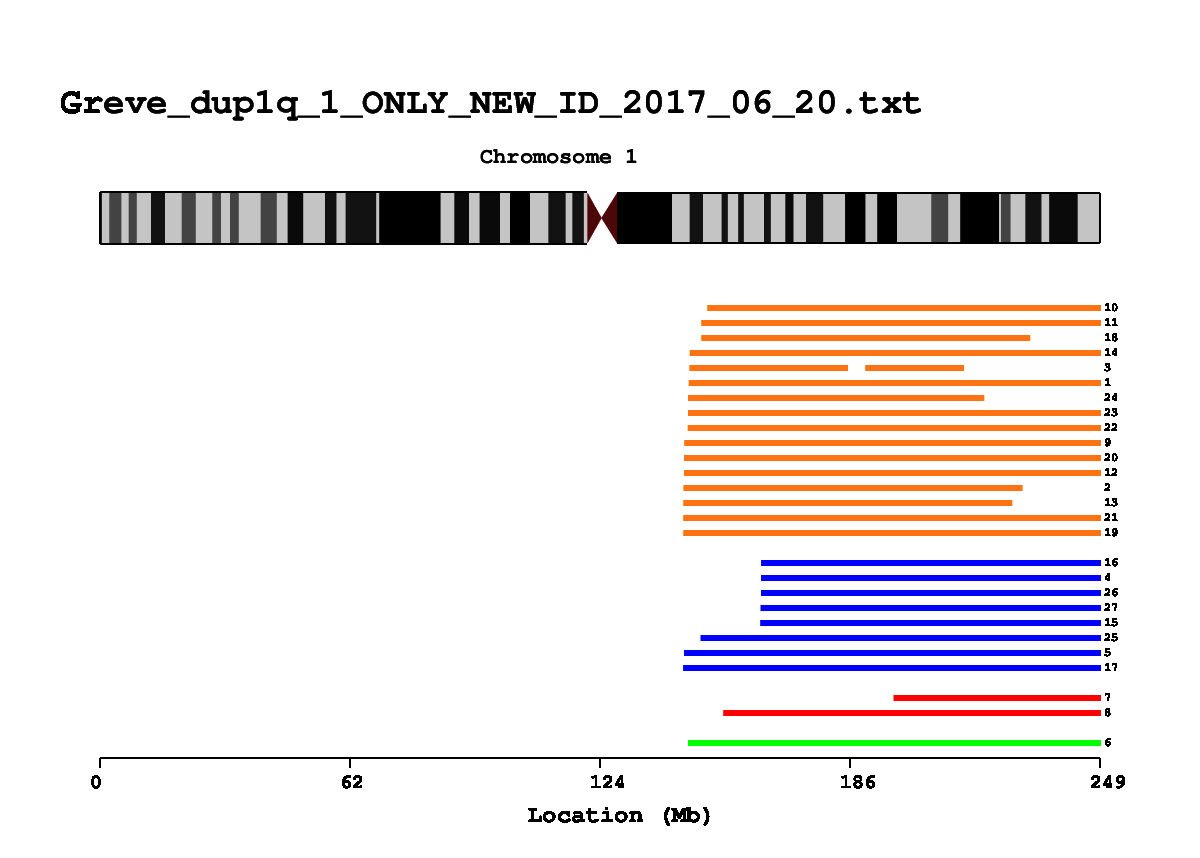
**

Supplementary Figure 3. Chromosome plot showing the extension of dup(1q) based on SNP-A analyses of 16 cases with high hyperdiploidy (51-67 chromosomes; orange lines), eight with *TCF3*-*PBX1* (t(1;19) cases; blue lines), two with B-other (red lines), and one with *ETV6*-*RUNX1* (green line).


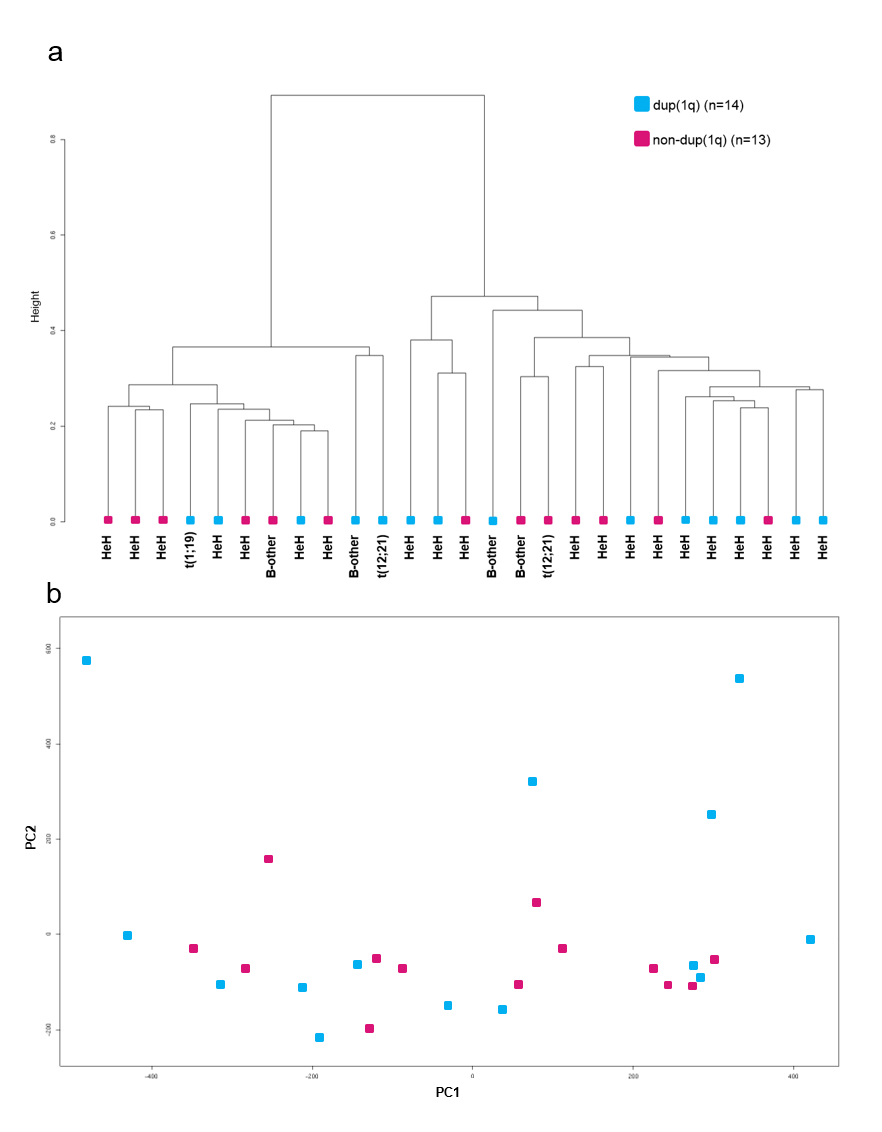


Supplementary Figure 4. Hierarchal clustering analysis (**a**) and principal component analysis (**b**) of the CpG methylation patterns on chromosome arm 1q in cases with (blue) and without (pink) dup(1q).


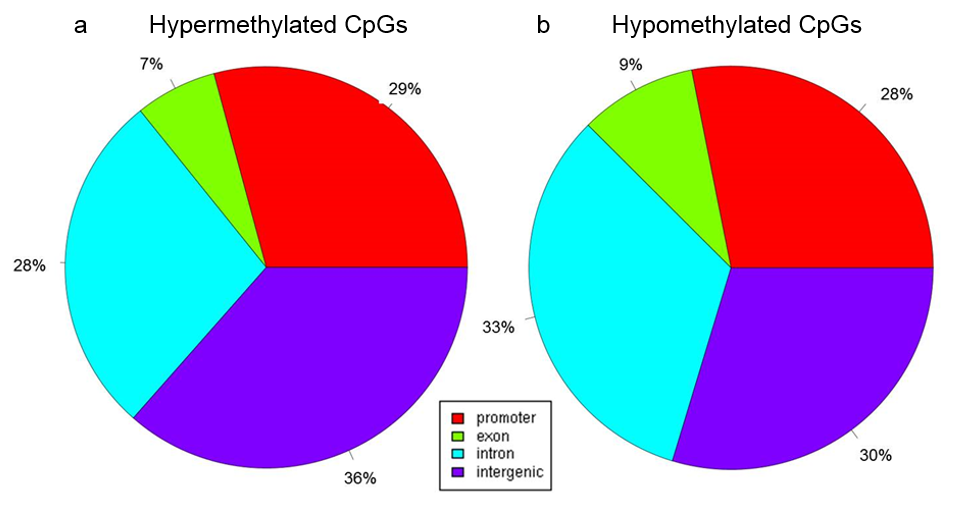


Supplementary Figure 5. Frequencies of differentially hypermethylated (**a**) and hypomethylated (**b**) CpGs in promoters, exons, introns, and intergenic regions on chromosome arm 1q in dup(1q)-positive cases.


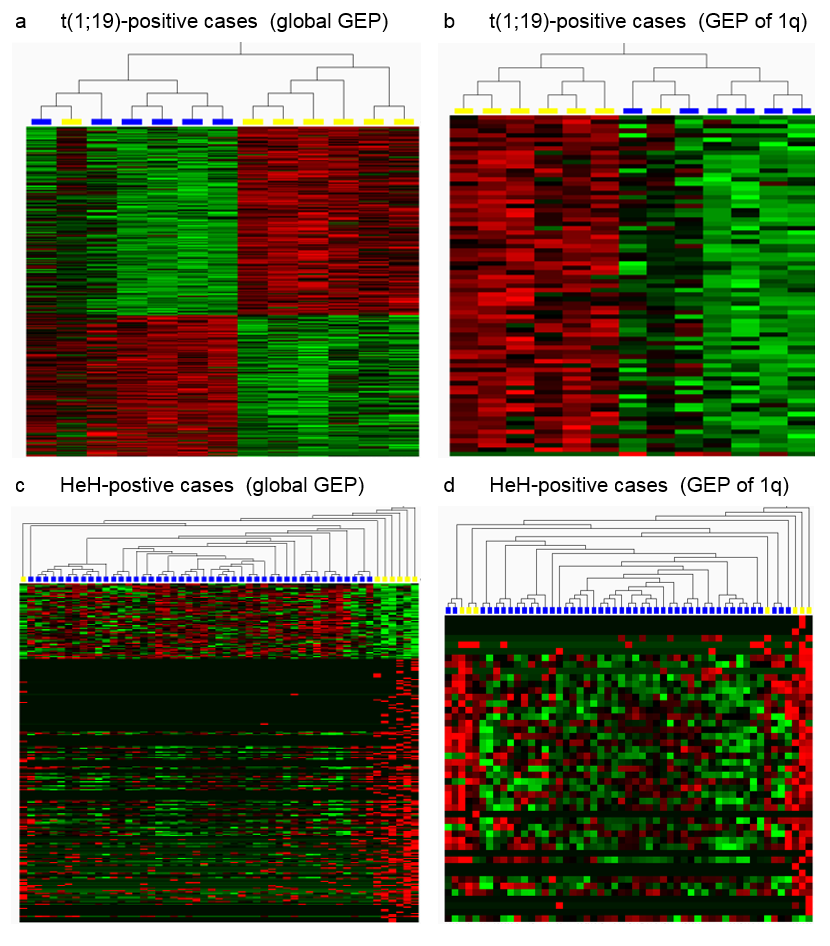


Supplementary Figure 6. Hierarchal clustering analysis of the global gene expression profiles (GEP) (**a, c**) and of the GEP of only genes mapping to 1q (**b, d**) in cases with t(1;19) (**a, b**) or with high hyperdiploidy (**c, d**). Cases w/wo dup(1q) are indicated in yellow and blue, respectively.


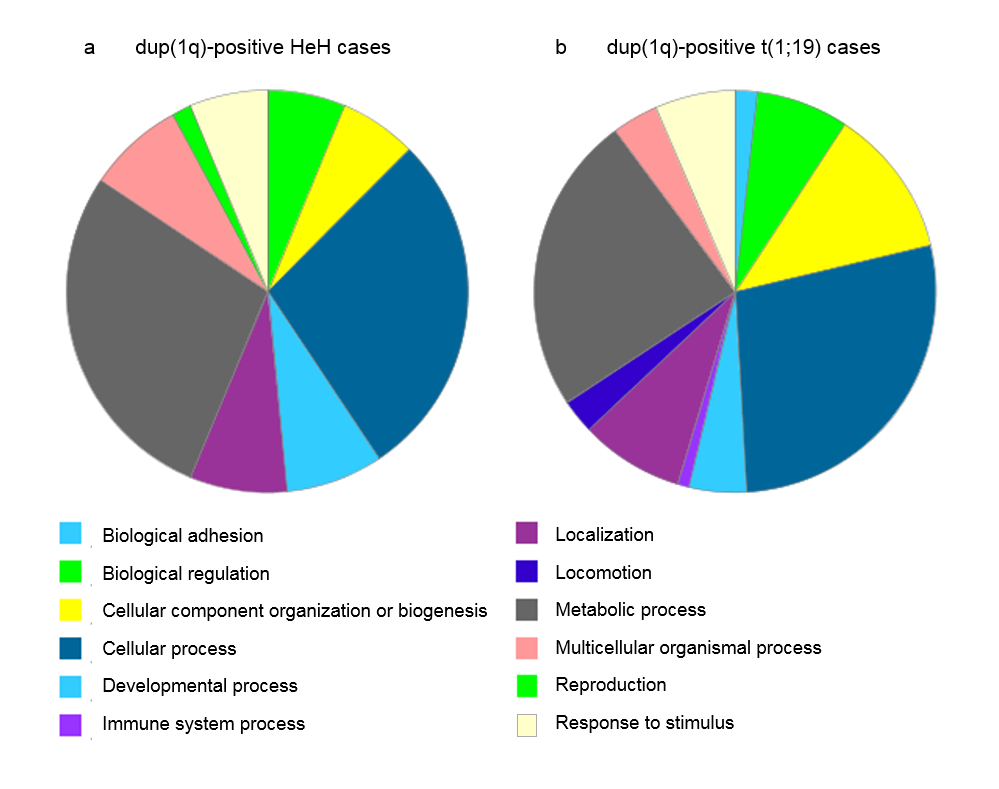


Supplementary Figure 7. Biological process ontology classes of genes on 1q upregulated in dup(1q)-positive cases. (**a**) In the HeH cases, 41 of the 46 upregulated genes had defined biological processes, comprising nine classes. (**b**) In the t(1;19) cases, 74 of the 76 overexpressed genes had defined biological processes, comprising 11 classes. Biological adhesion (GO:0022610); biological regulation (GO:0065007); cellular component organization or biogenesis (GO:0071840); cellular process (GO:0009987); developmental process (GO:0032502); immune system process (GO:0002376); localization (GO:0051179); locomotion (GO:0040011); metabolic process (GO:0008152); multicellular organismal process (GO:0032501); reproduction (GO:0000003); and response to stimulus (GO:0050896).


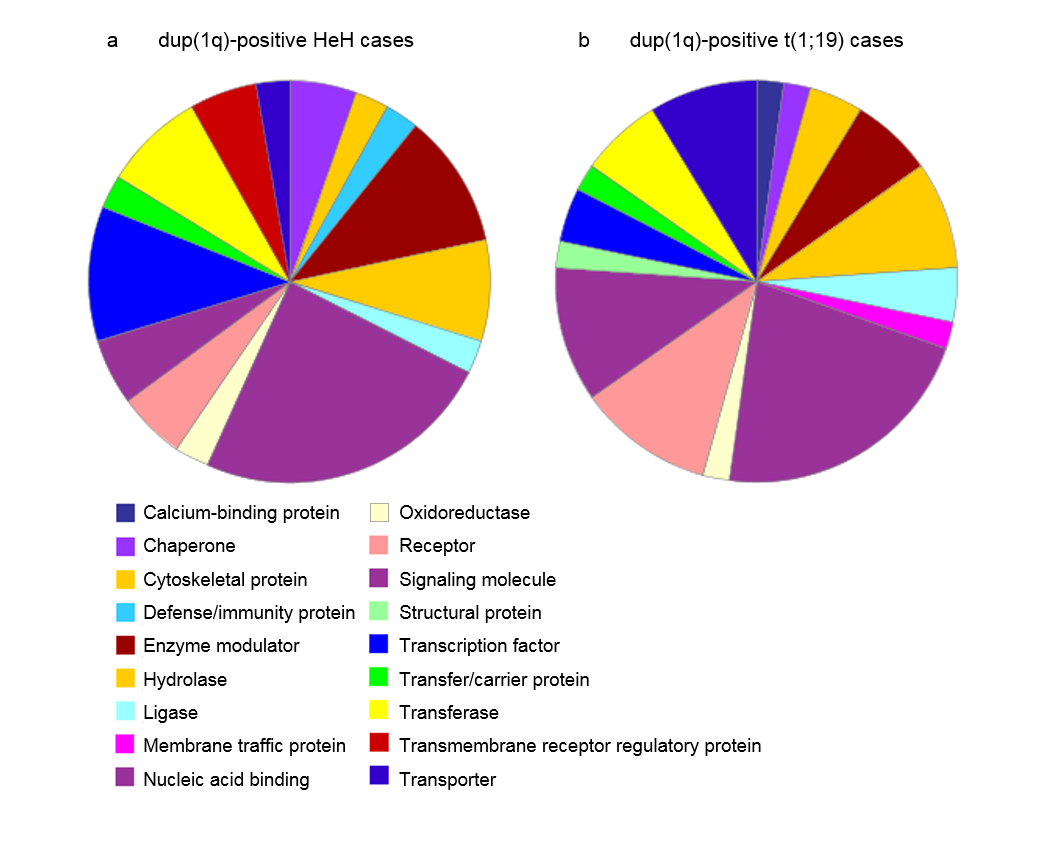
Supplementary Figure 8. Protein classes of genes on 1q upregulated in dup(1q)-positive cases. (**a**) In the HeH cases, 37 of the 46 upregulated genes had defined protein functions, comprising 15 classes. (**b**) In the t(1;19) cases, 46 of the 76 overexpressed genes had defined protein functions, comprising 16 classes. Calcium-binding protein (PC00060); chaperone (PC00072); cytoskeletal protein (PC00085); defense/immunity protein (PC00090); enzyme modulator (PC00095); hydrolase (PC00121); ligase (PC00142); membrane traffic protein (PC00150); nucleic acid binding (PC00171); oxidoreductase (PC00176); receptor (PC00197); signaling molecule (PC00207); structural protein (PC00211); transcription factor (PC00218); transfer/carrier protein (PC00219); transferase (PC00220); transmembrane receptor regulatory protein (PC00226), and transporter (PC00227).
